# A self-supervised reconstruction framework for segment-level detection of task-induced deviations in multi-channel EEG

**DOI:** 10.1371/journal.pone.0361586

**Published:** 2026-10-30

**Authors:** Lucas A. Saavedra, Antonio Quintero-Rincón, Hadj Batatia

**Affiliations:** 1 Laboratory of Molecular Neurobiology, Biomedical Research Institute (BIOMED), UCA-CONICET, Pontifical Catholic University of Argentina (UCA), Buenos Aires, Argentina; 2 Department of Data Science, Data Science and AI Laboratory, Faculty of Engineering and Agricultural Sciences, Pontifical Catholic University of Argentina (UCA), Buenos Aires, Argentina; 3 Department of Computer Sciences, Faculty of Engineering and Agricultural Sciences, Pontifical Catholic University of Argentina (UCA), Buenos Aires, Argentina; 4 MACS School, Heriot-Watt University, Dubai Campus, Dubai, United Arab Emirates; Karadeniz Technical University: Karadeniz Teknik Universitesi, TÜRKIYE

## Abstract

Understanding brain activations during mental arithmetic tasks provides valuable insights into cognitive processing. However, analyses of the same electroencephalogram (EEG) dataset have produced inconsistent findings about the cortical regions involved, raising methodological questions about global signal treatment. Detecting task-related activations without pre-labelled datasets further remains challenging when supervised approaches are not applicable. This study presents a self-supervised framework for segment-based anomaly detection using deep learning to identify EEG signal outliers during mental arithmetic. Using a hierarchical variational auto-encoder, the study reconstructed multichannel EEG data segmented into 1-second intervals and trained exclusively on resting-state recordings. The study assessed reconstruction quality using multiple error metrics, including dynamic time warping, short-time Fourier transform loss, and signal-to-noise ratio, applied individually to channel segments. Outlier segments were detected based on the elevated reconstruction error relative to the resting baseline, using an unsupervised detection method. By analysing deviations at the segment level rather than treating the signal globally, the proposed framework captured transient cortical dynamics that may otherwise be obscured. The results demonstrated that these outlier segments exhibited distinct temporal and spectral characteristics, particularly within the delta, theta, and beta bands, and were most frequently located in peripheral electrodes such as Fp2, O1, O2, P3, and T6. Additionally, co-occurrence analysis uncovered functional patterns of simultaneous anomalies across specific electrode pairs and triplets, suggesting temporally dynamic and spatially distributed cognitive processes during arithmetic reasoning. Overall, the proposed framework provided a fine-grained temporal and spatial characterisation of EEG deviations during mental arithmetic and offers a methodological perspective that helped explain discrepancies in previously reported electrode-level findings.

## 1. Introduction

Mental arithmetic involves controlled and explicit thinking. Controlled thinking involves activity in (among others) the dorsolateral prefrontal cortex (DLPFC), the dorsal premotor cortex (dPMC), and the posterior parietal cortex (PPC) [1,2]. During mathematical reasoning, these cortices interact with each other, playing distinct roles. DLPFC and dPMC, both located in the frontal lobes, are mainly related to working memory (WM), where information is stored short-term unless attention is continuously maintained [3]; and the intra-parietal sulcus (IPS), located in the PPC, builds a visuospatial representation of numerical quantities and interacts with the fusiform gyrus (FG) that decodes visual number forms [4]. Whereas thinking occurs between FG and IPS, short-term memory for the serial arithmetic task is carried out in the DLPFC and dPMC (regions directly related to WM). In addition, IPS interacts with WM. The interaction between these areas involves a consciousness process [1].

WM has been argued to play a key role in mental calculation [3,5]. The left parietal cortex [6], the left frontal area [7], and the frontal lobes [5] are the primary regions activated during mental arithmetic tasks [3]. In cases of incorrect arithmetic responses, fMRI analyses revealed activations in the DLPFC (i.e., BA 46) and the VLPFC (i.e., BA 47) [8]. A segment-based traditional analysis with monitored response timing—allowing precise identification of when responses occur—reported increased neural activity during mental calculation at 3.9 Hz (within the delta band) in Broca’s area, which is responsible for speech production [9], and at 5.46 Hz (within the theta band) in the prefrontal cortex, which is involved in attention-demanding tasks [3,10].

EEG signals have been used to investigate dynamic interactions among different cortical areas of the brain during mental arithmetic tasks. EEG is a non-invasive neurophysiological technique that assesses the brain’s electrical activity by attaching electrodes to the scalp. Its superior temporal resolution makes it an indispensable tool for examining real-time brain activity in various cognitive tasks, including mental arithmetic. In this domain, participants are instructed to solve basic arithmetic problems that require focused mental effort (e.g., subtracting large numbers). During the performance of these tasks, the electrodes placed on the participants’ scalps capture the electrical signals generated by synaptic activity within the involved brain regions [11], which are stored for analysis.

Shpenkov et al. [12] have assembled a dataset that exemplifies this type of experimental setting, including recordings from 36 subjects during both resting states and various arithmetic tasks. The recordings were analysed using Detrended Fluctuation Analysis (DFA) to elucidate long-term correlations in the signals and facilitate the identification of the principal regions activated during the task [13]. In later work [14], the same dataset was re-analysed using decision trees to determine the frequency bands that most accurately represent the underlying process. In [15], Khanam et al. questioned the way of analyzing the same data. They used Support Vector Machine (SVM) and other Machine Learning (ML) models to distinguish rest and task state signals separately for good and bad student performers. Their results showed that good performers exhibit ambiguous rest and task states as compared to bad performers. Another study [16] later focused on the same dataset. They used a multilayer Perceptron (MLP) in conjunction with a SVM to globally identify electrodes that exhibit significant differentiation between rest and task states, as well as between proficient and less proficient counters. The authors reported 98.4% accuracy in classifying good counters in both task and rest states. They also reported prominent signal changes in *F*_4_, *C*_4_, *T*_4_, *T*_5_, *P*_3_, and *O*_2_ areas, with significant changes in bad counters. In a more recent work [17], the same dataset from [12] was again analysed using three feature-based ML models Linear Discriminant Analysis (LDA), k-Nearest Neighbour (kNN), and SVM to distinguish between signals of proficient and less proficient counters, and kNN yielded the best F1-score of 0.857.

The three studies [15–17] address a fundamentally different problem from the one considered here. Specifically, they train supervised classifiers on globally extracted features to discriminate between a priori defined conditions such as rest versus task or good- versus poor-counter performance, and they report conventional supervised learning metrics such as classification accuracy or F1-score. These evaluation measures do not match the unsupervised, segment-level reconstruction-error scores produced by the framework proposed in this work, which relies on neither a predefined classification objective nor access to ground-truth outlier labels. Consequently, a direct quantitative comparison with these studies would conflate results obtained under distinct problem formulations and evaluation metrics and therefore would not constitute a meaningful benchmark.

All these analyses report different discrepancies between rest and task states in good and bad counters. However, despite using the same dataset, the reported discriminative electrodes and classification performances vary substantially across studies. These inconsistencies raise an important methodological question: why does the same dataset lead to different conclusions regarding the neural regions involved in mental arithmetic?

We argue that these inconsistencies stem from analysing the signal globally, which obscures immediate short-term variations and masks transient cortical dynamics. Most previous approaches treat the EEG signal as a single unit or rely on global feature extraction, potentially overlooking local temporal deviations that occur during arithmetic processing. Consequently, region-specific activation patterns may be diluted when averaged over long time intervals.

This observation motivates our approach to differentiate rest and task signals at the segment level without assuming prior knowledge of task-specific signals. Instead of analysing the entire signal, this paper focuses on the local scale using 1-second segments, enabling detection of short-term deviations from resting-state dynamics.

Previous studies have predominantly relied on global features or analyses performed over relatively long EEG intervals. Although such approaches can identify broad differences between participant groups or experimental conditions, they provide limited temporal resolution and may obscure transient, electrode-specific deviations. This limitation motivates the use of short, non-overlapping segments in the present work.

Deep Learning (DL)-based approaches improve the accuracy of EEG analysis by approximately ~5% compared to traditional methods [18]. Most efforts to develop DL methods in this area have concentrated on supervised and unsupervised learning techniques [18,19]. Although supervised learning generally outperforms unsupervised approaches [19], the latter benefits from not requiring pre-labelled datasets, which are unavailable in the dataset used in this work [12]. A subclass of unsupervised learning is self-supervised learning, which relies on automatic labelling and enables learning exclusively from unlabelled experimental EEG signals [20].

In particular, self-supervised learning can be used for anomaly detection by training neural networks on EEG data assumed to represent only normal activity. The trained network then reconstructs EEG signals recorded during the arithmetic tasks, and segments that are poorly reconstructed are considered abnormal relative to the resting baseline [21,22]. In this framework, anomalies reflect statistically significant deviations from resting-state dynamics rather than predefined task labels [23].

Auto-encoders detect anomalies by comparing the original signal to its reconstruction, where large reconstruction errors are considered outliers [22]. In EEG analysis, anomaly detection auto-encoders have been used to accurately identify seizures by analysing reconstruction errors in 1-second non-overlapping segments [22] and to detect artefacts using hierarchical architectures [24]. In [24], the hierarchical reconstruction model hvEEGNet was customised to each subject through dedicated training on resting-state EEG and then used to reconstruct signals recorded during an arithmetic task. We then identified deviations associated with task-related anomalies using DTW.

This study proposes a new label-free, reconstruction-based framework for detecting physiologically plausible task-induced EEG deviations at the segment and channel levels using an exclusively resting-state reference distribution. Unlike conventional anomaly-detection approaches that primarily identify corrupted, saturated, or otherwise invalid signals, the proposed framework treats task-related EEG activity as statistically deviant when its reconstruction-error profile differs sufficiently from the distribution learned during rest.

The framework comprises four methodological components. First, each EEG segment is represented by a multi-metric reconstruction-error vector comprising DTW, SNR, STFTL, Mean Absolute Percentage Error (MAPE), normalised correlation (NC), and Percent Root Mean Square Difference (PRD). These metrics capture complementary temporal, spectral, amplitude, correlation, and relative-error characteristics. Second, detection is performed at the segment level, enabling identification of short-duration deviations rather than obscuring them through global or trial-level aggregation. Third, a one-class detector is trained exclusively on resting-state EEG, without task-condition labels. Fourth, channel-level co-occurrence analysis determines whether detected deviations arise simultaneously across electrodes and characterises their spatial organisation during mental arithmetic.

The hvEEGNet architecture serves as the reconstruction backbone. The methodological contribution therefore lies not in modifying hvEEGNet itself, but in transforming its reconstruction errors into a multidimensional, segment-level, one-class detection and spatial co-occurrence framework for task-induced EEG deviations.

The channel-wise formulation is motivated by the fact that task-induced deviations from the resting-state distribution may arise in only a subset of channels within a given temporal segment. Aggregating reconstruction-error metrics from all channels into a single representation, as in multichannel formulations such as that considered by Cisotto et al. [24], would increase feature-space dimensionality as additional metrics are incorporated. This may obscure spatially localised deviations and make characterising normal observations more difficult, particularly in distance- and neighbourhood-based anomaly-detection methods [25–27]. In this study, EEG segments identified as outliers from their reconstruction-error profiles are termed anomalies in a strictly statistical sense; the term does not denote clinical abnormality. Accordingly, the terms *anomaly*, *outlier*, and *task-induced deviation* refer to physiologically plausible EEG segments that differ significantly from the resting-state reference distribution. Section 2.2.5 details the methodological rationale for channel-wise detection, including the implications of dimensionality reduction.

The study has two related objectives. First, it aims to identify short-duration, task-induced deviations from resting-state EEG at the segment and channel levels. Second, it aims to characterise the spatial and temporal organisation of these deviations by analysing their co-occurrence across electrodes during mental arithmetic.

We hypothesise that task-related deviations do not necessarily arise synchronously across all cortical regions but may occur in different subsets of electrodes at different times. To test this hypothesis, we first identify segment-level deviations relative to the resting-state reference distribution, then analyse their cross-channel co-occurrence patterns. This approach provides a dynamic perspective on cortical engagement during mental arithmetic that complements conventional global or trial-level EEG analysis.

The remainder of the paper is organised as follows. Material and Methods in Section 2 describes the experimental dataset (Section 2.1), and the proposed methodology (Section 2.2), where the following topics are addressed: the EEG signal segmentation (Section 2.2.1), the EEG signal reconstruction using hvEEGNet (Section 2.2.2), the reconstruction metrics (Section 2.2.3), the power spectral density (Section 2.2.4), the outlier clustering-based detection method (Section 2.2.5), the time-frequency co-occurrence analysis (Section 2.2.6), the experimental setup and design rationale (Section 2.2.7), the synthetic data generation procedure (Section 2.2.8), and the statistical analysis framework (Section 2.2.9). Section 3 presents the results and discussion, and Section 4 concludes the paper with a summary and future directions.

## 2. Materials and methods

### 2.1. Dataset

The public ICA-cleaned EEG dataset of Mental Arithmetic Task Performance, provided in [12], was considered for experimental purposes. This dataset includes 36 subjects performing serial subtraction of two numbers: a 4-digit minuend and a 2-digit subtrahend. Participants were 36 healthy university students (20 males, 16 females; mean age ≈20±1.5 years). The 19 electrodes of a standard 10–20 system (see Supplementary S1 Fig) were placed on the subjects’ scalp. The recordings consist of two parts: (i) the resting state and (ii) the mental counting. Before recording the resting state, subjects were instructed to relax, and rules regarding the task (e.g., finger movement is not allowed) were communicated. After this adaptation, the subjects closed their eyes, and a 3-minute recording began to capture resting-state signals. Next, subjects performed mental counting for 4 minutes, during which they calculated multiple subtractions. However, only the first minute of the arithmetic task was recorded to prevent cognitive overload (see Supplementary S2 Fig). Subjects were divided into “good” and “poor” quality counters following the definition provided by the original dataset authors and based on arithmetic task performance, whereby participants who required disproportionately high effort to complete the mental task were classified as poor counters. Specifically, participants were divided into Group “G” (24 subjects), characterised by good quality performance (mean number of operations per 4 minutes = 21, SD = 7.4), and Group “B” (12 subjects), characterised by poor-quality performance (mean number of operations per 4 minutes = 7, SD = 3.6). For more details on the experiment, see [12,28].

### 2.2. Methodology

This study aimed to identify electrodes that showed temporal deviations from the resting state during a mental-arithmetic task using a segment-level outlier-detection strategy. To achieve this, we developed a three-stage processing pipeline. First, we divided the EEG signal into non-overlapping 1-second segments. Resting-state segments served as the resting baseline (reference distribution) for subsequent modelling. Second, the hierarchical Variational Auto-encoder hvEEGNet was trained exclusively on resting-state segments to learn a subject-specific latent representation of normal (resting-state) EEG dynamics. Once trained, the model was used to reconstruct both resting-state and task-related segments. We quantified reconstruction quality using multiple metrics, including DTW, NC, MAPE, PRD, Short-Time Fourier Transform Loss (STFTL), and Signal-to-Noise Ratio (SNR). In the third stage, we compared task segments against the distribution of reconstruction errors from resting-state segments. We applied a self-supervised one-class learning method to identify segments that deviated significantly from resting-state dynamics. We then aggregated these segment-level outliers at the electrode level to identify channels that showed consistent task-related temporal deviations from the resting baseline.

Fig 1 summarises the full pipeline, including EEG signal acquisition and segmentation, training the hvEEGNet reconstruction model, and anomaly detection applied to EEG segments recorded during the mental-arithmetic task.

**Fig 1:**
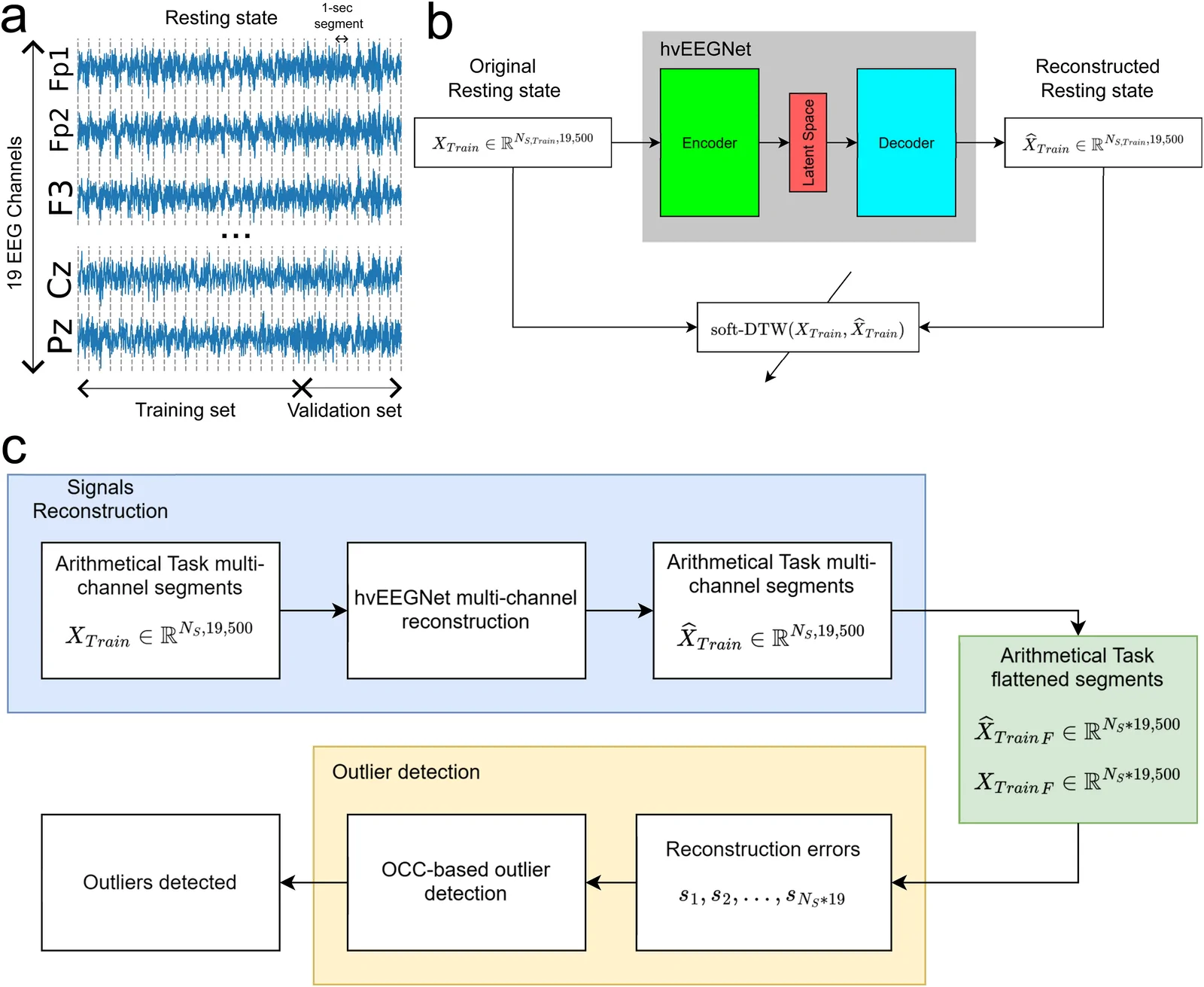
EEG reconstruction and outlier detection pipeline using hvEEGNet. **a.** Resting-state EEG recordings from 19 channels are segmented into 1-second windows and subsequently partitioned into training and validation sets. **b.** The hvEEGNet model, consisting of an encoder, latent space, and decoder, is trained to reconstruct resting-state EEG segments. The reconstruction error serves as the training loss, represented by the soft DTW (soft-DTW) loss between the original and reconstructed signals. **c.** After training, the model is used to reconstruct multi-channel EEG segments recorded during a mental arithmetic task. These segments are then flattened across channels to allow independent outlier detection per channel. We compute reconstruction errors for each segment and apply an OCC algorithm to identify outliers.

Fig 1a illustrates the EEG recordings from 19 distinct channels during the resting state. The EEG data are divided into 1-second segments and then split into training and validation sets. This segmentation is used to train a reconstruction model on normal (resting state) EEG activity.

Fig 1b illustrates the training of a separate reconstruction model for each subject. hvEEGNet processes the training set ${\hat{X}}_{Train}$ through the encoder to output the latent representation. The decoder reconstructs the signals from the latent representation. During training, the soft-DTW loss compares the reconstructed signals with the original signals to assess reconstruction quality. Once the hvEEGNet model was trained on resting-state EEG data, it was used to reconstruct EEG recordings obtained during the mental arithmetic task, as illustrated in Fig 1c. We first reconstructed the multi-channel task-related segments using the trained hvEEGNet, then reorganised them to enable independent outlier detection at the single-channel (i.e., electrode) level, ensuring that anomalies in one channel do not affect others. We then computed reconstruction errors by comparing the original and reconstructed EEG segments using the metrics DTW, NC, MAPE, PRD, STFTL, and SNR. In other words, we converted reconstruction errors for each channel segment into 6-dimensional data points (i.e., each value representing a different metric). These errors served as features for outlier detection using a One-Class Classification (OCC) model [29]. The detected outliers may correspond to atypical neural responses occurring during the mental-arithmetic task relative to the resting baseline.

The remainder of this section is organised as follows. We first describe the signal segmentation procedure and the multichannel reconstruction framework based on hvEEGNet, including reconstruction metrics and complementary spectral analysis. We then introduce the segment-level deviation modelling strategy based on single-channel reconstruction-error features and one-class learning. Next, we present connectivity and co-occurrence analyses to characterise inter-channel relationships associated with detected deviations. Finally, we detail the experimental setup and design rationale, followed by the synthetic data generation procedure used for validation and the statistical analysis framework adopted for hypothesis testing.

#### 2.2.1. Signal segmentation.

This study proposes encoding single-channel segments into feature vectors to enable outlier detection at the single-channel level, ensuring that anomalies in one channel do not influence others. This approach differs from the encoding strategy used in Cisotto et al. [24], where a single feature vector represents multiple channels jointly.

**Segment definition.** Let $X\in {\mathbb{R}}^{C\times T}$ denote the raw multi-channel EEG matrix, where each of the *C* rows corresponds to one EEG channel and the *T* columns represent time samples. Let $x_{c}\in {\mathbb{R}}^{1\times T}$ denote the time series corresponding to channel *c*, i.e., the *c*-th row of X.

The signal segmentation divides X into non-overlapping 1-second windows of length *N* samples using a rectangular window $\Omega_{i}\in {\mathbb{R}}^{T\times N}$ so that


$$\begin{array}{cc} x_{c,i} & =x_{c}\Omega_{i},\;x_{c,i}\in {\mathbb{R}}^{1\times N}, \end{array}$$ (1)

$$\begin{array}{cc} \Omega_{i} & =[\begin{array}{c} 0_{iN\times N}\\ I_{N\times N}\\ 0_{(T-iN-N)\times N} \end{array}], \end{array}$$ (2)

where $0_{a\times b}$ denotes the null matrix of size a×b, $I_{N\times N}$ is the identity matrix, *N* is the number of samples per 1-second segment, and *i* indexes the segment. We assume $i=0,1,...,\left\lfloor \frac{T}{N}\right\rfloor -1$. Note that the multiplication in Eq. 1 is well-defined since $x_{c}\in {\mathbb{R}}^{1\times T}$ and $\Omega_{i}\in {\mathbb{R}}^{T\times N}$.

The corresponding multi-channel segment is obtained by vertically stacking the single-channel segments:


$$\begin{array}{cc} X_{i} & =[\begin{array}{c} x_{1,i}\\ x_{2,i}\\ \vdots \\ x_{C,i} \end{array}]\in {\mathbb{R}}^{C\times N}. \end{array}$$ (3)

Equivalently, the multi-channel segment can be written in matrix form as


$$\begin{array}{c} X_{i}=X\Omega_{i}\in {\mathbb{R}}^{C\times N}, \end{array}$$

which is equivalent to stacking the segmented channel vectors defined in Eq. (1). In this work, the encoder is applied to each single-channel segment $x_{c,i}$ independently.

We selected a 1-second segment length, corresponding to *N* = 500 samples at a recording frequency of 500 Hz, as a compromise between temporal localisation and the amount of signal information available for reconstruction. Shorter segments provide finer temporal resolution but contain fewer samples, which may limit the reconstruction model’s ability to capture the EEG signal’s temporal and spectral structure. Conversely, longer segments may improve frequency resolution but reduce temporal specificity and increase the likelihood of violating local stationarity assumptions [30–34].

**Segment-length selection.** This fixed-length segmentation serves a different purpose from the temporal windows used in Detrended Fluctuation Analysis (DFA). DFA evaluates signal fluctuations over multiple temporal scales to characterise scaling behaviour, temporal persistence, and long-range correlations [13,35,36]. For example, Seleznov et al. [13] illustrate part of the DFA procedure using segments of length *N* = 200, but this value does not represent a fixed segmentation length for their complete EEG analysis. In contrast, the present framework requires fixed-length raw EEG segments with sufficient information for hvEEGNet reconstruction and subsequent calculation of temporal, spectral, amplitude, correlation, and relative-error metrics. Using 1-second segments therefore retains short-term temporal specificity while providing enough signal information for reconstruction-based deviation modelling.

**Overlap strategy.** EEG recordings may be divided into non-overlapping or overlapping epochs before extracting temporal, spectral, non-linear, or machine-learning features.

Non-overlapping segmentation divides the EEG signal into consecutive windows without reusing samples across epochs. This avoids the additional dependence introduced by overlapping windows, although adjacent segments may still remain temporally correlated because of the intrinsic autocorrelation of EEG signals. Because each sample contributes to only one epoch, non-overlapping segmentation also reduces the risk of inflating the effective sample size and limits data leakage in machine-learning pipelines. Its main drawback is that, for a fixed recording duration, it yields fewer epochs than overlapping segmentation, which may increase the variability of spectral estimates and reduce the temporal density with which transient neurophysiological changes can be tracked.

Overlapping segmentation reuses subsets of EEG samples across consecutive epochs, thereby increasing the number of analysable segments and improving temporal continuity. Overlap is also commonly employed between internal sub-windows in spectral-estimation methods such as Welch’s method to reduce the variability of power spectral density estimates. More generally, overlapping windows are widely used in spectral analysis, time–frequency decomposition, and real-time brain–computer interfaces. However, direct sample reuse introduces additional dependence between neighbouring epochs. If overlapping segments derived from the same recording are distributed across training, validation, or test sets without appropriate separation, model performance may be substantially overestimated because of data leakage.

In this work, we adopted non-overlapping 1-second windows (see Eqs. (1)–(3)) and implemented them as fixed-length epochs with zero temporal overlap. This choice was made for three reasons specific to the proposed pipeline: (i) it avoids direct sample reuse across segments and limits the additional dependence that would be introduced by overlapping windows, thereby reducing dependence between observations used for model fitting, validation, and evaluation; (ii) it reduces the risk of data leakage between the segments used to train hvEEGNet and those held out for validation or evaluated during the arithmetic task; and (iii) the resulting reduction in the number of epochs is not a practical limitation in this study, since each subject contributes approximately 150 non-overlapping resting-state segments for training, while the synthetic validation described in Section 2.2.8 provides approximately 19,000 generated segments per class. In addition, we trained five independently initialised hvEEGNet models per subject to reduce variability associated with model initialisation.

**Temporal binning.** Temporal binning combines adjacent samples through averaging or another aggregation operation [31,37]. In EEG analysis, binning may reduce high-frequency noise and improve the signal-to-noise ratio [38]. From a signal-processing perspective, it acts as a smoothing or low-pass operation that suppresses rapid temporal fluctuations and reduces variance [39]. However, excessive binning may remove physiologically relevant transient events, reduce temporal specificity, and attenuate high-frequency spectral components, particularly in the beta and gamma bands [40–42].

This study applied no temporal binning, sample averaging, additional aggregation, or downsampling. Each 1-second segment retains the original sampling resolution of 500 Hz, corresponding to *N* = 500 samples, and hvEEGNet is trained and evaluated directly on these full-resolution segments. This choice avoids the loss of high-frequency content and temporal specificity that binning could introduce, which is important for both signal reconstruction and the subsequent spectral analyses described in (Sections 2.2.4 and 2.2.6).

**Boundary and spectral-window effects.** Edge effects may arise when finite EEG segments are subjected to spectral, wavelet, Hilbert-transform, or filtering procedures [41,43–46]. At segment boundaries, signal-continuity assumptions may be violated, producing spectral leakage, amplitude distortion, or spurious oscillatory components. These artefacts are particularly relevant to wavelet transforms and finite impulse response filters, whose convolution kernels extend beyond the available signal interval. Common mitigation strategies include signal padding, tapered analysis windows, mirror or symmetric extension, and removing boundary samples after filtering [31,39,41,47].

In our implementation, segmentation itself uses a rectangular (boxcar) window $\Omega_{i}$ with hard, non-overlapping boundaries (Eq. (2)), since hvEEGNet reconstructs each segment end-to-end and does not apply convolutional filtering across segment edges. Edge-effect mitigation is instead applied at the level of the spectral transforms computed on top of the reconstructed segments: power spectral density estimation for the SNR metric (Section 2.2.4) uses Welch’s method with a tapered flat-top window and 50% overlap between sub-windows, and the short-time Fourier transform used for the STFTL metric (Section 2.2.3) is computed with a tapered Gaussian analysis window. These tapered windows reduce spectral leakage at the boundaries of each 1-second segment relative to a rectangular window, at the cost of reduced effective spectral resolution.

#### 2.2.2. Multichannel EEG reconstruction using hvEEGNet.

Auto-encoders (AEs) provide a framework for learning compact representations of input signals. An AE consists of an encoder ϕ, which maps an input segment (either $X_{i}$ or $x_{c,i}$) into a lower-dimensional vector, and a decoder θ, which reconstructs the input from this code [48]. Formally, the encoder produces a latent representation


$$\begin{array}{c} z_{i}=\phi (X_{i}), \end{array}$$

and the decoder reconstructs the signal as


$$\begin{array}{c} {\hat{X}}_{i}=\theta (z_{i}). \end{array}$$

Variational Auto-encoders (VAEs) extend this framework by learning probability distributions rather than deterministic codes. Instead of producing a single latent vector, the encoder outputs the parameters of a conditional distribution $q_{\phi }(z_{i}|X_{i})$, while the decoder models the likelihood $p_{\theta }(X_{i}|z_{i})$ [49,50]. This formulation enables generative capabilities and imposes structure on the latent space. Training a VAE involves minimising the negative Evidence Lower Bound (ELBO), composed of a reconstruction term and a Kullback–Leibler (KL) divergence term. In the following, we introduce the notation in general form before specialising to hvEEGNet.

Hierarchical VAEs (HVAEs) generalise the VAE by introducing multiple layers of latent variables $z_{i}^{\left(1\right)},...,z_{i}^{\left(L\right)}$ arranged in a top-down generative hierarchy [51]. Higher-level latent variables capture global or slow-varying features, while lower levels encode finer details. The generative model factorizes as


$$\begin{array}{c} p(X_{i},z_{i}^{\left(1\right)},...,z_{i}^{\left(L\right)})=p(z_{i}^{\left(L\right)})\left(\prod_{\ell =1}^{L-1}p(z_{i}^{\left(\ell \right)}|z_{i}^{\left(\ell +1\right)})\;\right)p(X_{i}|z_{i}^{\left(1\right)}), \end{array}$$ (4)

with approximate posteriors of the form


$$\begin{array}{c} q_{\phi }(z_{i}^{\left(1\right)},...,z_{i}^{\left(L\right)}|X_{i})=q_{\phi }(z_{i}^{\left(1\right)}|X_{i})\prod_{\ell =2}^{L}q_{\phi }(z_{i}^{\left(\ell \right)}|z_{i}^{\left(\ell -1\right)}). \end{array}$$ (5)

hvEEGNet [24] is a hierarchical VAE specifically designed for multichannel EEG. Its input and output are multichannel 1-second segments $X_{i},{\hat{X}}_{i}\in {\mathbb{R}}^{C\times N}$, where *C* is the number of EEG channels and *N* is the number of time samples per segment (see Eq. (1)–(3)). The encoder of hvEEGNet maps each segment $X_{i}$ into a hierarchy of latent variables


$$\begin{array}{c} z_{i}^{\left(1\right)},...,z_{i}^{\left(L\right)},\;z_{i}^{\left(\ell \right)}\in {\mathbb{R}}^{d_{\ell }}, \end{array}$$

and the decoder reconstructs the segment using the hierarchical generative model.

The reconstruction quality is optimized using a differentiable dynamic time warping (soft-DTW) loss, which is robust to local temporal misalignments. The full training objective is


$$\begin{array}{c} {ℒ}_{\text{hvEEGNet}}={ℒ}_{\text{reconstruction}}+{ℒ}_{\text{KL}}, \end{array}$$ (6)

where ${ℒ}_{\text{reconstruction}}$ is the soft-DTW loss evaluated between the multichannel segments $X_{i}$ and ${\hat{X}}_{i}$ (see Section 2.2.3), and ${ℒ}_{\text{KL}}$ is the sum of KL divergence terms associated with all latent layers.

Importantly, we use hvEEGNet solely for multichannel reconstruction. Outlier detection in this work is performed separately (Section 2.2.5) using *single-channel* segments $x_{c,i}\in {\mathbb{R}}^{1\times N}$ (see Eq. (1)), ensuring that deviations detected in one channel do not influence the detection process in other channels. The mathematical structure of hvEEGNet remains fully compatible with this design, as reconstruction losses are computed at the multichannel level while anomaly detection operates on single-channel reconstruction-error representations derived from the multichannel reconstructions.

It is further worth noting that the hvEEGNet model is trained exclusively using the soft-DTW reconstruction loss. Although we later introduce additional reconstruction-error metrics for deviation analysis (Section 2.2.3), we do not incorporate them into the training objective. This deliberate design preserves independence between the reconstruction optimisation stage and the subsequent multi-metric deviation detection process, thereby preventing circular optimisation effects.

#### 2.2.3. Reconstruction metrics.

This section defines the metrics used to evaluate the accuracy and fidelity of a reconstructed single-channel EEG segment $\hat{x}*c,i\in {\mathbb{R}}^{1\times N}$ with respect to its original segment $x*c,i\in {\mathbb{R}}^{1\times N}$, as defined in Eq. (1). For notational simplicity, we write $x,\hat{x}\in {\mathbb{R}}^{1\times N}$ for a generic original–reconstructed pair. For multichannel segments $X_{i},{\hat{X}}_{i}\in {\mathbb{R}}^{C\times N}$, metrics are computed channel-wise and can subsequently be aggregated across channels.

**Dynamic Time Warping (DTW):** DTW measures similarity between two time series by finding an optimal temporal alignment [52]:


$$\begin{array}{c} \text{DTW}(x,\hat{x})=\min_{W}\sum_{(n,m)\in W}dist(x_{n},\hat{x}*m), \end{array}$$ (7)

where *W* is the warping path and dist(·,·) is typically the squared Euclidean distance. Because standard DTW is not differentiable, Cuturi and Blondel [53] introduced soft-DTW, a differentiable relaxation controlled by γ>0:


$$\begin{array}{c} \text{soft-DTW}(x,\hat{x})=-\gamma \log\sum *W\exp!\left(-\frac{cost(W)}{\gamma }\right). \end{array}$$ (8)

Here, cost(*W*) denotes the DTW path cost. We use the PyTorch implementation of soft-DTW provided for hvEEGNet [54–56].

**Normalized Correlation (NC):** NC measures the linear correlation between two time series [57,58]:


$$\begin{array}{c} \text{NC}(x,\hat{x})=\frac{\sum_{n=1}^{N}(x_{n}-\bar{x})(\hat{x}*n-\bar{\hat{x}})}{(N-1),\sigma *x,\sigma_{\hat{x}}}, \end{array}$$ (9)

where $\bar{x}$ and $\bar{\hat{x}}$ are the sample means and $\sigma_{x},\sigma_{\hat{x}}$ are the corresponding sample standard deviations. NC ranges from −1 to 1, with 1 indicating perfect agreement.

**Mean Absolute Percentage Error (MAPE):** MAPE measures the average absolute reconstruction error relative to the original signal [59]:


$$\begin{array}{c} \text{MAPE}(x,\hat{x})=\frac{100\%}{N}\sum_{n=1}^{N}\left|\frac{x_{n}-{\hat{x}}_{n}}{x_{n}}\right|, \end{array}$$ (10)

computed over indices for which $x_{n}\text{≠}0$. Lower values indicate better reconstruction, with zero denoting a perfect match.

**Percent Root Mean Squared Difference (PRD):** PRD measures reconstruction distortion relative to the energy of the original signal [60]:


$$\begin{array}{c} \text{PRD}(x,\hat{x})=100\times \frac{{\left|x-\hat{x}\right|}_{2}}{{\left|x\right|}_{2}}. \end{array}$$ (11)

Lower values indicate less distortion, with PRD = 0 for a perfect reconstruction.

**Short-time Fourier Transform loss (STFTL):** Following Défossez et al. [61], the STFT loss compares signals in the time–frequency domain using the magnitude of their short-time Fourier transforms (STFTs). Let S(x)=STFT(x) and $S(\hat{x})=STFT(\hat{x})\in {\mathbb{C}}^{F\times K}$ be the complex spectrograms of x and $\hat{x}$, with *F* frequency bins and *K* time frames. The STFT loss is defined as


$$\begin{array}{c} {ℒ}_{STFT}(x,\hat{x})=\frac{\|\;|S(x)|-|S(\hat{x})|\;{\|}_{F}}{\|\;|S(x)|\;{\|}_{F}}+\frac{1}{K}\;\|\log|S(x)|-\log|S(\hat{x})|{\|}_{1}, \end{array}$$ (12)

where |·| denotes the magnitude, $\|\cdot {\|}_{F}$ the Frobenius norm, and $\|\cdot {\|}_{1}$ the entry-wise $\ell_{1}$ norm. This loss emphasises spectral-content preservation and captures frequency-specific deviations that may not be reflected by time-domain measures.

**Signal-to-Noise Ratio (SNR):** SNR expresses the signal power relative to reconstruction-noise power [62]. For a single-channel segment,


$$\begin{array}{c} P(x)=\frac{1}{N}\sum_{n=1}^{N}x_{n}^{2},\;P_{\text{noise}}(x,\hat{x})=\frac{1}{N}\sum_{n=1}^{N}(x_{n}-\hat{x}*n{)}^{2}. \end{array}$$ (13)

The SNR (in decibels) is


$$\begin{array}{c} \text{SNR}(x,\hat{x})=10\log*10\left(\frac{P(x)}{P_{\text{noise}}(x,\hat{x})}\right). \end{array}$$ (14)

Higher SNR indicates better reconstruction, with SNR→∞ for a perfect match.

Table 1 summarises the reconstruction metrics under study.

**Table 1:** Reconstruction metrics.

Method	Type	Purpose	Metric range
DTW	Distance	Measures similarity allowing time misalignment	Lower is better (0 = perfect)
NC	Similarity	Measures linear correlation between signals	[−1,1] (1 = perfect)
MAPE	Error	Percentage error between original and reconstructed	Lower is better (0 = perfect)
PRD	Error	Signal distortion relative to original energy	Lower is better (0 = perfect)
STFTL	Frequency loss	Compares signals in time–frequency domain	Lower is better
SNR	Quality	Signal strength relative to reconstruction noise	Higher is better (→∞ = perfect)

Pairwise Pearson correlations among the six metrics were computed on the mental-arithmetic segments (Supplementary S7 Fig). PRD and STFTL showed the highest correlation (ρ=0.87), while the remaining 14 pairs had |ρ|<0.80. We retain all six metrics because PRD and STFTL capture complementary time-domain and time–frequency aspects of reconstruction.

#### 2.2.4. Power spectral density (PSD).

To analyse the frequency content of each single-channel segment, the power spectral density (PSD) is estimated using Welch’s method [63]. Let ${\hat{X}}_{i}\in {\mathbb{R}}^{C\times N}$ be the reconstructed multichannel segment corresponding to the original segment $X_{i}$. For a fixed segment index *i* and channel *c*, denote the reconstructed single-channel signal as ${\hat{x}}_{c,i}\in {\mathbb{R}}^{1\times N}$, corresponding to the single-channel segment defined in Eq. (1). Welch’s method partitions ${\hat{x}}_{c,i}$ into *K* possibly overlapping windows of length *M*, computes the discrete Fourier transform of each window, and averages the squared magnitudes.

For the full multichannel segment ${\hat{X}}_{i}$, the cross-spectral density (CSD) matrix at frequency *f* is defined as


$$\begin{array}{c} S_{{\hat{X}}_{i}}(f)=\frac{1}{K}\sum_{k=1}^{K}{\hat{X}}_{i,k}(f)\;{\hat{X}}_{i,k}(f{)}^{H}, \end{array}$$ (15)

where

- ${\hat{X}}_{i,k}(f)\in {\mathbb{C}}^{C\times 1}$ is the *C*-dimensional discrete Fourier transform (DFT) of the *k*-th window of ${\hat{X}}_{i}$,
- $(\cdot {)}^{H}$ denotes the Hermitian (conjugate) transpose,
- *K* is the number of windows used in Welch’s averaging.

The diagonal entries $S_{{\hat{X}}_{i}}(c,c;f)$ correspond to the PSD of channel *c*, while the off-diagonal entries $S_{{\hat{X}}_{i}}(c,d;f)$ represent the cross-spectral densities between channels *c* and *d*. In this work, a flat-top window is used with a segment length of *M* samples and an overlap of *M*/2 samples. For datasets with higher sampling rates, these parameters are scaled proportionally to maintain a fixed temporal window duration.

In this work, the PSD is not used as an additional reconstruction error metric in the outlier-detection pipeline. Instead, it serves as a complementary frequency-domain analysis tool applied after segment-level outlier detection to characterise the spectral content of the reconstructed EEG segments. By examining how power is distributed across canonical EEG bands (e.g., delta, theta, alpha, beta) for inlier and outlier segments, the PSD analysis provides a physiological interpretation of the detected segment-level deviations and of the differences between rest and task-related activity.

#### 2.2.5. Single-channel deviation modelling.

This stage identifies EEG segments that deviate from the resting-state baseline distribution and can therefore be considered segment-level outliers. Consistent with the segmentation notation introduced in Section 2.2.1, each single-channel EEG segment is denoted by $x_{c,i}\in {\mathbb{R}}^{1\times N}$, and its reconstruction, obtained from the multichannel hvEEGNet reconstruction and restricted to channel *c*, is denoted by ${\hat{x}}_{c,i}\in {\mathbb{R}}^{1\times N}$.

We adopt a channel-wise detection strategy rather than constructing a single feature vector containing reconstruction-error metrics from all channels. In a multichannel representation such as that considered by Cisotto et al. [24], adding error metrics increases the dimensionality of each observation with both the number of channels and the number of metrics. High-dimensional representations can make the structure of the normal-data distribution increasingly difficult to estimate, particularly in anomaly-detection and other unsupervised methods that depend on distances or local neighbourhoods [25,26]. This issue is especially relevant to EEG analysis, where the original signal representation is already highly dimensional [64]. As dimensionality increases, distances may become less discriminative, potentially affecting the distinction between normal and deviant observations [27].

Dimensionality-reduction techniques such as Principal Component Analysis (PCA) can produce a lower-dimensional multichannel representation [64]. PCA does not require deviations to occur simultaneously across all channels; however, because it projects channel-level information onto latent components that capture dominant global variance, it may attenuate or obscure deviations that are sparse, spatially localised, or present in only a subset of electrodes. Channel-wise modelling avoids this aggregation and preserves electrode-specific deviation profiles, while the subsequent co-occurrence analysis determines whether detected deviations arise concurrently across multiple channels.

Outlier detection is therefore performed independently for each channel *c*. The feature vector and outlier classification for channel *c* are computed solely from $\left(x_{c,i},{\hat{x}}_{c,i}\right)$, without using reconstruction-error information from any other channel. This procedural independence is guaranteed by construction, but it does not imply that the resulting outlier labels are statistically independent across channels. Two or more channels may be classified as deviant within the same segment because they respond to a common underlying cognitive event. Section 2.2.6 examines this empirical dependence by quantifying the co-occurrence structure of the channel-level outlier labels and testing it against a permutation-based null model of channel independence.

**Feature construction:** For every reconstructed single-channel segment $\left(x_{c,i},{\hat{x}}_{c,i}\right)$, we compute the reconstruction-error metrics defined in Section 2.2.3: soft-DTW (the differentiable DTW variant used in hvEEGNet training), NC, MAPE, PRD, STFTL, and SNR. These six metrics constitute a feature vector encoding the deviation of the reconstructed signal from its original counterpart. Formally, for channel *c* and segment *i*, we define


$$\begin{array}{c} \theta_{c,i}=(e_{c,i}^{\text{DTW}},e_{c,i}^{\text{NC}},e_{c,i}^{\text{MAPE}},e_{c,i}^{\text{PRD}},e_{c,i}^{\text{STFTL}},e_{c,i}^{\text{SNR}})\in {\mathbb{R}}^{6}, \end{array}$$ (16)

where each $e_{c,i}^{\left(\cdot \right)}$ is the error measure obtained from the corresponding metric applied to $\left(x_{c,i},{\hat{x}}_{c,i}\right)$.

The resulting feature vectors represent the multi-metric deviation profiles of individual segments and serve as inputs to the channel-specific outlier-detection models.

**One-class and unsupervised outlier detection:** Resting-state segments form a high-density region in the resting-state feature distribution, while task-related or statistically deviant segments tend to fall in low-density regions or isolated micro-clusters. A single-channel segment $x_{c,i}$ is classified as an outlier if its feature vector $\theta_{c,i}$ either (i) does not belong to any cluster, (ii) lies at a large distance from its nearest cluster centroid, or (iii) forms part of a very small or sparse cluster. This behaviour reflects deviations in reconstruction error, temporal alignment, or spectral characteristics.

**Comparison with existing approaches:** A previously used reconstruction-based outlier criterion is kNN distance thresholding [24], where a segment is labelled an outlier if its *k*-th nearest neighbour distance exceeds a predefined threshold *d*. This approach, however, requires subject-specific tuning of *d* and relies on validity indices (e.g., DBI) that assume distinct and well-separated clusters, an assumption not met in our data.

We therefore evaluated several unsupervised one-class methods, including one-class SVM (OCSVM) with an RBF kernel, Isolation Forest (IF), and Local Outlier Factor (LOF) [65]. For each channel, the models were fitted exclusively to its resting-state feature vectors $\theta_{c,i}$ and then used to assign an inlier or outlier score to each task-related single-channel segment.

No systematic hyperparameter search (e.g., grid search or cross-validation) was performed for OCSVM, IF, or LOF. Default scikit-learn configurations were applied uniformly across all subjects: OCSVM with an RBF kernel, ν=0.1, and γ=`scale'; IF with 100 estimators and automatic setting of contamination; and LOF with 20 neighbours and automatic setting of contamination [65].

Prior to fitting, the six reconstruction-error features were normalised (zero mean and unit variance; scikit-learn’s StandardScaler [65]). Statistics were estimated exclusively on the resting-state training data and then applied to all segments. This normalisation places the six error metrics on a common scale, preventing kernel- and distance-based detectors from being dominated by the largest-magnitude feature. We apply this normalisation throughout the anomaly-detection analyses reported in this work on real EEG data.

The resulting channel-level classifications provide fine-grained temporal deviation profiles that are subsequently aggregated to analyse spatial, electrode-level patterns relative to resting-state dynamics.

#### 2.2.6. Connectivity and co-occurrence analyses.

**Graph analysis of concurrent segment-level deviations:** To investigate connectivity between concurrent segment-level deviations across channels, we constructed weighted graphs based on the co-occurrence of segment-level deviations across channels, aggregated over subjects. For each subject *s*, a binary outlier-indicator matrix


$$\begin{array}{c} A^{\left(s\right)}\in \{0,1{\}}^{C\times I} \end{array}$$

was constructed, where *C* is the number of channels and *I* corresponds to the total number of segments defined in Section 2.2.1. The entry $A_{c,i}^{\left(s\right)}=1$ indicates that segment *i* of channel *c* was classified as an outlier, and 0 otherwise.

Pairwise co-occurrence counts between channels were then computed from $A^{\left(s\right)}$ by counting, for each segment *i*, all pairs (*c*,*d*) such that $A_{c,i}^{\left(s\right)}=1$ and $A_{d,i}^{\left(s\right)}=1$. Formally, the subject-level co-occurrence matrix is defined as


$$\begin{array}{c} C_{c,d}^{\left(s\right)}=\sum_{i=1}^{I}A_{c,i}^{\left(s\right)}A_{d,i}^{\left(s\right)}. \end{array}$$

These subject-level co-occurrence matrices were accumulated across subjects and normalised to obtain average connectivity values. Formally,


$$\begin{array}{c} C=\frac{1}{S}\sum_{s=1}^{S}C^{\left(s\right)}, \end{array}$$

where *S* denotes the total number of subjects.

To better account for relative overlap between channel activity profiles, we also computed a Jaccard-like index, defined as the ratio between the number of co-occurrence events (intersection) and the total number of anomalous events across the corresponding pair of channels (union). Formally, for channels *c* and *d*, the Jaccard weight is defined using the aggregated co-occurrence matrix C as


$$\begin{array}{c} J(c,d)=\frac{C_{c,d}}{C_{c,c}+C_{d,d}-C_{c,d}}. \end{array}$$ (17)

We used this index as the weight of the graph edges.

We constructed an undirected weighted graph where nodes represent channels and edges connect channel pairs with non-zero Jaccard weight. Co-occurrence links with a Jaccard weight below 0.3 were discarded for the analysis. Each graph was then analysed using standard network measures. In particular, we estimated weighted degree and detected community structure via the greedy modularity maximisation algorithm, while quantifying node centrality using weighted betweenness and weighted degree. We implemented the graph-based analysis using networkX in Python [66]. This graph-based analysis complements the time–frequency co-occurrence analysis described earlier by providing a structural representation of channel-level deviation patterns.

To determine whether the observed pairwise co-occurrences reflect genuine cross-channel coupling rather than chance overlap between independently fluctuating per-channel outlier rates, we constructed a permutation-based null model. For each subject, the temporal order of the binary outlier indicator sequence was independently shuffled for each channel (1,000 permutations), preserving each channel’s marginal outlier rate while destroying any temporal cross-channel coupling. Observed co-occurrence counts $C_{c,d}$ were compared against this null distribution, and the resulting per-pair p-values were corrected for multiple comparisons using the Benjamini–Hochberg FDR procedure (see Section 2.2.9). 97.1% of channel pairs exhibited co-occurrence significantly above the chance level expected under independent per-channel detection (FDR-corrected *p* < 0.05), confirming that the spatial co-occurrence patterns captured by the Jaccard-weighted graph described above reflect genuine, non-random cross-channel structure rather than an artefact of independently fluctuating per-channel detection rates.

**Time-Frequency co-occurrence analysis:** In contrast to the graph analysis based on binary segment-level deviations, the following analysis examines phase synchronisation patterns between channels within specific frequency bands.

Co-occurrence analysis is frequently used in EEG signal examination to identify patterns of functional brain connectivity derived from events or features in the EEG signal that occur concurrently or within a brief temporal window (or signal segmentation), either within a single signal or across multiple EEG channels. This method investigates the co-occurrence of oscillatory patterns characterised by brain rhythms within the delta, theta, and alpha frequency bands across different regions during arithmetic calculations, using the Phase Locking Value (PLV) metric for its quantification in a range of [0,1], where 1 is perfect phase locking,


$$\begin{array}{c} PLV_{c,d}^{\left(i\right)}=\left|\frac{1}{N}\sum_{t=1}^{N}\exp\;\left(i\left(\phi_{c,i}(t)-\phi_{d,i}(t)\right)\right)\right|, \end{array}$$ (18)

where $\phi_{c,i}(t)$ and $\phi_{d,i}(t)$ denote the instantaneous phases of the single-channel EEG segments $x_{c,i}$ and $x_{d,i}$, respectively, at time index *t*, obtained from the analytic signal via the Hilbert transform [67].

This transformation converts the real-valued signal $x_{c,i}(t)$ into an analytic signal, $z_{c,i}(t)$, from which the instantaneous phase $\phi_{c,i}(t)$ is subsequently derived as:


$$\begin{array}{c} z_{c,i}(t)=x_{c,i}(t)+i\;ℋ[x_{c,i}(t)] \end{array}$$ (19)

where the Hilbert transform $ℋ[x_{c,i}(t)]$ of $x_{c,i}(t)$, as well as the instantaneous phase $\phi_{c,i}(t)$, are defined as follows:


$$\begin{array}{cc} ℋ[x_{c,i}(t)] & =\frac{1}{\pi }\text{PV}\int_{-\infty }^{\infty }\frac{x_{c,i}(\tau )}{t-\tau }d\tau \end{array}$$ (20)

$$\begin{array}{cc} \phi_{c,i}(t) & =\arctan\left(\frac{ℋ[x_{c,i}(t)]}{x_{c,i}(t)}\right). \end{array}$$ (21)

The kernel in $\frac{1}{t-\tau }$ has a singularity at τ=t. We use the Cauchy Principal Value (PV) to address singularities. For further details on time-frequency co-occurrence analysis, refer to [68–70].

#### 2.2.7. Experimental setup and design rationale.

We evaluated the reconstructed signals ${\hat{X}}_{i}$ using the metrics described in Section 2.2.3. Each reconstructed signal corresponds to a 1-second resting-state segment, with *N* = 500 time points and *C* = 19 channels. Reconstructed segments serve as the validation set for the hvEEGNet method (Section 2.2.2).

In summary, for each subject, we trained five hvEEGNet models in a self-supervised fashion using different random initialisations to ensure robustness. Consequently, the signals from the resting state were exclusively employed to train the hvEEGNet network. We used the initial 2.5 minutes of the resting state for training, reserving the remaining time solely for validation. Furthermore, all recordings were segmented into non-overlapping 1-second segments (*N* = 500), (see Eq. (3)). Hence, the training dataset comprised approximately 150 segments; see Eq. (6).

As explained in the methodology section, the trained model served to reconstruct multichannel EEG segments recorded during a mental arithmetic task. We then processed these segments channel-wise to independently detect segment-level deviations per channel. The reconstruction errors for each segment were computed, and an OCC algorithm [29] was employed to identify segments that deviate from the resting-state baseline distribution. In this context, segments that deviate from the resting-state baseline distribution are referred to as outliers in the remainder of the paper. Accordingly, we adopted a single-channel strategy instead of a multi-channel approach.

#### 2.2.8. Synthetic data generation with hvEEGNet.

This experiment verifies that the one-class detectors correctly reject outlier points injected into the proposed feature space. It is not intended as evidence of sensitivity to physiologically realistic task-induced deviations.

To provide a controlled validation of the proposed methodology, synthetic resting-state signals were generated using the trained hvEEGNet model. Normal segments were generated by sampling latent variables from the learned prior distribution and decoding them through the generative model. Each generated sample corresponds to a multichannel segment $X_{i}\in {\mathbb{R}}^{C\times N}$. Abnormal segments were created by adding artificial perturbations 𝒫*i:


$$\begin{array}{c} \tilde{X}*i=X_{i}+{𝒫}_{i}. \end{array}$$ (22)

These perturbations consisted of sinusoidal components with random frequency (0–40 Hz), amplitude (0–10 μV), phase (0–2π), and vertical offset (–25–25 μV). For each channel *c*, ten sinusoidal components were added independently, producing clear deviations from normal EEG activity.

The validation is restricted to sinusoidal perturbations and is not intended to span the full range of deviation morphologies that could arise in practice; systematic comparison with additional perturbation families (e.g., wavelet-based transients, broadband or non-stationary colored noise, tapered oscillatory bursts) is left for future work.

For each subject, 19,000 normal and 19,000 abnormal samples were generated. We processed both groups through the same reconstruction and feature-extraction pipeline described in Sections 2.2.2 and 2.2.3 to calculate reconstruction errors. We saved the original and reconstructed signals for outlier-detection analysis.

#### 2.2.9. Statistical analysis.

To assess differences between distributions of reconstruction-error features and deviation-related measures, we used the non-parametric Mann–Whitney U test [71]. Depending on the hypothesis direction, we applied left- or right-tailed tests. We used *p* < 0.05 as the threshold for statistical significance. Given the large number of simultaneous tests across metrics, electrodes, frequency bands, and subjects, we adjusted all reported *p*-values for multiple comparisons using the Benjamini–Hochberg false discovery rate (FDR) procedure [72].

## 3. Results and discussion

In this section, we present results using a progressive analytical framework to characterise mental task–induced deviations from the resting-state baseline. We begin with a reconstruction-based analysis at the segment level to quantify temporal and spectral discrepancies. We then use these reconstruction-error features to validate a statistical anomaly-detection approach, where deviations are defined relative to the resting distribution.

We subsequently examine the prevalence of task-induced deviations, followed by a spatial analysis across EEG channels and a co-occurrence analysis to assess inter-electrode interactions. This hierarchical approach—from segment-level errors to spatial and network-level patterns—provides a multi-scale characterisation of cognitive load effects in EEG signals.

### 3.1. Reconstruction analysis

This subsection reports the reconstruction performance of the proposed hvEEGNet-based framework (Section 2.2) on the EEG dataset described in Section 2.1. We illustrate the reconstruction behaviour for a representative subject (Subject 1) to demonstrate the model’s ability to capture resting-state dynamics and quantify reconstruction fidelity before deviation detection.

To mitigate cognitive overload, only the first 60 s of the mental arithmetic task were recorded (see Section 2.1). Consequently, no task-related EEG segments extend beyond this duration. This limitation is illustrated in Supplementary S2 Fig.

Fig 2 illustrates the reconstruction performance of 1-second EEG segments during the mental arithmetic task and the resting state.

**Fig 2:**
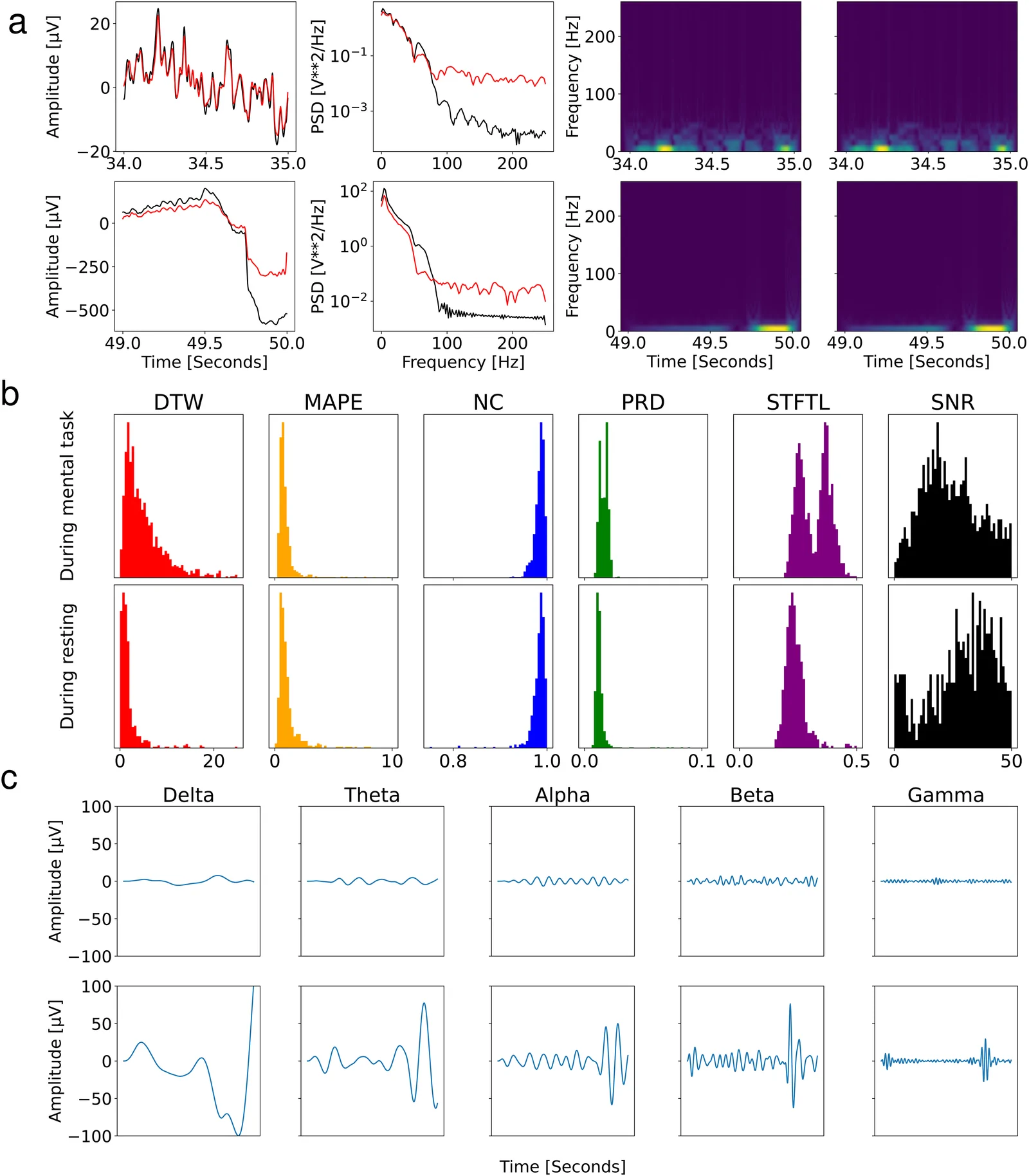
1-second segments reconstruction during mental counting and resting. **a.** Each row corresponds to a different EEG segment during the mental task from Subject 1. The four columns represent, respectively, the time-domain signal, its frequency-domain representation, the spectrogram of the original signal, and the spectrogram of the reconstructed signal. The top row shows the segment with the lowest reconstruction error, while the bottom row shows the segment with the highest reconstruction error. Original and reconstructed signals are shown in black and red, respectively. **b.** Distributions of the error metrics (see Section 2.2.3) computed for EEG segments during the mental arithmetic task (top row) and the resting state (bottom row). **c.** Signals decomposed into canonical EEG frequency bands (Delta–Gamma) for the best (top) and worst (bottom) reconstructed segments. The worst reconstruction shows severe degradation in the higher-frequency components, reflected by the exceptionally large DTW values (> 10,000).

In Fig 2a, we show the segments with the lowest and highest DTW values during the arithmetic task (top and bottom rows, respectively). The top row corresponds to the best reconstruction (minimal DTW), whereas the bottom row corresponds toshows the best reconstruction (minimal DTW), whereas the bottom row shows the worst reconstruction (maximal DTW; see Section 2.2.3). A clear deviation between the original and reconstructed signals is observed in the worst case, particularly toward the end of the segment (49–50 s), where the DTW reaches approximately 13,000 s.

Fig 2b shows the distributions of the reconstruction metrics (DTW, NC, MAPE, PRD, STFTL, and SNR) for Subject 1 during the mental arithmetic task (top row) and the resting state (bottom row).

Compared with the resting condition, the mental task showed a broader distribution of DTW values, indicating greater variability in reconstruction quality. In addition, the PRD and STFTL distributions showed bimodal patterns during the mental task. These two modes likely correspond to well-reconstructed segments and segments with markedly degraded reconstruction (outliers).

Supplementary S3 Fig presents the distributions of these metrics across all subjects, with statistical significance assessed using the Mann–Whitney U test (see Section 2.2.9).

Table 2 presents a statistical summary of the reconstruction error metrics across segments from all subjects, corroborating the variability patterns previously observed in Fig 2b. Outliers were evident, as reflected by maximum values that markedly deviated from the corresponding medians, particularly for DTW, MAPE, PRD, and STFTL. Notably, reconstruction errors (except NC) were significantly higher during the arithmetic task than in the resting state (*p* < 0.001), in agreement with the broader, bimodal distributions reported earlier.

**Table 2:** Descriptive statistics summary of errors.

	Resting state						Arithmetic task					
	DTW	NC	MAPE	PRD	STFTL	SNR	DTW	NC	MAPE	PRD	STFTL	SNR
**Mean**	1.55	0.98	2.02	0.01	0.23	28.06	5.67	0.96	420.63	0.07	0.66	34.24
**STD**	69.83	0.02	18.91	0.01	0.08	16.74	49.81	0.11	11829.01	0.92	8.06	19.45
**Min**	−0.95	0.65	0.06	0.00	0.06	0.02	−1.02	−0.24	0.07	0.00	0.05	0.04
**Q25**	−0.18	0.98	0.61	0.01	0.16	21.23	0.01	0.98	0.66	0.01	0.18	15.69
**Median**	0.46	0.99	0.93	0.01	0.22	24.83	0.18	0.98	0.89	0.01	0.19	31.82
**Max**	13819.93	1.00	1864.34	0.05	0.65	182.73	2442.09	1.00	1259522.71	62.22	636.71	313.42
**Q75**	0.86	0.9	1.42	0.01	0.23	44.50	1.2	0.99	1.41	0.02	0.27	37.07
**IQR**	1.04	0.01	0.81	0.00	0.08	23.27	1.28	0.01	0.75	0.01	0.09	21.38

For intra-subject comparisons, 72%, 63%, and 63% of subjects showed statistically significant differences between rest and task conditions in DTW, STFTL, and SNR, respectively (*p* < 0.05, *p* < 0.01, and *p* < 0.05 on average). Conversely, the distinctions observed in MAPE and NC metrics were less consistent across subjects (44% and 0%, respectively), despite global statistical significance (*p* < 0.001 on average). These discrepancies in error metrics are more pronounced during the arithmetic task than in the resting state, suggesting increased reconstruction instability under cognitive load.

Furthermore, reconstruction errors were detectable across a broader range of evaluation metrics than those used during training (see Section 2.2.2), indicating that post-hoc multi-metric assessment provided a more comprehensive characterisation of reconstruction quality.

Regarding the frequency content of the segments, the reconstructed signal inadequately recovers frequency components above ~100 Hz, which lie well beyond the physiologically relevant EEG bands spanning 0–40 Hz. This discrepancy is evident in the power spectral density shown in the middle panels of Fig 2a, where deviations between original and reconstructed spectra become pronounced at higher frequencies.

Additionally, the standard deviation of the signals was examined across the canonical frequency bands—delta (1–4 Hz), theta (4–8 Hz), alpha (8–13 Hz), beta (13–30 Hz), and gamma (30–45 Hz)—as presented in Fig 2c. The standard deviation of the original rhythms in segments with the poorest reconstruction was consistently higher than in segments with the best reconstruction across all subjects, although the magnitude of this difference varied across bands (*p* < 0.001). In the delta band, the standard deviation increased on average from 3.08 μV to 13.69 μV (a 4.44-fold increase), whereas in the gamma band it rose from 0.87 μV to 2.84 μV (a 3.26-fold increase).

For spectral power, we quantified the area under the Power Spectral Density (PSD), expressed in ${\mu V}^{2}$. This analysis revealed substantial differences between segments with optimal and poor reconstruction in the delta, theta, and beta frequency bands. The mean PSD areas in the optimally reconstructed segments were 12.90, 0.80, and 2.16 ${\mu V}^{2}$, respectively, whereas the corresponding values in the poorly reconstructed segments increased to 34.28, 35.48, and 80.76 ${\mu V}^{2}$ (*p* < 0.001 in all cases). These findings indicate significantly elevated spectral power in the poorly reconstructed segments within these frequency bands. In contrast, we observed no statistically significant differences in the alpha and gamma bands.

Given the observed discrepancies in both the temporal and spectral domains, we performed a time–frequency analysis using spectrograms of the original and reconstructed signals (*time vs frequency*). In the segment reconstructed with the highest accuracy, we observed distinctive electrophysiological activity across frequency bands extending from delta to gamma (< 40 Hz). In contrast, such broadband activity was absent in the segment with the poorest reconstruction. In this latter case, electrophysiological activity was largely confined to the 0–13 Hz range, encompassing the delta, theta, and alpha bands.

S4 Fig in supplementary material illustrates the temporal statistical differences between the resting and counting states for both original and reconstructed signals. The reconstructed signals show no notable differences between normal and outlier segments when assessed using conventional temporal statistics.

In summary, segments associated with the task stage show higher reconstruction errors than those from the resting stage, consistent with previously reported variability in error metrics. Moreover, traditional statistical descriptors derived from EEG data may be insufficient to discriminate between normal and abnormal data within the dataset. Notably, poorly reconstructed segments exhibit elevated spectral power in the delta, theta, and beta bands, whereas the most accurately reconstructed segments preserve rhythmic content extending up to 40 Hz. Collectively, the Mann–Whitney U tests demonstrated that reconstruction errors were significantly elevated during the arithmetic task relative to the resting state for five of the six metrics (*p* < 0.001, FDR-corrected), providing strong statistical evidence that the proposed multi-metric feature space is able to discriminate between resting-state activity and task-induced brain activation. At the single-subject level, the OCSVM classifier then identifies which segments deviate beyond the resting-state distribution, offering temporal and channel-level resolution that global statistics alone cannot provide; thus, the proposed framework jointly fulfils the requirements of condition discrimination and abnormal-segment identification.

### 3.2. Validation of statistical anomaly detection

After comparing the original and reconstructed signals using different error metrics, we validated the anomaly detection method with synthetic data. Because we used generative artificial intelligence with a variational auto-encoder (see Section 2.2.2), we could synthesise signals using the trained models. Because the models were trained on resting-state segments, they could be used to generate resting signals that were later modified to mimic abnormal segments. Section 2.2.8 details the procedure for generating these synthetic abnormalities. We then used these synthesised segments to validate the proposed methodology with three anomaly detection algorithms: OCSVM, Isolation Forest (IF), and Local Outlier Factor (LOF) (see Section 2.2.5 for details).

Table 3 summarises the performance of the evaluated algorithms. In the proposed framework, we trained the models on reconstruction errors from real resting-state data and then assessed them on synthetic data. For normal samples, OCSVM achieved the highest accuracy, whereas IF and LOF performed worse and often misclassified normal data. Conversely, for abnormal samples, both IF and LOF achieved near-perfect accuracy and surpassed OCSVM. In summary, among the three methodologies evaluated, OCSVM offered the most balanced trade-off between normal-sample retention and abnormal-sample detection in this configuration. This synthetic validation only verifies that the detectors reject samples outside the resting-state support (the abnormal-detection rates), an outcome insensitive to feature scaling (rejection ≥90% with and without a scaler). We therefore applied normalisation only to the real-EEG pipeline, where deviations are subtle and scale-dependent, and did not use it in this scale-insensitive sanity check.

**Table 3:** Performance of classification methods using the subject’s resting data.

Accuracy	OCSVM	IF	LOF
Normal samples	64%±38%	8%±24%	2%±13%
Abnormal samples	90%±10%	99%±1%	99%±2%

Consider the scenario in which a model is trained using the resting data from subjects other than the one being analysed. Table 4 summarises the performance metrics for these algorithms when each subject trains on the resting data of all other subjects, excluding their own. Notably, overall performance varies little, with only a marginal reduction in normal-sample detection. Because training with additional resting data can be time-intensive and memory-demanding, it is best to focus on a configuration that uses only the subject’s resting data under analysis.

**Table 4:** Performance of classification methods using the resting data of others from all other subjects.

Accuracy	OCSVM	IF	LOF
Normal samples	63%±10%	13%±8%	4%±1%
Abnormal samples	90%±2%	99%±1%	99%±1%

### 3.3. Detection of mental task-induced deviations

After validating outlier detection, the next phase involved identifying and characterising segments that showed substantial deviations from the resting-state baseline during the mental arithmetic task. In this context, segments classified as outliers reflect statistical deviations from the resting reference distribution rather than clinical abnormalities.

As shown in Table 2, DTW and MAPE are strongly influenced by extreme values: although maximum DTW and MAPE values exceed 100, their medians remain below 10, suggesting that only a subset of segments shows pronounced reconstruction deviations relative to resting errors.

Given each segment’s high-dimensional representation (six reconstruction-error features), we analysed both task-state outliers and resting-state segments using tridimensional Uniform Manifold Approximation and Projection (UMAP) [73]. This dimensionality reduction technique enables visualisation of six-dimensional feature vectors in a three-dimensional space [74], as illustrated in Fig 3.

**Fig 3:**
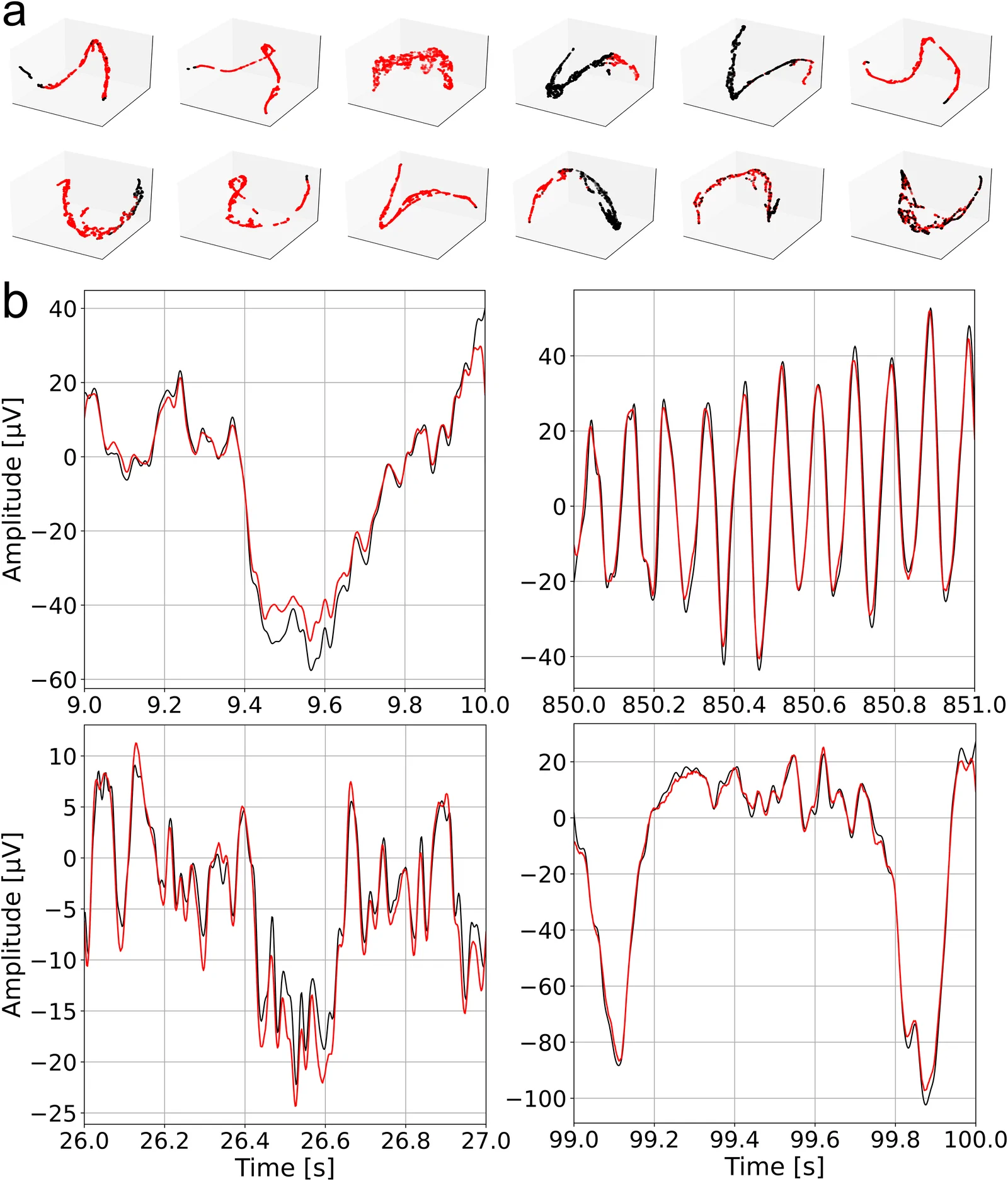
Visualization of statistically deviant segments using three-dimensional UMAP analysis. **a.** Each scatter plot shows the three-dimensional UMAP embedding (fitted on resting-state segments) of the 6-dimensional reconstruction-error feature vectors corresponding to 1-second segments from the first eight subjects. Task-state segments classified as baseline and statistically deviant are represented by red and black dots, respectively. **b.** Four randomly selected statistically deviant EEG segments. The original and reconstructed signals are shown in black and red, respectively.

Fig 3a reveals a non-linear separation between statistically deviant (black) and baseline (red) segments. Although the two groups remain relatively close in the embedded space, a discernible separation is observed.

Across all subjects, between 0.8% and 99.9% (Mean = 28%, SD = 28%) of the task segments were identified as deviant. This variability indicates that not all task segments differ substantially from resting-state behaviour. Consequently, a subset of subjects exhibits limited differentiation between task and resting conditions, as shown in Fig 3b. To confirm that the observed task deviant rate reflects genuine task-related deviations rather than the detector’s intrinsic false-alarm rate, we calibrated it against a held-out resting-state baseline. For each subject, resting segments were held out from the detector fit using 5-fold cross-validation and scored identically to the task segments, so that the resting baseline was never used to classify itself. The held-out resting deviant rate was low and tightly concentrated around the detector’s nominal false-alarm level (mean = 10.3%, SD = 0.5 percentage points), and significantly below the task deviant rate (mean = 28%; one-sided Wilcoxon signed-rank test, *p* = 0.001; the task rate exceeded the resting rate in 23 of 36 subjects). This indicates that task segments deviate from the resting-state distribution beyond what the detector’s baseline false-alarm rate would expect. To investigate whether subjects with high deviant rates (i.e., the percentage of task-related segments classified as deviants) correspond to those with poor reconstruction quality, we computed the Spearman correlation between the per-subject deviant rate and the mean value of each reconstruction metric computed over the normal segments. No significant correlation was found for DTW (ρ=−0.25, *p* = 0.15), NC (ρ=−0.04, *p* = 0.80), MAPE (ρ=+0.01, *p* = 0.95), PRD (ρ=+0.31, *p* = 0.07), or SNR (ρ=+0.01, *p* = 0.99). Similarly, the correlation between the deviant rate and the number of resting-state segments used for training was negligible (ρ=−0.19, *p* = 0.27), indicating that training-set size does not account for this variability. The only statistically significant association was observed for STFTL (ρ=+0.55, *p* < 0.001), indicating that subjects with higher deviant rates tend to exhibit higher average short-time Fourier transform (STFTL) reconstruction error even on their normal segments. Because no such association was found for five of the six error metrics or for the training-set size, the inter-subject variability in deviant rates is largely independent of baseline reconstruction quality and data availability, and therefore primarily reflects genuine inter-subject differences in EEG responses to mental arithmetic; the moderate STFTL association nonetheless indicates that a spectral component of reconstruction quality also contributes to this variability.

Segments identified as statistically deviant are expected to exhibit elevated reconstruction errors compared to baseline segments. Statistical analyses confirmed that 56%, 61%, 19%, and 53% of deviant segments showed significantly greater errors than baseline segments for DTW, NC, STFTL, and SNR metrics, respectively (all *p* < 0.01 on average, FDR-corrected). No significant differences were found between deviant and baseline segments for the MAPE metric.

With respect to frequency content, significant differences in signal standard deviation between deviant and baseline segments were observed in 83% (*p* < 0.01), 78% (*p* < 0.01), 72% (*p* < 0.05), 83% (*p* < 0.01), and 78% (*p* < 0.01) of subjects across the delta, theta, alpha, beta, and gamma bands, respectively. These findings indicate that statistical deviations affect signal variability broadly across all frequency bands (72–83% of subjects), with the delta and beta bands showing the highest proportions (both 83%).

Furthermore, differences in PSD area between deviant and baseline segments were identified in 47% (*p* < 0.01), 42% (*p* < 0.01), 36% (*p* < 0.05), 53% (*p* < 0.01), and 36% (*p* < 0.01) of subjects across the delta, theta, alpha, beta, and gamma bands, respectively. This indicates that frequency-density alterations are more consistently observed in the delta and beta bands.

Overall, statistical deviations manifest as increased signal variability broadly distributed across the spectrum, whereas PSD-based differences are less uniform and most consistent in the delta and beta bands. Since PSD lacks temporal resolution and cannot determine when specific frequency components emerge or disappear within a 1-second segment, examining temporal rhythm dynamics may provide deeper insight. Such temporal analysis may reveal that statistical deviations are characterised not solely by spectral shifts, but also by transient fluctuations.

To assess whether the detected (observed) deviations could be attributed to artefacts, we examined each deviant segment using two commonly used heuristic indices: peak-to-peak (PTP) amplitude and signal kurtosis. Segments were classified as artifacts when PTP>100μV or kurtosis > 5. On average, only 7.4% (SD = 11.1 percentage points) of the deviant segments satisfied this criterion. This relatively small proportion suggests that the identified deviations are largely not attributable to high-amplitude or impulsive artefacts and are more consistent with task-related neural dynamics. Although significant differences were also present in the beta and gamma ranges, where myogenic activity is most prominent, the comparable or stronger involvement of the delta band, which is not associated with muscular artefacts, argues against a purely myogenic origin. Nonetheless, we cannot definitively rule out a residual myogenic contribution to the higher-frequency bands, given the dataset’s lack of dedicated EOG/EMG reference channels.

### 3.4. Channel-level analysis of statistical deviations

After identifying statistically deviant segments during the mental arithmetic task, we conducted a channel-level analysis to determine whether these deviations exhibit spatial structure across the scalp. While previous sections characterised deviations in the reconstruction-error feature space and frequency domain, the present analysis aims to localise their distribution across EEG electrodes.

Fig 4 presents the spatial distribution of statistically deviant segments across EEG channels. Segments classified as statistically deviant during the mental arithmetic task were compared with baseline segments to evaluate electrode-specific contributions. As shown in Fig 4a, lateral and peripheral electrodes—particularly those positioned outside the central scalp region—account for a substantial proportion of statistically deviant segments. Within the cohort categorised as good-quality counters, the electrodes most frequently associated with statistically deviant segments were Fp2, O2, and T6 (followed closely by O1 and T3), each exhibiting approximately 25–30% of segments classified as deviant. The Fp1 electrode, adjacent to Fp2 and associated with the prefrontal cortical region, is implicated in executive and mental processing functions. Similarly, the T5 electrode, adjacent to T6 and anatomically related to temporal lobe structures including the fusiform gyrus (near Brodmann area BA 37), is linked to higher-order language and visual processing.

**Fig 4:**
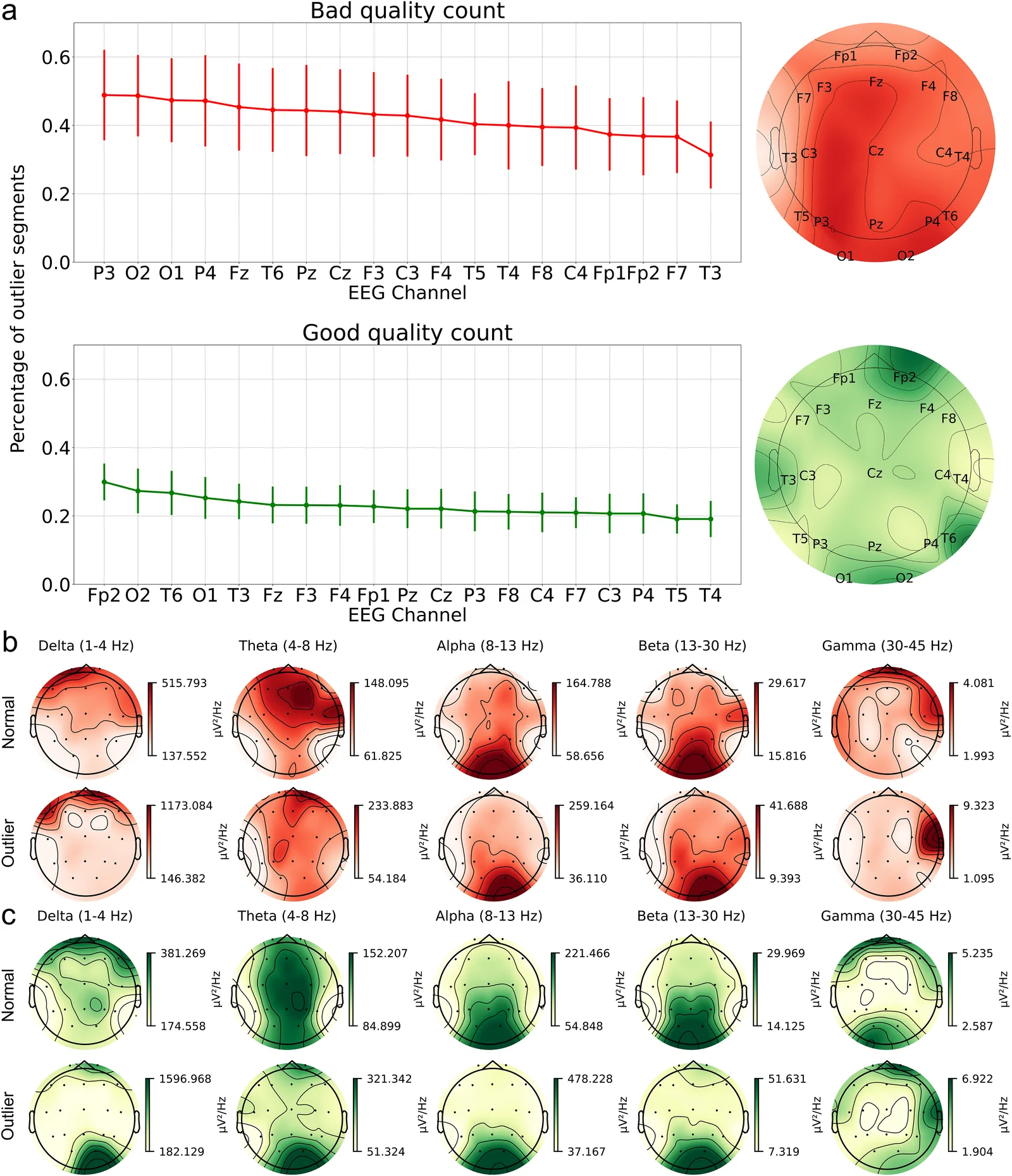
Channel-level distribution of task-state statistical deviations. **a.** Spatial distribution of statistically deviant segments across scalp electrodes for lower-performing (top) and higher-performing (bottom) counters during the arithmetic task. Warmer colours indicate a higher percentage of statistically deviant segments per electrode. **b.** Power spectral density (PSD) comparison between baseline and statistically deviant segments across frequency bands for lower-performing counters. **c.** Same analysis as in **b** for higher-performing counters.

In the cohort categorised as poor-quality counters, the electrodes exhibiting the highest proportion of statistically deviant segments were P3, O2, and O1 (with P4 and Fz close behind), with approximately 45–49% of segments classified as deviant. Moreover, poor-quality counters showed a higher proportion of deviant segments across nearly all electrodes (≈40–49%) than good-quality counters (≈19–30%), indicating a global rather than local increase. Regardless of counting proficiency, statistically deviant segments predominantly involved peripheral electrodes, although differences across electrodes were modest. Notably, the T3 electrode ranked among the most deviant electrodes in good-quality counters but was the least deviant electrode in poor-quality counters, suggesting potential differences in task engagement or cortical recruitment patterns.

We further examined the temporal continuity of statistically deviant segments by quantifying consecutive occurrences within each electrode. On average, electrodes exhibited 6.87 (SD = 15.16), 7.70 (SD = 16.61), and 8.51 (SD = 17.42) consecutive deviant segments, respectively. These results indicate that deviations tend to occur in short bursts rather than prolonged intervals, reflecting temporal specificity in the proposed detection framework.

The observed spatial heterogeneity across electrodes is consistent with the spectral differences previously reported between baseline and statistically deviant segments. As shown in Fig 4b and 4c, baseline segments demonstrate stronger central frequency activity (a pattern also observed in the resting state; S6 Fig in the supplementary materials), whereas statistically deviant segments exhibit reduced central spectral power and comparatively greater activity in peripheral regions.

Overall, these findings suggest that task-state statistical deviations are not uniformly distributed across the scalp, but rather preferentially involve peripheral electrodes, accompanied by altered spectral organisation relative to baseline segments.

### 3.5. Co-occurrence analysis of task-state deviations

So far, we characterised task-induced deviations first at the segment level—through reconstruction errors and spectral differences—and then at the single-channel level—through the spatial distribution of deviant segments across the scalp. Here, we extended the analysis to the inter-channel level by examining whether, and in what combinations, the detected deviations co-occur across electrode pairs and triplets. Together, these three levels of analysis—segment, channel, and network—provide a complementary characterisation of cognitive load effects during mental arithmetic.

Building on the spatial characterisation of statistically deviant segments across individual electrodes, the next step examined whether these deviations co-occur across multiple channels. While previous analyses focused on segment-level deviations and their spatial distribution, the present section investigates inter-electrode relationships to determine whether statistically deviant activity manifests as coordinated multi-channel patterns.

Supplementary S5 Fig presents representative examples of segments identified as statistically deviant across four channels. On average, approximately five electrodes (SD = 6) exhibited statistically deviant segments concurrently across all subjects, with poor-quality counters showing more concurrent deviant channels (≈8) than good-quality counters (≈4). However, this aggregate number does not indicate which specific electrode combinations are most prone to co-occur during such deviations.

Fig 5 illustrates the time-frequency co-occurrence analysis across multiple EEG channels (see Section 2.2.6). In Fig 5a, the most frequent co-active electrode combinations are identified. Among triplets, the combinations (T4,T6,O2), (C3,P3,P4), (P3,O1,O2), (F4,O1,O2), and (O1,O2,Fz) were the most common. The most frequent electrode pairs include (P3,O2) and (P3,O1), followed by (P4,Cz), (P3,P4), and (F3,P3). These patterns suggest that specific electrode groups—particularly involving parietal, occipital, central, and temporal regions tend to exhibit statistically deviant activity simultaneously during the arithmetic task.

**Fig 5:**
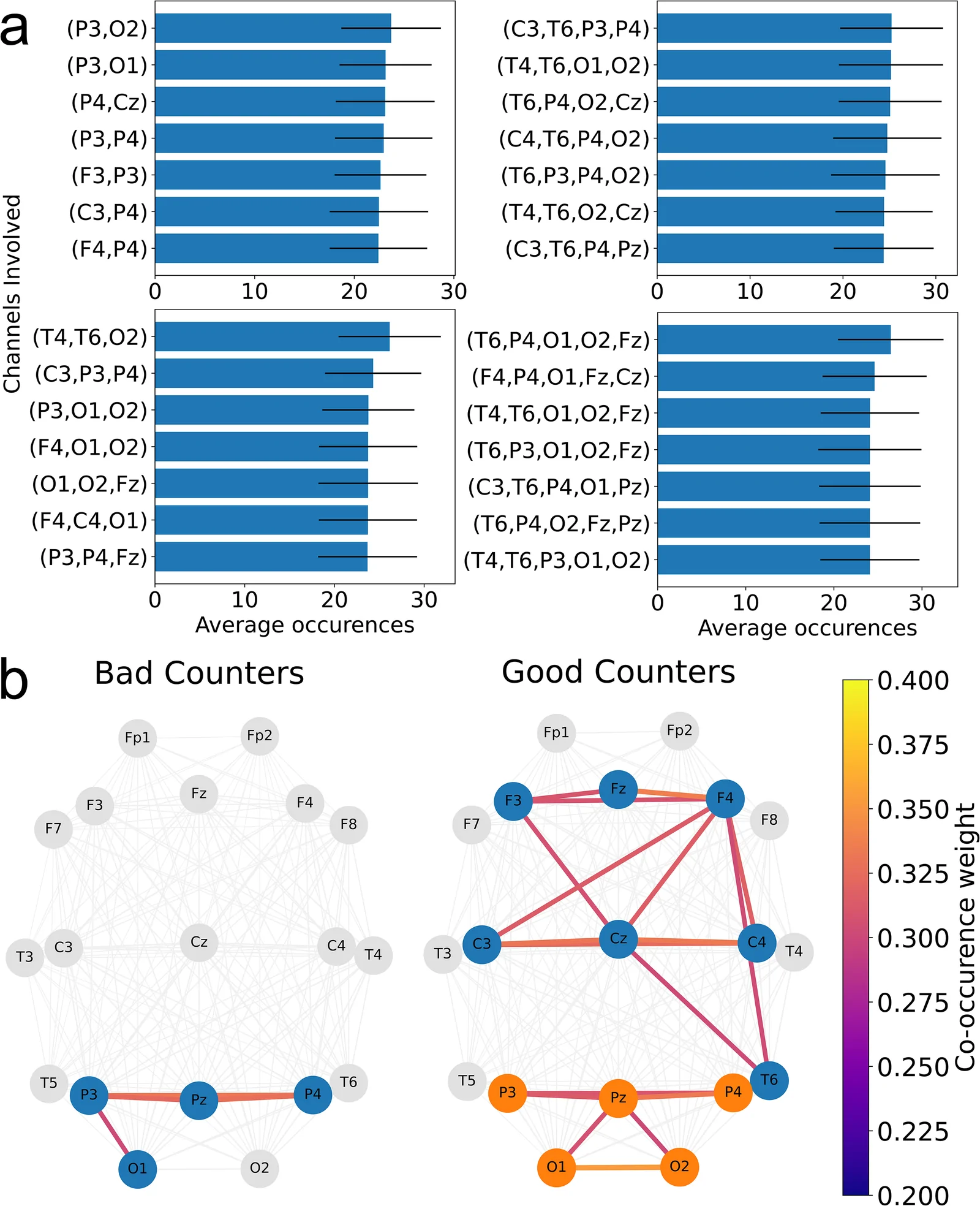
Time–frequency co-occurrence analysis of statistically deviant segments across EEG channels. **a.** Bar plots showing the average co-occurrence frequency of statistically deviant EEG segments across electrode combinations of size two to five (from left to right) during the arithmetic task across all subjects. **b.** Graph-based representation of average co-occurrence patterns for lower- and higher-performing counters. Nodes belonging to the same community are displayed in the same colour (grey nodes have no assigned community). Edge thickness and colour encode the Jaccard co-occurrence weight: faint grey edges denote sub-threshold co-occurrences (≤0.3), whereas thick, colour-coded edges denote the strong co-occurrences (> 0.3) on which community detection was performed.

To further examine inter-electrode interactions, we conducted a graph-theoretical analysis (see Section 2.2.6). In this representation, we treated electrodes as nodes and weighted edges by co-occurrence frequency. As shown in Fig 5b, subjects with lower performance displayed a sparse co-occurrence graph (modularity Q≈0), whereas higher-performing subjects exhibited denser and more structured co-occurrence patterns (modularity *Q* = 0.45). After removing edges with co-occurrence weights below 0.3, greedy modularity community detection identified two communities among the higher-performing subjects, while lower-performing subjects formed a single parieto-occipital community (P3, P4, Pz, and O1). The principal community consisted of electrodes P3, Pz, P4, O1, and O2, while a secondary fronto-central cluster included F3, F4, C3, C4, Cz, Fz, and T6.

Consistent with the channel-level analysis, statistically deviant segments were predominantly observed at peripheral electrodes, particularly over the prefrontal (Fp2), temporal (T6), and posterior occipital and parietal regions. Spectral analyses indicated that deviations were most pronounced in the delta (1–4 Hz) and theta (4–8 Hz) bands. Using normalised correlation, simultaneously deviant segments exhibited an average correlation comparable to baseline and resting simultaneous segments (0.52±0.14 vs 0.50±0.08; *p* = 0.36, not significant). No significant differences were observed in coherence measures between deviant and baseline segments.

Overall, segment-level reconstruction deviations were not uniformly distributed over time, while their inter-channel synchrony did not differ from baseline. Instead, we observed transient activations. These findings suggest that cognitive load during arithmetic processing may involve dynamic and spatially distributed neural activity. More broadly, they highlight the utility of segment-based, single-channel, statistical deviation detection in capturing transient, spatially heterogeneous task-related dynamics.

## 4. Conclusion

This study introduced a segment-level anomaly detection framework that integrates a hvEEGNet, trained exclusively on resting-state EEG, with an OCSVM to detect task-induced statistical deviations during mental arithmetic, based solely on reconstruction errors. By analysing short, 1-second EEG segments, the proposed method enables fine-grained identification of transient deviations from the resting-state baseline. hvEEGNet learns to reconstruct baseline brain signals, and reconstruction errors computed on task-related data serve as the basis for statistical deviation detection.

The results demonstrate that task-state deviations are not uniformly distributed but exhibit spatial and spectral structure. These statistically deviant segments were predominantly localised over the prefrontal and temporal regions and were primarily associated with alterations in the delta and theta bands, reflecting transient activations. Channel-level and co-occurrence analyses further revealed that such deviations tend to occur in spatially distributed yet temporally specific patterns rather than as globally synchronised events.

The proposed methodology leverages the generative capacity of VAEs and the one-class classification paradigm, enabling modelling of complex data distributions without labelled data. Nonetheless, VAEs remain computationally demanding and sensitive to training configuration, while OCSVM-based detection trades off sensitivity to statistically deviant segments against retention of baseline segments.

Overall, this self-supervised framework provides a label-free solution for identifying transient task-induced deviations in EEG signals and offers a multi-scale characterisation of cognitive load effects.

Taken collectively, these findings may contribute to resolving the inconsistencies reported across previous studies analysing the same dataset [14–17]: because task-induced deviations are transient and spatially heterogeneous, analyses that treat the EEG recording as a whole are likely to capture different temporal windows or channel subsets of this underlying variability, which may explain the inconsistent discriminative electrodes and classification performances previously reported. Neurophysiologically, the predominance of statistically deviant segments over prefrontal (Fp2) and temporal (T6) electrodes, together with the associated delta/theta band changes, is consistent with executive-control and working-memory-related cortical regions during mental arithmetic, and suggests that this label-free, subject-specific framework could support fine-grained monitoring of cognitive load in applications ranging from adaptive learning environments to clinical settings where deviations from an individual’s own baseline are of interest. This is without even considering the temporal resolution achieved in the analysis.

Perspectives include extending the framework from offline analysis to real-time cognitive-state monitoring to detect transient cognitive overload. Future research may also examine adaptive and transfer-learning strategies to balance subject-specific sensitivity with cross-subject generalisation. A comprehensive, synthetically generated validation study that incorporates a broader spectrum of perturbation morphologies, extending beyond purely sinusoidal components to include, for example, transient burst-like events and non-stationary coloured noise, would further enhance confidence in both the robustness and the generalizability of the proposed detection framework. Another limitation concerns the lack of a systematic hyperparameter optimisation strategy. Future work will address this issue, for example, through cross-validation restricted to resting-state data. Additionally, extending the current co-occurrence framework toward time-resolved functional network modelling may provide deeper insight into the dynamical organisation of task-induced deviations. Finally, this methodology can be applied to other modalities in which no mental tasks are performed and where mental diseases such as Alzheimer are studied, provided a predefined normal dataset is available.

## Supporting information

S1 Fig Electrodes used in this study. This illustration shows the locations of the electrodes used in the present study.
(EPS)

S2 Fig Example of last EEG segments per channel during the mental arithmetic task. Last segments from channels Fp1, Fp2, F3, F4, and F7. Segments over 60 s were removed as they showed no relevant activity in conformance with the database specification.
(EPS)

S3 Fig Distribution of errors across all subjects. Distributions of the error metrics DTW, NC, MAPE, PRD, and STFTL during mental task (top row) and resting state (bottom row).
(EPS)

S4 Fig Statistical metrics from the original and reconstructed segments in the temporal domain. Visual inspection shows that these metrics do not report statistical discrimination between the original (blue) and reconstructed (red) signals in both resting and counting states.
(EPS)

S5 Fig Signals from Fp1, Fp2, T5, and T3. Signals with segments classified as normal and abnormal segments. Those segments coloured in red are classified as abnormal.
(EPS)

S6 Fig Spectral analysis of scalp electrodes during the resting state. Bad counters (red) and good counters (green).
(EPS)

S7 FigPairwise Pearson correlation matrix of the six reconstruction-error metrics.Correlation matrix computed on segments recorded during the mental arithmetic task (DTW, NC, MAPE, PRD, STFTL, SNR). The only pair showing high correlation (ρ=0.87) is PRD and STFTL; all remaining pairs satisfy |ρ|<0.80.(EPS)
