# Supplementary material for: A self-supervised reconstruction framework for segment-level detection of task-induced deviations in multi-channel EEG
Source: PLoS One. 2026 Oct 30;21(10):e0361586. doi: 10.1371/journal.pone.0361586 (PMC13722333; doi:10.1371/journal.pone.0361586)
Supplement: Supplementary file 1 [file pone.0361586.s008.pdf]

Recommendation: Major Revision

Summary: This study presents a 1-second segment-based, self-supervised framework that utilizes a hierarchical variational autoencoder (hvEEGNet) and a One-Class SVM (OCSVM) to detect EEG signal deviations (anomalies) during mental arithmetic tasks. The study's aim to capture transient cortical dynamics by examining signals in short-term segments rather than analyzing them globally is scientifically valuable. However, there are very serious structural issues regarding methodological originality, the clear contribution to literature, the validity of the validation using synthetic data, and the reliability of the physiological interpretations that must absolutely be addressed before acceptance for publication.

Major Comments:

- **Weak Contribution to the Literature and Lack of Methodological Originality:** The authors use the "hvEEGNet" architecture for signal reconstruction, and the text explicitly states that this architecture was already developed by Cisotto et al. (Ref [24]). The primary methodological contribution of this study appears to consist merely of adding metrics such as DTW, MAPE, PRD, STFTL, and SNR to the outputs of an existing model to perform anomaly detection with an OCSVM algorithm. The authors must go beyond simply testing an existing model on a new dataset; they must provide strong theoretical proof of why their methodology is structurally superior or more innovative than previous approaches (e.g., those using only kNN or simple thresholding).
- **Circularity in Validation with Synthetic Data:** In Section 2.2.8, the authors state that they generated synthetic data using the already trained hvEEGNet model to validate the anomaly detection algorithms (OCSVM, IF, LOF). Validating an anomaly detection algorithm tested on synthetic data generated by a VAE (hvEEGNet) itself, using its own reconstruction errors, is methodologically flawed (a circular logic error). The VAE will transfer its own learned biases to the synthetic data. This does not scientifically prove that the model can capture real-world physiological anomalies; it merely shows that the model validates itself.
- **Physiological Interpretations and the Possibility of Artifacts (Noise):** The study finds that statistical deviations occur more frequently in peripheral electrodes such as Fp1, Fp2, T3, and T5. It is well known in the literature that Fp1 and Fp2 are highly susceptible to blinking/eye movements (EOG), while T3 and T5 are highly susceptible to jaw or neck muscle tension (EMG). Although the authors claim the dataset used is "artifact-free," involuntary muscle contractions (e.g., frowning) can occur during tasks requiring high cognitive effort, such as mental arithmetic. The authors must discuss in detail whether these detected deviations are truly complex cognitive processes or physiological artifacts (muscle tension) caused by increased mental effort.
- **Correlation of Metrics and Dimensionality (Curse of Dimensionality):** A vector of 6 different metrics (DTW, NC, MAPE, PRD, STFTL, SNR) is provided as input to the OCSVM for anomaly detection. Error metrics such as MAPE and PRD generally show

a very high correlation with each other. This situation can lead to unnecessary dimensionality in the model. The authors should present the correlation matrix between these 6 features and increase the robustness of the model by eliminating redundant features.

Section-by-Section Comments:

- **Introduction:** The introduction states that previous studies found contradictory results on the same dataset. However, it is not fully justified why the proposed "1-second segment" approach will physiologically resolve these contradictions in the literature. Window size is very critical in signal processing; why were exactly 1-second windows chosen instead of 2 seconds or 0.5 seconds? The signal processing or physiological foundations of this choice must be explained.
- **Methods:** How the hyperparameters of the One-Class Learning algorithms (OCSVM, IF, LOF) (e.g., kernel type or *nu* parameter for OCSVM, number of trees for IF) were selected and optimized is left missing in the methodology. The hyperparameter optimization process must be detailed.
- **Results:** In Section 3.4, the spatial differences (electrode-based) between "good quality counters" and "poor quality counters" are discussed. However, it is not specified in the Methods section exactly what criteria were used to define these subject groups in the dataset, how they were separated, and how many patients are in each group.
- **Conclusion:** The conclusion section should concretely state how the findings align with (or resolve) previous contradictory findings in the literature. Furthermore, the inferences regarding the practical clinical or neurological benefits of the article remain weak and should be strengthened.
