# Supplementary material for: A self-supervised reconstruction framework for segment-level detection of task-induced deviations in multi-channel EEG
Source: PLoS One. 2026 Oct 30;21(10):e0361586. doi: 10.1371/journal.pone.0361586 (PMC13722333; doi:10.1371/journal.pone.0361586)
Supplement: Supplementary file 2 [file pone.0361586.s009.pdf]

Reviewer comments

PONE-D-26-16141, “A self-supervised reconstruction framework for segment-level detection of task-induced deviations in multi-channel EEG” Abstract:

1. *Please declare the electroencephalogram first before using the ”EEG” abbreviation in the second line.*
2. *Please remove the abbreviation ”hvEEGNet” because it is not used in the abstract.*
3. *Please remove the abbreviation ”DTW, STFT, SNR” because they are not used in the abstract.*
4. *Please review the tense used in describing the experimental methods and results, it is preferred to use past tense.*

Introduction section:

1. *Please use (i.e. BA 46) instead of (BA 46). (line 17)*
2. *Please reorder the cited references to be [3] [10] instead of [10] [3]. (line 22)*
3. *Please declare ”SVM” first before using the abbreviation. (line 40)*
4. *Please declare both ”LDA and kNN” before using them. (line 51)*
5. *In line 74, what do you mean by ”...,which are not available for this dataset”, which dataset are you referring to?*
6. *Please declare ”hvEEGNet” first before using it. (line 88)*
7. *Please declare all ”NC, PRD, STFTL, and MAPE” first before using them. (line 94)*
8. *Please no need to use slanted font style in the paragraph started from line 108 to line 114.*
9. *Please remove the ”,” between segment-level and self supervised framework. (line 115)*
10. *In the last paragraph, all subsections of section 2 were mentioned except subsections 2.2.7 -2.2.9, please state them.*

Material and Methods section:

1. *In section 2.1, please mention brief demographic information about the dataset used.*
2. *In section 2.2, have you calculated the training time for the proposed framework? if yes, please state the computation capabilities used and the average time consumed.*
3. *(line 176) please use (i.e. electrode) instead of (electrode).*
4. *(line 181) please use (i.e. each value....) instead of (each value....). Do the same throughout the paper.*
5. *Please remove the last sentence in the caption of Figure 1, as it already explained in the context.*

6. *(line 211) please use (Eq. 1) instead of (1) to refer to Equation 1.*
7. *In subsection 2.2.2, please enumerate all the equations before Equation 4.*
8. *(line 437 and 439) please use ($N = 200$) instead of ($\mathrm{N} = 200$).*
9. *In the statistical analysis subsection, Did you test the normality of data using Shapiro-Wilk test first before using the Mann-Whitney U test? if, yes please state the test p-value.*

Results and Discussion section:

1. *In this section, it is recommended to use the past tense in reporting the findings.*
2. *(line 525) Please use "60 s" instead of "60 seconds". Please use "s" to refer to seconds throughout the paper.*
3. *(line 535) What is the unit of measurement of DTW that reached approximately 13000.*
4. *(line 578) What is the unit of measurement of the standard deviation that increased frome 3.08 to 13.69 and similarly for the gamma band.*
5. *I can't find figure 4 and 5 in the submitted file.*
