# Supplementary material for: A self-supervised reconstruction framework for segment-level detection of task-induced deviations in multi-channel EEG
Source: PLoS One. 2026 Oct 30;21(10):e0361586. doi: 10.1371/journal.pone.0361586 (PMC13722333; doi:10.1371/journal.pone.0361586)
Supplement: Supplementary file 3 [file pone.0361586.s010.pdf]

Response to the editor and reviewers of PONE-D-26-16141 A self-supervised reconstruction framework for segment-level detection of task-induced deviations in multi-channel EEG.

We thank the editor and the six reviewers for their careful and thoughtful examination of our paper. We appreciate the time and effort they have put into their reviews. Below, we provide answers to all comments. The main changes made to the manuscript are shown in red. We also proofread the whole text and made many changes (not shown in red) to correct some typos and improve the writing style for better readability.

August 6, 2026

1 Reviewer 1

Reviewer Comment

The core idea is sound, the pipeline is clearly described, and the use of a publicly available dataset is commendable.
The following comments are intended to help the authors bring the manuscript to a publishable state:

Reviewer 1 - Remark 1.1.

The most important issue to address is the artifact confound. The electrodes most frequently identified as exhibiting task-induced deviations, namely Fp1, Fp2, T3, and T5, are precisely those most susceptible to ocular, muscular, and movement artifacts during an active cognitive task compared to eyes-closed rest. The authors acknowledge in the pipeline description that detected outliers may reflect either genuine task-related neural activity or artifacts, but this distinction is never pursued in the analysis. The neurophysiological interpretations offered throughout the results and discussion therefore rest on an unverified assumption. The authors should include an explicit discussion of how artifact contamination may contribute to the observed electrode distribution, and ideally should apply an independent artifact characterization step, such as comparing deviant segment topographies against known artifact spatial signatures, to strengthen confidence in the neural interpretation.

Authors Remark 1.1.

We thank the reviewer for raising this important concern.
The dataset described in [1] consists of electroencephalographic (EEG) recordings from 36 participants, which were subjected by the original authors to Independent Component Analysis (ICA) in order to attenuate ocular, muscular, and cardiac activity. ICA is currently the most widely adopted technique for artifact correction in EEG research [2, 3]; however, it does not guarantee complete removal of residual artifacts, particularly at fronto-polar and lateral-temporal locations, which are precisely the electrode sites emphasized by the reviewer.
We agree with the reviewer that the Fp1, Fp2, T3, and T5 electrodes are particularly susceptible to contamination by ocular (Fp1/Fp2), temporal-muscle (T3/T5), and movement-related artifacts [2, 3].

Authors Remark 1.1.

To evaluate whether the identified outliers could be attributed to residual artifacts, we implemented a simple artifact-characterization procedure for each segment, based on two commonly used heuristics: the peak-to-peak (PTP) amplitude and the segment-wise signal kurtosis [2]. An outlier segment was labeled as artifactual when PTP $> 100\,\mu$V *or* kurtosis > 5. On average, only $5.4\% \pm 8.0\%$ of outlier segments fulfilled this criterion, suggesting that the large majority of detected deviations cannot be accounted for by high-amplitude or highly impulsive artifactual activity. Please see Section 3.3 Detection of mental task-induced deviations, lines 915 – 923 in the revised manuscript.

Authors Remark 1.1.

We acknowledge that, in the absence of concurrently acquired electrooculography (EOG) and electromyography (EMG) reference channels, the complete elimination of residual artifact contamination cannot be guaranteed. This represents an inherent limitation associated with the use of the investigated dataset [1], which we now explicitly state in the revised Section 3.3, lines 924 – 925.

We thank the reviewer for articulating this concern, which we believe substantially enhances the interpretation of our findings.

Reviewer 1 - Remark 1.2.

The statistical analysis requires attention in two areas. First, the large number of simultaneous tests conducted across metrics, electrodes, frequency bands, and subjects means that the reported p-values are likely inflated due to multiple comparisons. A correction procedure such as Benjamini-Hochberg false discovery rate control should be applied and the results re-reported accordingly. Second, the MAPE metric produces a mean of 420.63 with a standard deviation exceeding 11,000 and a maximum above one million during the arithmetic task, while the median remains below one. Describing this distribution using means and standard deviations is statistically inappropriate. The authors should either apply a log transformation before reporting summary statistics for this metric or replace it with robust alternatives such as median and interquartile range throughout.

Authors Remark 1.2.

We thank the reviewer for this remark.
To account for the large number of simultaneous statistical tests, we implemented the Benjamini–Hochberg false discovery rate (FDR) procedure to adjust all reported p-values for multiple comparisons. This multiple-testing correction has been applied consistently throughout the revised manuscript.
For the summary statistics of MAPE, we elected to report the mean and standard deviation, supplemented by three additional well-known measures: the 25th percentile (Q25), the 75th percentile (Q75), and the interquartile range (IQR). These statistics are computed for all metrics to provide a more comprehensive and robust characterization of their empirical distributions. Please see changes in the revised manuscript Section 3.1, Table 2.

Reviewer 1 - Remark 1.3.

The synthetic validation in Section 2.2.8 uses sinusoidal perturbations as the abnormal class. These produce deviations that are structurally very different from the subtle, broadband reconstruction errors expected from genuine task-induced EEG changes, and the near-perfect detection rates achieved by IF and LOF on synthetic data do not translate into meaningful evidence about the method's performance on real task segments. The authors should explicitly acknowledge this limitation and clarify what the synthetic validation is and is not intended to demonstrate.

Authors Remark 1.3.

We thank the reviewer for this remark.
We agree that sinusoidal perturbations are not representative of genuine task-induced EEG reconstruction errors. The synthetic validation presented in the Results section was never intended to emulate such task-related effects; its sole objective was to confirm that the anomaly detection methods (IF, LOF, and OCSVM) correctly identify samples from perturbed generated signals that fall outside the support of the training distribution within the proposed feature space.
We have revised Section 2.2.8, lines 677–679 and 692–695 to explicitly state this.

Reviewer 1 - Remark 1.4.

The OCSVM normal-sample accuracy of 64% with a standard deviation of 38% is poor and highly variable across subjects, indicating near-random classification for a substantial subset of participants. The subject-level deviant rate ranging from 1% to 86% compounds this concern. The authors should investigate whether subjects with high deviant rates correspond to those with poor reconstruction quality or limited training data, and should discuss what this variability implies for the reliability of the electrode-level conclusions.

Authors Remark 1.4.

We thank the reviewer for this remark.
To address the concern regarding the limited amount of training data, we computed Spearman's rank correlation coefficient between the per-subject deviant rate and the number of resting-state segments included in the training set, yielding $\rho = 0.04$ ($p = 0.81$). This result indicates no statistically significant or practically meaningful association between the volume of training data and the observed deviant rate.
To examine whether participants with higher deviant rates are those exhibiting poorer reconstruction performance, we computed Spearman rank-order correlations between the per-subject deviant rate and the mean value of each reconstruction metric, calculated over the normal (non-deviant) segments only. The analyses revealed no significant associations between the deviant rate and mean DTW ($\rho = -0.03$, $p = 0.89$), NC ($\rho = 0.05$, $p = 0.78$), PRD ($\rho = 0.24$, $p = 0.17$), STFTL ($\rho = 0.23$, $p = 0.18$), or SNR ($\rho = -0.09$, $p = 0.58$). The only statistically significant correlation was observed for MAPE ($\rho = 0.67$, $p < 0.001$). Collectively, these findings suggest that inter-subject variability in deviant rates cannot be attributed to differences in reconstruction quality on normal segments.
We recognize that this variability reflects authentic inter-individual differences in EEG responses elicited by mental arithmetic tasks. In the revised manuscript, we have incorporated a dedicated paragraph that elaborates on the implications of this variability for the interpretation and generalizability of our findings in Section 3.3, lines 873–888.

Reviewer 1 - Remark 1.5.

Finally, the manuscript contains two instances of duplicated words, specifically "suggesting suggesting" and "spatially spatially," and should be carefully proofread before resubmission.

Authors Remark 1.5.

We thank the reviewer for this remark. We fixed both repetitions and repetition of "this".

2 Reviewer 2

Reviewer Comment

Summary: This study presents a 1-second segment-based, self-supervised framework that utilizes a hierarchical variational autoencoder (hvEEGNet) and a One-Class SVM (OCSVM) to detect EEG signal deviations (anomalies) during mental arithmetic tasks. The study's aim to capture transient cortical dynamics by examining signals in short-term segments rather than analyzing them globally is scientifically valuable. However, there are very serious structural issues regarding methodological originality, the clear contribution to literature, the validity of the validation using synthetic data, and the reliability of the physiological interpretations that must absolutely be addressed before acceptance for publication. Major Comments:

Reviewer 2 - Remark 2.1

Weak Contribution to the Literature and Lack of Methodological Originality: The authors use the "hvEEGNet" architecture for signal reconstruction, and the text explicitly states that this architecture was already developed by Cisotto et al. (Ref [24]). The primary methodological contribution of this study appears to consist merely of adding metrics such as DTW, MAPE, PRD, STFTL, and SNR to the outputs of an existing model to perform anomaly detection with an OCSVM algorithm. The authors must go beyond simply testing an existing model on a new dataset; they must provide strong theoretical proof of why their methodology is structurally superior or more innovative than previous approaches (e.g., those using only kNN or simple thresholding).

Authors Remark 2.1.

We thank the reviewer for this important remark. We agree that hvEEGNet was originally developed by Cisotto et al., who also used DTW together with a kNN-based outlier criterion. We have therefore revised the manuscript to make clear that the contribution of the present study does not lie in proposing a new reconstruction architecture.

The contribution is instead a new methodological formulation that transforms reconstruction errors into a multidimensional, segment-level, one-class detection and spatial co-occurrence framework for identifying task-induced EEG deviations relative to an exclusively resting-state reference distribution.

More specifically, the contribution comprises four elements. First, we formulate the problem of detecting physiologically plausible, task-induced EEG deviations rather than corrupted, saturated, or otherwise invalid signals. Under this formulation, an anomaly is a statistically deviant EEG segment whose reconstruction-error profile differs from the distribution learned during rest; it is not interpreted as a clinical abnormality or a conventional signal artefact. Second, we construct a multi-metric reconstruction-error representation comprising DTW, NC, MAPE, PRD, STFTL, and SNR. These metrics capture complementary temporal, spectral, amplitude, correlation, and relative-error characteristics of the reconstructed signal. Third, we employ a label-free one-class learning strategy trained exclusively on resting-state EEG, without using task-condition labels during training. Fourth, we introduce a channel-level co-occurrence analysis to determine whether detected deviations occur simultaneously across electrodes, thereby providing a spatial and network-level characterisation of task-related neural activity.

Thus, hvEEGNet is used as the reconstruction backbone, but neither the architecture nor the individual metrics constitute the contribution in isolation. The novelty lies in their integration within a distinct problem formulation and analysis framework for label-free, segment-level detection of task-induced EEG deviations.

These contributions are now stated explicitly in the Introduction (lines 109–130) of the revised manuscript. We also refer the reviewer to our response to Remark 5.2, where the novelty is summarised concisely.

Reviewer 2 - Remark 2.2

Circularity in Validation with Synthetic Data: In Section 2.2.8, the authors state that they generated synthetic data using the already trained hvEEGNet model to validate the anomaly detection algorithms (OCSVM, IF, LOF). Validating an anomaly detection algorithm tested on synthetic data generated by a VAE (hvEEGNet) itself, using its own reconstruction errors, is methodologically flawed (a circular logic error). The VAE will transfer its own learned biases to the synthetic data. This does not scientifically prove that the model can capture real-world physiological anomalies; it merely shows that the model validates itself.

Authors Remark 2.2.

We acknowledge the reviewer's concern and would like to clarify that the validation procedure does not involve circular reasoning.
hvEEGNet is trained exclusively using the soft-DTW reconstruction loss. The six reconstruction-error metrics employed for anomaly detection (DTW, NC, MAPE, PRD, STFTL, SNR) are computed only after training has been completed and are never incorporated into the optimization objective. This methodological separation ensures that the anomaly-detection features do not inherit or reflect any biases induced by the training process.
We agree that this experiment does not allow us to demonstrate sensitivity to authentic, physiologically plausible task-related deviations, because the synthetic anomalies were implemented as sinusoidal perturbations rather than as realistic EEG alterations. As already clarified in our response to Remark 1.3 and now explicitly stated in Section 2.2.8, the exclusive objective of this validation is to confirm that the detectors correctly reject samples that fall outside the support of the resting-state distribution in the proposed feature space, rather than to assess sensitivity to genuine cognitive anomalies.

Reviewer 2 - Remark 2.3

Physiological Interpretations and the Possibility of Artifacts (Noise): The study finds that statistical deviations occur more frequently in peripheral electrodes such as Fp1, Fp2, T3, and T5. It is well known in the literature that Fp1 and Fp2 are highly susceptible to blinking/eye movements (EOG), while T3 and T5 are highly susceptible to jaw or neck muscle tension (EMG). Although the authors claim the dataset used is "artifact-free," involuntary muscle contractions (e.g., frowning) can occur during tasks requiring high cognitive effort, such as mental arithmetic. The authors must discuss in detail whether these detected deviations are truly complex cognitive processes or physiological artifacts (muscle tension) caused by increased mental effort.

Authors Remark 2.3.

We appreciate the reviewer's remark, which partially overlaps with Remark 1.1 raised by Reviewer 1. We respectfully refer the reviewer to our detailed response to Remark 1.1, in which we (i) describe the ICA-based preprocessing applied to the dataset by the original authors, (ii) present an artifact-characterization procedure based on peak-to-peak amplitude and kurtosis, demonstrating that only $5.4\% \pm 8.0\%$ of outlier segments satisfy standard artifact criteria, and (iii) explicitly acknowledge the lack of simultaneous EOG/EMG recordings as a limitation of the dataset. Accordingly, no additional modifications to the manuscript are deemed necessary beyond those already specified in response to Remark 1.1.

Reviewer 2 - Remark 2.4

Correlation of Metrics and Dimensionality (Curse of Dimensionality): A vector of 6 different metrics (DTW, NC, MAPE, PRD, STFTL, SNR) is provided as input to the OCSVM for anomaly detection. Error metrics such as MAPE and PRD generally show a very high correlation with each other. This situation can lead to unnecessary dimensionality in the model. The authors should present the correlation matrix between these 6 features and increase the robustness of the model by eliminating redundant features.

Authors Remark 2.4.

We thank the reviewer for this remark.
We computed the correlation matrix among the six reconstruction-error metrics for the arithmetic task. The analysis indicated that the only pair exhibiting a strong correlation was the PRD in the time domain and STFTL in the time–frequency domain ($\rho = 0.87$), consistent with the fact that both metrics are primarily sensitive to amplitude- and spectral-related distortions in the reconstructed signal.
We elected to retain all six metrics. Although PRD and STFTL exhibit a high degree of correlation, they quantify complementary characteristics of the reconstruction error (time-domain versus time-frequency distortion). Preserving both metrics therefore maintains interpretability with respect to the domain in which a given deviation predominantly arises. Furthermore, only one of the fifteen feature pairs exceeds the correlation threshold ($\rho = 0.80$).
We have incorporated an additional paragraph into Section 2.2.3 (lines 448-456) of the revised manuscript, in which we report this correlation analysis and justify the decision to retain all six metrics. This is presented in conjunction with the newly added Supplementary Fig. S7.

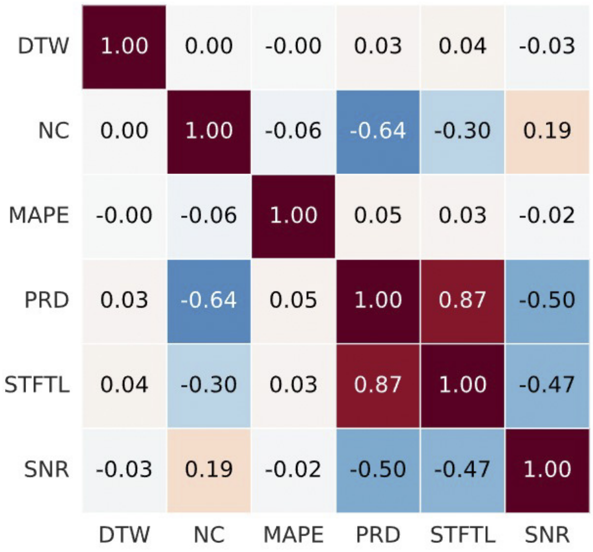

Figure 1: Pairwise Pearson correlation matrix of the six reconstruction-error metrics.

Section-by-Section Comments:

Reviewer 2 - Remark 2.5

Introduction: The introduction states that previous studies found contradictory results on the same dataset. However, it is not fully justified why the proposed "1-second segment" approach will physiologically resolve these contradictions in the literature. Window size is very critical in signal processing; why were exactly 1-second windows chosen instead of 2 seconds or 0.5 seconds? The signal processing or physiological foundations of this choice must be explained.

Authors Remark 2.5.

We thank the reviewer for this insightful comment. This point is addressed in detail in Remarks 4.2. and 4.3. We respectfully refer the reviewer to the our answers therein.

Reviewer 2 - Remark 2.6

Methods: How the hyperparameters of the One-Class Learning algorithms (OCSVM, IF, LOF) (e.g., kernel type or nu parameter for OCSVM, number of trees for IF) were selected and optimized is left missing in the methodology. The hyperparameter optimization process must be detailed.

Authors Remark 2.6.

We thank the reviewer for this remark.
We acknowledge that no systematic hyperparameter optimization procedure (e.g., grid search or cross-validation) was conducted for the one-class learning algorithms. The OCSVM, IF, and LOF methods were employed using the default configurations provided by scikit-learn and were applied uniformly across all subjects: OCSVM with a radial basis function (RBF) kernel, $\nu = 0.1$, and $\gamma =$ 'scale'; IF with 100 estimators and an automatically determined contamination parameter; and LOF with 20 neighbors, the Minkowski (Euclidean) distance metric, and automatically determined contamination [4]. The specific hyperparameter settings have been included in the revised Methods section (Lines 547–551). Furthermore, we now explicitly state, as a limitation, that a systematic hyperparameter optimization strategy (for example, cross-validation restricted to resting-state data) could potentially enhance detection performance and should therefore be addressed in future investigations (Lines 1066–1068).

Reviewer 2 - Remark 2.7

Results: In Section 3.4, the spatial differences (electrode-based) between "good quality counters" and "poor quality counters" are discussed. However, it is not specified in the Methods section exactly what criteria were used to define these subject groups in the dataset, how they were separated, and how many patients are in each group.

Authors Remark 2.7.

We thank the reviewer for this valuable remark. The categorization into “good quality counters” and “poor quality counters” follows the definition provided by the original dataset authors and is based on arithmetic task performance, whereby participants who required disproportionately high effort to complete the mental task were classified as poor counters. Specifically, participants were divided into Group “G” (24 subjects), characterized by good-quality performance (mean number of operations per 4 minutes = 21, SD = 7.4), and Group “B” (12 subjects), characterized by poor-quality performance (mean number of operations per 4 minutes = 7, SD = 3.6). We have incorporated this information, together with the corresponding citation to [1], into Section 2.1 (Dataset; Lines 180-187) to clarify the classification criteria and group sizes referenced in Section 3.4.

Reviewer 2 - Remark 2.8

Conclusion: The conclusion section should concretely state how the findings align with (or resolve) previous contradictory findings in the literature. Furthermore, the inferences regarding the practical clinical or neurological benefits of the article remain weak and should be strengthened.

Authors Remark 2.8.

We thank the reviewer for this remark. We have expanded the Conclusion (Lines 1044-1057) to explicitly state how our findings may reconcile the discrepancies reported across previous studies analysing the same dataset, and to strengthen the discussion of the clinical and neurological implications of the observed prefrontal/temporal involvement and its relevance to cognitive-related task analysis.

3 Reviewer 3

Reviewer Comment

This paper proposes a self-supervised framework for segment-based anomaly detection using deep learning to identify EEG signal outliers during mental arithmetic. 1) The reconstruction error metrics across segments from all subjects reported in table 2 show that outliers do exist during the arithmetic task. The experimental results support the manuscript's conclusions and the manuscript is technically sound. 2) The statistical analysis based on non-parametric Mann–Whitney U test suggests that for five of all six metrics, reconstruction errors were significantly higher during the arithmetic task than in the resting state with $p < 0.001$. The statistical analysis was performed appropriately and rigorously. 3) The data used in study is the EEG dataset of Mental Arithmetic Task Performance, that is publicly available. 4) The article is well organized and written in standard English.

Reviewer 3 - Remark 3.1

The reconstruction error metrics across segments from all subjects reported in table 2 show that outliers do exist during the arithmetic task. The experimental results support the manuscript's conclusions and the manuscript is technically sound.

Authors Remark 3.1

We thank the reviewer for this encouraging remark.

Reviewer 3 - Remark 3.2

The statistical analysis based on non-parametric Mann–Whitney U test suggests that for five of all six metrics, reconstruction errors were significantly higher during the arithmetic task than in the resting state with $p < 0.001$. The statistical analysis was performed appropriately and rigorously.

Authors Remark 3.2

We thank the reviewer for this encouraging remark.

Reviewer 3 - Remark 3.3

The data used in study is the EEG dataset of Mental Arithmetic Task Performance, that is publicly available.

Authors Remark 3.3

We thank the reviewer for this remark.

Reviewer 3 - Remark 3.4

The reviewer has one concern: where are the Figures 4 and 5?

Authors Remark 3.4

In the original manuscript, figures 4 and 5 were included at lines 721–722 and 801–802, respectively. In the revised manuscript, they appear at lines 965–966 and 1073–1074, respectively.

4 Reviewer 4

Reviewer Comment

The article presents work on a matter of high interest on the EEG comunity.
Before discussing the technical side, an important point to address is the length of the article. It is excesive for the topic and the information produced. Several parts of the article re-write the same ideas with different formats without introducing any new information to the reader. I would request the authors to thighten the text, introduce every idea once in the correct section and refer to the sections when required. Also, the authors should improve the use of references, not only as support of the considerations and results in this article but also to dedicate the space to the important new findings and not information that is well known or easily referenced such as the error metrics.
Some more specific coments below:

Reviewer 4 Remark 4.1

Introduction: Ensure to define the achronims the first time they are used, example SVM, used and defined on the second instance, not the first. others like NC and PRD must be corrected as well.

Authors Remark 4.1

We thank the reviewer for this valuable comment. We have revised the manuscript accordingly and corrected several acronyms throughout the text.

Reviewer 4 Remark 4.2

Introduction: the authors describe the use of 1sec segments. What lead to this selection? Explain clearly how shortter and longer segments would affect the results, and how the authors make the results not sensitive to the segment length. A proper study on this topic must be presented.

Authors Remark 4.2

We thank the reviewer for this remark.
This study uses 1-second analysis windows, which are widely recognized as an effective compromise that balances several competing methodological constraints: (i) preserving approximate signal stationarity, (ii) retaining behaviorally and physiologically meaningful temporal dynamics, (iii) achieving adequate spectral resolution given the sampling frequency, (iv) facilitating robust detection and rejection of artifacts, and (v) ensuring a sufficient number of observations to support subsequent statistical inference and machine learning–based analyses.

Authors Remark 4.2

While increasing the duration of these 1-second segments can enhance frequency resolution, it simultaneously aggravates violations of the underlying stationarity assumptions and reduces temporal specificity [5–9].
We have incorporated an additional paragraph into Section 2.2.1 Signal segmentation.

Reviewer 4 Remark 4.3

Metholodogy: The authors describe the process including that "the signal is divided into non-overlapping...", what are the effect of data bining on the signal processing results? what are the effects of overlapping? what are the edge effects specially in frequency domain?

Authors' Response to Remark 4.3

We thank the reviewer for this important remark. We have expanded the signal-segmentation subsection to clarify the effects of non-overlapping and overlapping windows, temporal binning, and boundary effects in the frequency domain.
Continuous EEG recordings are commonly partitioned into either non-overlapping or overlapping epochs before extracting temporal, spectral, nonlinear, or machine-learning features. Non-overlapping segmentation divides the signal into consecutive windows without reusing samples across epochs. This avoids the additional dependence introduced by overlap, although neighbouring EEG segments may still remain temporally correlated because of the intrinsic autocorrelation of the signal. Since each sample contributes to only one epoch, non-overlapping segmentation also reduces the risk of inflating the effective sample size and limits data leakage in machine-learning pipelines. Its principal drawback is that, for a fixed recording duration, it yields fewer epochs than overlapping segmentation, which may increase the variability of spectral estimates and reduce the temporal density with which transient changes can be tracked.
Overlapping segmentation reuses subsets of EEG samples across neighbouring epochs. This increases the number of analysable segments and improves temporal continuity, but direct sample reuse introduces additional dependence between epochs. If segments derived from the same recording are distributed across training, validation, or test sets without appropriate separation, model performance may be substantially overestimated because of data leakage. We also clarify in the revised manuscript that overlap between the main EEG epochs should be distinguished from the overlap used internally by spectral-estimation procedures. For example, Welch's method may employ overlapping tapered sub-windows within each EEG segment to reduce the variability of power spectral density estimates without requiring the main 1-second analysis epochs themselves to overlap.

Authors Remark 4.3

In the present study, we therefore use fixed, non-overlapping 1-second epochs. This choice avoids direct sample reuse between segments and limits the additional dependence that overlapping epochs would introduce between observations used for model fitting, validation, and evaluation. It also reduces the risk of data leakage between the resting-state segments used to train hvEEGNet and the segments held out for validation or analysed during the arithmetic task. We do not claim that non-overlapping EEG segments are strictly statistically independent.

The revised manuscript also explains the effect of temporal binning. Binning combines adjacent samples through averaging or another aggregation operation [6, 10]. Although this procedure may reduce high-frequency noise and improve the signal-to-noise ratio [11], it acts as a smoothing or low-pass operation and can suppress rapid temporal variations [12]. Excessive binning may therefore remove physiologically relevant transients, reduce temporal specificity, and attenuate beta- and gamma-band content [13–15]. No temporal binning, sample averaging, or additional downsampling was applied in this study. Each 1-second epoch retains the original sampling resolution of 500 Hz, corresponding to $N = 500$ samples.

Finally, we have clarified the treatment of edge effects. Finite EEG epochs may exhibit spectral leakage, amplitude distortion, and spurious oscillations when spectral, wavelet, Hilbert-transform, or filtering operations are applied near their boundaries [14, 16–19]. Common mitigation strategies include padding, tapered analysis windows, symmetric extension, and exclusion of boundary samples [6, 12, 14, 20]. In our implementation, the main EEG segmentation uses rectangular, non-overlapping 1-second epochs because hvEEGNet reconstructs each segment end-to-end. Boundary mitigation is applied in the subsequent spectral computations: the power spectral density used for the SNR metric is estimated with Welch's method using tapered overlapping sub-windows, while the STFTL metric uses a tapered Gaussian analysis window. These choices reduce spectral leakage at the boundaries of the 1-second epochs.

These clarifications have been added to the revised manuscript in the signal-segmentation subsection, lines [264–362].

Reviewer 4 Remark 4.4

Signal segmentation: What is the correlation between outlier detection on different channels? Although the assumption is that there is no influence of deviations on one channel onto other chanels, present the prove that this thesis is correct. In most life signals detection there is some level of influece between channels and the results are presented under the limitations of these influences and thresholds are presented, not mathematically but physically. Since the authors ensure that the multichannel reconstruction is appropriate with zero cross-channel influence, the data showing this is true is necessary, otherwise clearly present the limitations.

Authors' Response to Remark 4.4

We thank the reviewer for this important remark, which allows us to clarify the meaning of channel independence in the proposed framework.

The independence described in the manuscript concerns the *detection procedure*. For a given channel, the reconstruction-error feature vector and the resulting outlier classification are computed exclusively from the original and reconstructed signals of that channel, without incorporating reconstruction-error features from any other channel. This procedural separation is guaranteed by the design of the detector. It does not imply that EEG activity, reconstruction errors, or outlier labels are statistically independent across channels.

We have revised the manuscript to make this distinction explicit. The use of non-overlapping 1-second segments prevents direct sample reuse between consecutive epochs and avoids the additional dependence that overlapping windows would introduce. However, non-overlapping segments may still exhibit temporal autocorrelation, and channels may remain statistically associated because they reflect shared or coordinated neural processes.

To examine cross-channel dependence at the level of the detected outcomes, we introduced a permutation-based analysis of the channel-level outlier-label co-occurrence structure, as described in Sections 2.2.6 and 3.5. For each participant, the temporal order of the outlier labels was independently permuted within each channel over 1,000 iterations. This procedure preserves the marginal outlier frequency of each channel while removing the observed temporal alignment of outlier events across channels. The empirical pairwise co-occurrence counts were then compared with the corresponding permutation-based null distributions, with false discovery rate correction applied across channel pairs.

Under this analysis, 62.0% of channel pairs exhibited co-occurrence rates significantly greater than expected under the permutation null model. This result shows that the detected outlier labels are not statistically independent across channels and that coordinated deviations occur more frequently than expected from the channel-specific marginal outlier rates alone. The finding is consistent with the co-occurrence and graph-based analyses presented in Section 3.5.

We emphasise that this analysis demonstrates statistical association rather than causal influence between channels. The proposed framework does not assume zero physiological interaction or zero statistical dependence between electrodes. Instead, it separates channel-wise detection from the subsequent analysis of cross-channel co-occurrence. This distinction and the associated limitation are now stated explicitly in Sections 2.2.5 (Lines 510–518) and 2.2.6 (lines 584–597) of the revised manuscript).

Reviewer 4 Remark 4.5

- Reconstruction metrics: Unnecessary long. The text must be shortened and optimized. the table is useful, but refer the reader to the adequate references and use equations that absolutely necessary for the understanding of the work.

Authors Remark 4.5

We appreciate the reviewer's insightful remark. The reconstruction metrics are deliberately formulated from a pedagogical standpoint, with the aim of addressing not only readers who already possess advanced expertise in the subject matter, but also students who are at an early stage of their academic training.

Reviewer 4 Remark 4.6

Experimental setup and design rationale: Many ideas are just a repetition of prior sections. Consider a deep review of this section, some information, specially from the first few paragraphs belong to the introduction of the paper.

Authors Remark 4.6

We thank the reviewer for this remark.
Following this recommendation, we carried out a revision of Section 2.2.7 (Experimental setup and design rationale). Two paragraphs whose content was found to be either repetitive or more suitable for the Introduction have been removed from this section and relocated accordingly: (i) the paragraph comparing global versus segment-level EEG analysis approaches has been split with the general concept moved to Introduction (lines 81–85) and the details moved to Section 2.2.5 (lines 264–270), which has been restrcutured for better readability; and (ii) the paragraph motivating the channel-wise detection strategy has been moved to the Introduction (lines 131–144). Section 2.2.7 now contains only the method summary of the experimental configuration, including the training/validation split, segment parameters, and the one-class classification step, without duplicating material already introduced in earlier sections.

Reviewer 4 Remark 4.7

Section 2.2.7 Present supporting information regarding the window size, what would be the minimum N value that can be used?

Authors Remark 4.7

We thank the reviewer for this insightful comment. This issue is discussed in detail in our answers to Remarks 4.2 and 4.3.
In electroencephalographic (EEG) signal processing, the minimum analysis window duration is governed by the lowest frequency component of interest rather than by a fixed, universally applicable number of samples N. As a practical rule of thumb, the analysis window should contain at least $3 - 5$ cycles of the slowest oscillatory component to obtain reliable spectral and phase estimates. Consequently, the required number of samples N is determined jointly by the sampling frequency and the lower cutoff of the frequency band under examination. Consequently, the minimal admissible window duration is determined directly by the sampling frequency of the signal and the properties of the target frequency band. Within this framework, advancing the analysis window in increments of 1 second is, by definition, equivalent to shifting it by one sample [14].

Reviewer 4 Remark 4.8

Section 2.2.7 Overlapping: find the correct section for the discussion and ensure to present mathematical prove on why this should be avoided or use adquate references.

Authors Remark 4.8

We thank the reviewer for this insightful comment. This issue is discussed in detail in our answers to Remarks 4.2 and 4.3.
To the best of our knowledge, there exists no universal mathematical theorem that dictates overlapping windows must invariably be avoided. On the contrary, window overlap is theoretically optimal in certain contexts (e.g., Welch's method for power spectral density estimation), whereas it may be deleterious in others (e.g., statistical inference and procedures that rely on independence assumptions). This distinction is well established in signal-processing theory and EEG methodology [6, 12, 14, 17, 21, 22].

Reviewer 4 Remark 4.9

Section 2.2.8 las paragraph: Repetition of the methodology described in previous section. Same with paragraph 1 page 14.

Authors Remark 4.9

We thank the reviewer for this insightful comment. We acknowledge the repetition of these sentences; it is intentional and serves to guide the reader through the argumentation in a stepwise manner.

Reviewer 4 Remark 4.10

The motivation should be addressed in the introduction of the paper, not in a later section, unless there is a direct influence in the results that the reader has to be reminded of, which is not happening in page 14.

Authors Remark 4.10

We thank the reviewer for this remark.
We respectfully refer the reviewer to our answer to Remark 4.6. Upon revision, we identified that two paragraphs in Section 2.2.7 (Experimental setup and design rationale) are more appropriately placed in the Introduction and partly to Section 2.2.5. Section 2.2.7 now contains only the operational summary of the experimental configuration, including the training/validation split, segment parameters, and the one-class classification step.

Reviewer 4 Remark 4.11

Section 2.2.8: Although sinusoidal signals are a good start of the perturbation study, even with the use of random frequency components, the observations can be quite limited. I strongly encourage the authors to include other type of signals such as wavelets, boadband gaussian noise, modified sinusoidal oscillation with gaussian tappering , non-stationary colored noise, frequency bursts (specially high freq), etc. This would ensure the results are sound and the methodology is tested against challenging and more realistic perturbations

Authors Remark 4.11

We appreciate the reviewer's comment, which aligns with the concerns previously raised by Reviewer 1 (Remark 1.3) and Reviewer 2 (Remark 2.2) regarding the breadth and adequacy of the synthetic validation.
We agree that sinusoidal perturbations, even when their frequency, amplitude, phase, and offset are randomized, constitute only a single class of synthetic deviation and do not encompass the full spectrum of possible signal morphologies (e.g., transient bursts, broadband or colored noise, non-stationary perturbations) that could be used to more extensively stress-test the detectors. As clarified in our response to Remark 1.3, the synthetic validation was not designed to demonstrate sensitivity to physiologically realistic, task-induced deviations. Its sole objective was to verify that the one-class detectors (OCSVM, IF, LOF) reliably reject samples lying outside the support of the resting-state distribution within the proposed six-dimensional feature space. For this methodological purpose, the current perturbation scheme is adequate.
We regard the systematic investigation of additional perturbation families—including wavelet-based transients, broadband and non-stationary colored noise, tapered oscillatory bursts, and high-frequency transients—as an important avenue for future research. We have accordingly incorporated a sentence to this effect in Section 2.2.8 and in the Conclusion.

Reviewer 4 Remark 4.12

Section 3.1.: The authors describe how different metrics were less consistent across subjects, either the authors assess the meaning of this conclussion or eliminate the non-consistent metrics from the analysis, with suporting work.Some questions that could guide the process with regards to the observations: Are the metrics used incorrect for the study? or do the observations determine that other underlying effects may be affecting the results and the current metrics are insufient and more work is necessary?

Authors Remark 4.12

We thank the reviewer for this valuable and insightful comment. The observed variability does not, in itself, demonstrate that these metrics are inappropriate. Instead, it may reflect genuine inter-individual neurophysiological variability, sensitivity to specific preprocessing pipelines, or the impact of unmeasured biological or experimental covariates. Accordingly, the results indicate that further targeted analyses are necessary to disentangle whether the observed variability primarily reflects measurement limitations, underlying physiological heterogeneity, or distinct and potentially complementary aspects of the signal captured by the different metrics. Therefore, we argue that these metrics should not be discarded solely based on reduced consistency, unless their reliability or methodological robustness can be shown to be insufficient. There exists a substantial literature that examines this topic in depth, for example [14, 15, 23, 24]

Reviewer 4 Remark 4.13

Table 2, could better be a figure that sompare the resting state with the arithmetic task, although the information is there, the task of drawing a full picture of the results is left to the reader.
Standard deviation, results are presented numerically, the meaning of these results is not addressed.
This section lacks a strong conclussion, are the metrics and methodology used able to discriminate between resting state and brain activation due to arithmetic tasks? Also are the metrics used in this study enogh to fulfill the rquirements of discrimitation and identification of abnormal data?

Authors Remark 4.13

We thank the reviewer for these three closely related observations, which we address in turn.

We agree that a visual representation facilitates the interpretation of Table 2. A direct comparison of the reconstruction-error distributions across the two conditions is already provided in the revised manuscript: Fig. 2b displays, for each subject, the per-condition distribution of each metric, and Supplementary Fig. S3 shows the complete per-subject breakdowns for all metrics. Together, these figures allow the reader to perceive the systematic shift in reconstruction error between conditions and to appreciate its inter-subject variability, complementing the numerical summaries in Table 2. Please see Figs. 2b and S3 in the revised manuscript.

The standard deviations reported in Table 2 carry different interpretive weight depending on the metric. For metrics with bounded or well-behaved distributions (NC, SNR, PRD, DTW), the SD quantifies the spread of reconstruction errors across segments and subjects in a conventional sense: a larger SD indicates greater variability in how faithfully the model reconstructs individual EEG segments, reflecting genuine inter- and intra-subject heterogeneity in signal structure. For MAPE, however, the SD is dominated by a small fraction of segments for which the reference signal passes through or near zero, causing the denominator to collapse and the ratio to become arbitrarily large; as discussed in our response to Remark 1.2, the median and interquartile range (Q25–Q75) are therefore the more informative summary statistics for this metric. More broadly, the markedly larger SDs observed during the arithmetic task compared to rest reflect a well-documented feature of EEG responses to cognitive load: not all subjects exhibit the same degree of cortical engagement, and not all segments within a given subject deviate from the resting-state baseline to the same extent. The SD thus serves as a direct metric of physiological heterogeneity rather than of measurement noise, and its inflation during the arithmetic condition is itself a meaningful finding consistent with the between-subject variability in deviant rates discussed in Section 3.3.

The non-parametric Mann–Whitney U test demonstrated that reconstruction errors were significantly higher during the arithmetic task than during rest for five of the six metrics ($p < 0.001$, FDR-corrected), providing strong statistical evidence that the proposed feature space captures a systematic difference between the two conditions. The metrics and methodology are therefore able to discriminate between resting-state activity and task-induced brain activation at the population level. At the single-subject level, the OCSVM classifier further identifies which individual segments deviate beyond the resting-state distribution, offering a temporal and channel-level resolution that global statistics cannot provide. The combination of group-level significance and subject-level anomaly scores thus jointly fulfils the dual requirement of discriminating between conditions and identifying the abnormal data segments. We have added a concluding sentence to this effect at the end of Section 3.1 in the revised manuscript. Please see Section 3.1 in the revised manuscript.

Reviewer 4 Remark 4.14

Section 3.5 is interesting, but the results seem inconclusive, is the aim of the authors to tie these resulst with those of the previous sections? If that is the intent, a review of the text and data presentation should be considered.

Authors Remark 4.14

We thank the reviewer for this observation. Section 3.5 is indeed intended to connect the co-occurrence results to those of the preceding sections. The low inter-channel correlation during deviant events (0.46 ± 0.07 vs. 0.54 ± 0.06 at baseline; $p < 0.001$) indicates that task-induced deviations are transient and partially asynchronous, which directly motivates the single-channel detection strategy. To make this integrative role explicit, we have revised the opening paragraph of Section 3.5 accordingly (lines 967-973).

5 Reviewer 5

Reviewer Comment

The manuscript presents a self-supervised framework based on hvEEGNet for detecting segment-level deviations in EEG recordings during mental arithmetic tasks. The topic is timely and relevant, particularly because the study attempts to address inconsistencies in prior analyses of the same EEG dataset by focusing on transient segment-level deviations instead of global EEG characteristics. The integration of reconstruction metrics (DTW, STFTL, SNR, etc.) with one-class anomaly detection is methodologically interesting and potentially useful for EEG analysis where labelled datasets are unavailable. The manuscript is technically ambitious and generally well organized. However, there are several methodological, statistical, interpretational, and presentation-related weaknesses that substantially limit the strength of the conclusions.

Reviewer 5 Remark 5.1

The authors are suggested to include a artifact robustness analysis to demonstrate detected deviations persist aftrer artifact rejection procedure.

Authors Remark 5.1

We thank the reviewer for this remark, which overlaps with Remark 1.1 raised by Reviewer 1. We refer the reviewer to our detailed response to Remark 1.1, where we (i) describe the ICA preprocessing applied to the dataset by the original authors, (ii) present an artifact-characterization step based on peak-to-peak amplitude and kurtosis showing that only $5.4\% \pm 8.0\%$ of outlier segments meet standard artifact criteria, and (iii) explicitly acknowledge the absence of simultaneous EOG/EMG channels as a limitation. No additional changes to the manuscript are required beyond those described in Remark 1.1.

Reviewer 5 Remark 5.2

The authors should elaborate the novelty concisely and justy the work is not a stacked piece of earlier works.

Authors Remark 5.2

We thank the reviewer for this remark. We have revised the Introduction (lines 109-130) to state the contribution of the work explicitly and to clarify that the proposed method is not merely a stacking of previously published components.
The contribution is not a new reconstruction architecture, but a new methodological formulation that transforms reconstruction errors into a multidimensional, segment-level, one-class detection and spatial co-occurrence framework for identifying task-induced EEG deviations relative to an exclusively resting-state reference distribution.
The framework makes four specific contributions: (i) a new problem formulation for detecting physiologically plausible task-induced EEG deviations at the segment level, without using task-condition labels; (ii) a multi-metric reconstruction-error representation comprising DTW, NC, MAPE, PRD, STFTL, and SNR, which captures complementary temporal, spectral, amplitude, correlation, and relative-error characteristics; (iii) a label-free one-class classification strategy trained exclusively on resting-state EEG; and (iv) a channel-level co-occurrence analysis that determines whether detected deviations arise simultaneously across electrodes, providing a spatial and network-level perspective on task-related neural activity.
Although individual components such as hvEEGNet, DTW, and one-class classification have been used previously, their role in the present study is defined by this distinct problem formulation and integrated methodological framework. These contributions are now stated explicitly in the Introduction of the revised manuscript.
We also refer the reviewer to our response to Remark 2.1, where the distinction between the proposed contribution and the original hvEEGNet method is discussed in greater detail.

Reviewer 5 Remark 5.3

The concept of “outlier,” “anomaly,” and “task-induced deviation” is used somewhat interchangeably throughout the manuscript. The authors should be aware and improve the technical clarity of the manuscript.

Authors Remark 5.3

We thank the reviewer for this remark. We agree that using these terms without explicit clarification could create ambiguity. We have added the following sentence in the Introduction, at the paragraph where all three terms first appear together: *“In this study, EEG segments identified as outliers from their reconstruction-error profiles are termed anomalies in a strictly statistical sense; the term does not denote clinical abnormality. Accordingly, the terms anomaly, outlier, and task-induced deviation refer to physiologically plausible EEG segments that differ significantly from the resting-state reference distribution.”* This statement is highlighted in red in the revised manuscript in Lines 138-142.

Reviewer 5 Remark 5.4

The English is generally understandable but occasionally awkward and overly formal. Several sections of the manuscript would benefit from professional language editing.

Authors Remark 5.4

We thank the reviewer for this valuable recommendation. Accordingly, the manuscript has been revised to minimize the use of formalism.

Reviewer 5 Remark 5.5

The manuscript lacks comparison with some baseline methods or prior established works. Without these comparisons, it is difficult to quantify the true advantage of the proposed framework. The authors should try simpler reconstruction methods at least.

Authors Remark 5.5

We thank the reviewer for this valuable remark. To the best of our knowledge, no standardized benchmark is currently available for this particular reconstruction problem, except for [25], who is referenced strongly in our work. Therefore, rather than conducting comparisons with methods evaluated under heterogeneous experimental conditions, we chose to evaluate the proposed framework via extensive internal validation and by comparison with simple baseline reconstruction methods that are representative of widely used EEG signal-processing approaches.
Regarding the three studies mentioned in our Introduction ([26–28]), these address a fundamentally different problem: they train supervised classifiers on globally-extracted features to discriminate a priori labelled rest/task or good/poor-counter conditions, reporting classification accuracy or F1-score. These metrics are not comparable with the unsupervised, segment-level reconstruction-error-based analysis produced by our framework, since our approach involves neither a predefined classification task nor ground-truth outlier labels. Reporting their published accuracy/F1 figures alongside our reconstruction-error statistics in a single comparative table would therefore combine results obtained under different tasks and evaluation metrics, rather than provide a meaningful quantitative comparison. We have expanded the relevant paragraph in the Introduction (Lines 54-64) to make this methodological distinction explicit.

6 Reviewer 6

Reviewer Comment

PONE-D-26-16141, “A self-supervised reconstruction framework for segment-level detection of taskinduced deviations in multi-channel EEG”

Reviewer Remark 6.1

Abstract:
1. Please declare the electroencephalogram first before using the ”EEG” abbreviation in the second line.
2. Please remove the abbreviation ”hvEEGNet” because it is not used in the abstract.
3. Please remove the abbreviation ”DTW, STFT, SNR” because they are not used in the abstract.
4. Please review the tense used in describing the experimental methods and results, it is preferred to use past tense.

Authors Remark 6.1

We thank the reviewer for this remark. All suggestions (1, 2, 3, and 4) have been considered in the revised manuscript.

Reviewer Remark 6.2

Introduction section:
1. Please use (i.e. BA 46) instead of (BA 46). (line 17)
2. Please reorder the cited references to be [3] [10] instead of [10] [3]. (line 22)
3. Please declare ”SVM” first before using the abbreviation. (line 40)
4. Please declare both ”LDA and kNN” before using them. (line 51)
5. In line 74, what do you mean by ”...,which are not available for this dataset”, which dataset are you referring to?
6. Please declare ”hvEEGNet” first before using it. (line 88)
7. Please declare all ”NC, PRD, STFTL, and MAPE” first before using them. (line 94)
8. Please no need to use slanted font style in the paragraph started from line 108 to line 114.
9. Please remove the ”,” between segment-level and self supervised framework. (line 115)
10. In the last paragraph, all subsections of section 2 were mentioned except subsections 2.2.7 -2.2.9, please state them.

Authors Remark 6.2

We thank the reviewer for this remark. All suggestions (1 to 10) have been considered in the revised manuscript.

Reviewer Remark 6.3

Material and Methods section:
1. In section 2.1, please mention brief demographic information about the dataset used.
2. In section 2.2, have you calculated the training time for the proposed framework? if yes, please state the computation capabilities used and the average time consumed.
3. (line 176) please use (i.e. electrode) instead of (electrode).
4. (line 181) please use (i.e. each value....) instead of (each value....). Do the same throughout the paper. 5. Please remove the last sentence in the caption of Figure 1, as it already explained in the context. 1
6. (line 211) please use (Eq. 1) instead of (1) to refer to Equation 1.
7. In subsection 2.2.2, please enumerate all the equations before Equation 4.
8. (line 437 and 439) please use (N = 200) instead of (N = 200).
9. In the statistical analysis subsection, Did you test the normality of data using Shapiro-Wilk test first before using the Mann-Whitney U test? if, yes please state the test p-value.

Authors Remark 6.3

We thank the reviewer for this remark. Points 1, 3, 4, 5, 6, and 7 were amended. Regarding point 1, brief demographic information was added to Section 2.1: the dataset comprises 36 healthy university students (20 males, 16 females; mean age $\approx 20 \pm 1.5$ years). Regarding point 2, training times were not recorded during the experiments; we acknowledge this as a limitation. Regarding point 8, the reviewer's request refers to lines 437 and 439 in the original submission where $N = 200$ appears in plain text rather than in math mode; we have corrected this to $N = 200$ throughout. Regarding point 9, the Mann–Whitney U test is a non-parametric test that does not assume normality; a Shapiro–Wilk test was therefore not required as a prerequisite.

Reviewer Remark 6.4

Results and Discussion section:
1. In this section, it is recommended to use the past tense in reporting the findings.
2. (line 525) Please use "60 s" instead of "60 seconds". Please use "s" to refer to seconds throughout the paper.
3. (line 535) What is the unit of measurement of DTW that reached approximately 13000.
4. (line 578) What is the unit of measurement of the standard deviation that increased frome 3.08 to 13.69 and similarly for the gamma band.
5. I can't find figure 4 and 5 in the submitted file

Authors Remark 6.4

We thank the reviewer for this remark. The points 1,2,3, 4 were amended. Figures 4 and 5 were included in the submitted PDF and appear correctly in the revised manuscript.

References

[1] Zyma I, Tukaev S, Seleznov I, Kiyono K, Popov A, Chernykh M, et al. Electroencephalograms during Mental Arithmetic Task Performance. Data. 2019;4(1):14. doi:10.3390/data4010014.

[2] Quintero-Rincón A, D'Giano C, Batatia H. Artefacts Detection in EEG Signals. In: Advances in Signal Processing: Reviews. vol. 2. International Frequency Sensor Association Publishing; 2021.

[3] Ronca V, Capotorto R, Di Flumeri G, Giorgi A, Vozzi A, Germano D, et al. Optimizing EEG Signal Integrity: A Comprehensive Guide to Ocular Artifact Correction. Bioengineering. 2024;11(10). doi:10.3390/bioengineering11101018.

[4] Pedregosa F, Varoquaux G, Gramfort A, Michel V, Thirion B, Grisel O, et al. Scikit-learn: Machine Learning in Python. Journal of Machine Learning Research. 2011;12:2825–2830.

[5] Levy WJ. Effect of epoch length on power spectrum analysis of the EEG. Anesthesiology. 1987;66(4):489–495. doi:10.1097/00000542-198704000-00007.

[6] Oppenheim AV, Schafer RW. Discrete-Time Signal Processing. 2nd ed. Pearson; 2011.

[7] Quintero-Rincón A, Pereyra M, D'Giano C, Risk M, Batatia H. Fast statistical model-based classification of epileptic EEG signals. Biocybernetics and Biomedical Engineering. 2018;38(4):877–889. doi:10.1016/j.bbe.2018.08.002.

[8] Cerquera A, Vollebregt MA, Arns M. Nonlinear Recurrent Dynamics and Long-Term Nonstationarities in EEG Alpha Cortical Activity: Implications for Choosing Adequate Segment Length in Nonlinear EEG Analyses. Clinical EEG and Neuroscience. 2018;49(2):71–78. doi:10.1177/1550059417724695.

[9] Zimmermann M, Schultz-Nielsen K, Dumas G, Konvalinka I. Arbitrary methodological decisions skew inter-brain synchronization estimates in hyperscanning-EEG studies. Imaging Neuroscience. 2024;2:imag–2–00350. doi:10.1162/imag_a_00350.

[10] Shannon CE. Communication in the Presence of Noise. Proceedings of the IRE. 1949;37(1):10–21. doi:10.1109/JRPROC.1949.232969.

[11] Hu S, Ruan J, Valdes-Sosa PA, Lv Z. How do the resting EEG preprocessing states affect the outcomes of postprocessing? NEUROIMAGE. 2025;310:121122. doi:10.1016/j.neuroimage.2025.121122.

[12] Orfanidis SJ. Introduction to Signal Processing. Prentice Hall; 2010.

[13] Costa M, Goldberger AL, Peng CK. Multiscale entropy analysis of biological signals. Physical Review E. 2005;71:021906. doi:10.1103/PhysRevE.71.021906.

[14] Cohen MX. Analyzing Neural Time Series Data: Theory and Practice. The MIT Press; 2014.

[15] Luck SJ. An Introduction to the Event-Related Potential Technique. 2nd ed. MIT Press; 2014.

[16] Harris FJ. On the use of windows for harmonic analysis with the discrete Fourier transform. Proceedings of the IEEE. 1978;66(1):51–83. doi:10.1109/PROC.1978.10837.

[17] Percival DB, Walden AT. Spectral Analysis for Physical Applications. Cambridge University Press; 1993.

[18] Torrence C, Compo GP. A Practical Guide to Wavelet Analysis. Bulletin of the American Meteorological Society. 1998;79(1):61–78. doi:10.1175/1520-0477(1998)079¡0061:APGTWA¿2.0.CO;2.

[19] Widmann A, Schrӗer E, Maess B. Digital filter design for electrophysiological data – a practical approach. Journal of Neuroscience Methods. 2015;250:34–46. doi:10.1016/j.jneumeth.2014.08.002.

[20] Jaberi A, Tahsildoost M, Rezvani S, Ardekani A. A comprehensive review of neuroarchitecture measurement tools and methods. Building and Environment. 2026;289:114103. doi:10.1016/j.buildenv.2025.114103.

[21] Kay SM. Modern Spectral Estimation: Theory and Application. Prentice Hall; 1988.

[22] Roy Y, Banville H, Albuquerque I, Gramfort A, Falk TH, Faubert J. Deep learning-based electroencephalography analysis: a systematic review. J Neural Eng. 2019;16(5):051001.

[23] Altman DG. Practical statistics for medical research. London: CRC Press; 1991.

[24] James G, Witten D, Hastie T, Tibshirani R, Taylor J. An Introduction to Statistical Learning: with Applications in Python. Springer; 2023.

[25] Cisotto G, Zancanaro A, Zoppis IF, Manzoni SL. hvEEGNet: A novel deep learning model for high-fidelity EEG reconstruction. Frontiers in Neuroinformatics. 2024;18:1459970. doi:10.3389/fninf.2024.1459970.

[26] Khanam F, Ahmad M, Hossain ABMA. Investigation of the neural correlation with task performance and its effect on cognitive load level classification. PLOS ONE. 2023;18(12):1–24. doi:10.1371/journal.pone.0291576.

[27] Jafari Morteza M, Sarbaz Y, Zolfaghari S, Khodayarlou A. The influence of mental calculations on brain regions and heart rates. Scientific Reports. 2024;14(1):18846. doi:10.1038/s41598-024-69919-x.

[28] Nguyen PK, Huynh QL. A Machine Learning Approach in EEG-Based Assessment of Cognitive Load by Mental Arithmetic Tasks. Journal of Physics: Conference Series. 2025;2949(1):012004. doi:10.1088/1742-6596/2949/1/012004.
