# Supplementary material for: A self-supervised reconstruction framework for segment-level detection of task-induced deviations in multi-channel EEG
Source: PLoS One. 2026 Oct 30;21(10):e0361586. doi: 10.1371/journal.pone.0361586 (PMC13722333; doi:10.1371/journal.pone.0361586)
Supplement: Peer review history

# Peer review: Decision Letter 0

**DOI:** 10.1371/journal.pone.0361586.r001

**Authors:** Onder Aydemir

29 May 2026

Dear Dr. Batatia,

Thank you for submitting your manuscript to PLOS ONE. After careful consideration, we feel that it has merit but does not fully meet PLOS ONE’s publication criteria as it currently stands. Therefore, we invite you to submit a revised version of the manuscript that addresses the points raised during the review process.

Please submit your revised manuscript by Jul 13 2026 11:59PM. If you will need more time than this to complete your revisions, please reply to this message or contact the journal office at plosone@plos.org. When you're ready to submit your revision, log on to https://www.editorialmanager.com/pone/ and select the 'Submissions Needing Revision' folder to locate your manuscript file.

- A letter that responds to each point raised by the academic editor and reviewer(s). You should upload this letter as a separate file labeled 'Response to Reviewers'.
- A marked-up copy of your manuscript that highlights changes made to the original version. You should upload this as a separate file labeled 'Revised Manuscript with Track Changes'.
- An unmarked version of your revised paper without tracked changes. You should upload this as a separate file labeled 'Manuscript'.

If you would like to make changes to your financial disclosure, please include your updated statement in your cover letter. Guidelines for resubmitting your figure files are available below the reviewer comments at the end of this letter.

If applicable, we recommend that you deposit your laboratory protocols in protocols.io to enhance the reproducibility of your results. Protocols.io assigns your protocol its own identifier (DOI) so that it can be cited independently in the future. For instructions see: https://journals.plos.org/plosone/s/submission-guidelines#loc-laboratory-protocols. Additionally, PLOS ONE offers an option for publishing peer-reviewed Lab Protocol articles, which describe protocols hosted on protocols.io. Read more information on sharing protocols at https://plos.org/protocols?utm_medium=editorial-email&utm_source=authorletters&utm_campaign=protocols.

As the corresponding author, your ORCID iD is verified in the submission system and will appear in the published article. PLOS supports the use of ORCID, and we encourage all coauthors to register for an ORCID iD and use it as well. Please encourage your coauthors to verify their ORCID iD within the submission system before final acceptance, as unverified ORCID iDs will not appear in the published article. *Only*  the individual author can complete the verification step; PLOS staff *cannot*  verify ORCID iDs on behalf of authors.

We look forward to receiving your revised manuscript.

Kind regards,

Onder Aydemir

Academic Editor

PLOS One

**Journal Requirements:**

When submitting your revision, we need you to address these additional requirements.

1. Please ensure that your manuscript meets PLOS ONE's style requirements, including those for file naming. The PLOS ONE style templates can be found at

https://journals.plos.org/plosone/s/file?id=wjVg/PLOSOne_formatting_sample_main_body.pdf and

https://journals.plos.org/plosone/s/file?id=ba62/PLOSOne_formatting_sample_title_authors_affiliations.pdf

2. Please note that PLOS One has specific guidelines on code sharing for submissions in which author-generated code underpins the findings in the manuscript. In these cases, we expect all author-generated code to be made available without restrictions upon publication of the work. Please review our guidelines at https://journals.plos.org/plosone/s/materials-and-software-sharing#loc-sharing-code and ensure that your code is shared in a way that follows best practice and facilitates reproducibility and reuse.

3. If the reviewer comments include a recommendation to cite specific previously published works, please review and evaluate these publications to determine whether they are relevant and should be cited. There is no requirement to cite these works unless the editor has indicated otherwise.

**Additional Editor Comments:**

Dear Author,

The reviewers’ comments should be addressed carefully and appropriately in the revised version of your manuscript. Based on the reviewers’ evaluations, a decision of Major Revision has been made. Please submit a revised manuscript together with a detailed response letter explaining how each reviewer comment has been addressed.

We look forward to receiving your revised submission.

Best regards,

Dr. Önder Aydemir

Reviewer Comments:

Reviewer 1#

The core idea is sound, the pipeline is clearly described, and the use of a publicly available dataset is commendable.

The following comments are intended to help the authors bring the manuscript to a publishable state:

The most important issue to address is the artifact confound. The electrodes most frequently identified as exhibiting task-induced deviations, namely Fp1, Fp2, T3, and T5, are precisely those most susceptible to ocular, muscular, and movement artifacts during an active cognitive task compared to eyes-closed rest. The authors acknowledge in the pipeline description that detected outliers may reflect either genuine task-related neural activity or artifacts, but this distinction is never pursued in the analysis. The neurophysiological interpretations offered throughout the results and discussion therefore rest on an unverified assumption. The authors should include an explicit discussion of how artifact contamination may contribute to the observed electrode distribution, and ideally should apply an independent artifact characterization step, such as comparing deviant segment topographies against known artifact spatial signatures, to strengthen confidence in the neural interpretation.

The statistical analysis requires attention in two areas. First, the large number of simultaneous tests conducted across metrics, electrodes, frequency bands, and subjects means that the reported p-values are likely inflated due to multiple comparisons. A correction procedure such as Benjamini-Hochberg false discovery rate control should be applied and the results re-reported accordingly. Second, the MAPE metric produces a mean of 420.63 with a standard deviation exceeding 11,000 and a maximum above one million during the arithmetic task, while the median remains below one. Describing this distribution using means and standard deviations is statistically inappropriate. The authors should either apply a log transformation before reporting summary statistics for this metric or replace it with robust alternatives such as median and interquartile range throughout.

The synthetic validation in Section 2.2.8 uses sinusoidal perturbations as the abnormal class. These produce deviations that are structurally very different from the subtle, broadband reconstruction errors expected from genuine task-induced EEG changes, and the near-perfect detection rates achieved by IF and LOF on synthetic data do not translate into meaningful evidence about the method's performance on real task segments. The authors should explicitly acknowledge this limitation and clarify what the synthetic validation is and is not intended to demonstrate.

The OCSVM normal-sample accuracy of 64% with a standard deviation of 38% is poor and highly variable across subjects, indicating near-random classification for a substantial subset of participants. The subject-level deviant rate ranging from 1% to 86% compounds this concern. The authors should investigate whether subjects with high deviant rates correspond to those with poor reconstruction quality or limited training data, and should discuss what this variability implies for the reliability of the electrode-level conclusions.

Finally, the manuscript contains two instances of duplicated words, specifically "suggesting suggesting" and "spatially spatially," and should be carefully proofread before resubmission.

Reviewer 2#

Summary: This study presents a 1-second segment-based, self-supervised framework that utilizes a hierarchical variational autoencoder (hvEEGNet) and a One-Class SVM (OCSVM) to detect EEG signal deviations (anomalies) during mental arithmetic tasks. The study's aim to capture transient cortical dynamics by examining signals in short-term segments rather than analyzing them globally is scientifically valuable. However, there are very serious structural issues regarding methodological originality, the clear contribution to literature, the validity of the validation using synthetic data, and the reliability of the physiological interpretations that must absolutely be addressed before acceptance for publication.

Major Comments:

• Weak Contribution to the Literature and Lack of Methodological Originality: The authors use the "hvEEGNet" architecture for signal reconstruction, and the text explicitly states that this architecture was already developed by Cisotto et al. (Ref [24]). The primary methodological contribution of this study appears to consist merely of adding metrics such as DTW, MAPE, PRD, STFTL, and SNR to the outputs of an existing model to perform anomaly detection with an OCSVM algorithm. The authors must go beyond simply testing an existing model on a new dataset; they must provide strong theoretical proof of why their methodology is structurally superior or more innovative than previous approaches (e.g., those using only kNN or simple thresholding).

• Circularity in Validation with Synthetic Data: In Section 2.2.8, the authors state that they generated synthetic data using the already trained hvEEGNet model to validate the anomaly detection algorithms (OCSVM, IF, LOF). Validating an anomaly detection algorithm tested on synthetic data generated by a VAE (hvEEGNet) itself, using its own reconstruction errors, is methodologically flawed (a circular logic error). The VAE will transfer its own learned biases to the synthetic data. This does not scientifically prove that the model can capture real-world physiological anomalies; it merely shows that the model validates itself.

• Physiological Interpretations and the Possibility of Artifacts (Noise): The study finds that statistical deviations occur more frequently in peripheral electrodes such as Fp1, Fp2, T3, and T5. It is well known in the literature that Fp1 and Fp2 are highly susceptible to blinking/eye movements (EOG), while T3 and T5 are highly susceptible to jaw or neck muscle tension (EMG). Although the authors claim the dataset used is "artifact-free," involuntary muscle contractions (e.g., frowning) can occur during tasks requiring high cognitive effort, such as mental arithmetic. The authors must discuss in detail whether these detected deviations are truly complex cognitive processes or physiological artifacts (muscle tension) caused by increased mental effort.

• Correlation of Metrics and Dimensionality (Curse of Dimensionality): A vector of 6 different metrics (DTW, NC, MAPE, PRD, STFTL, SNR) is provided as input to the OCSVM for anomaly detection. Error metrics such as MAPE and PRD generally show a very high correlation with each other. This situation can lead to unnecessary dimensionality in the model. The authors should present the correlation matrix between these 6 features and increase the robustness of the model by eliminating redundant features.

Section-by-Section Comments:

• Introduction: The introduction states that previous studies found contradictory results on the same dataset. However, it is not fully justified why the proposed "1-second segment" approach will physiologically resolve these contradictions in the literature. Window size is very critical in signal processing; why were exactly 1-second windows chosen instead of 2 seconds or 0.5 seconds? The signal processing or physiological foundations of this choice must be explained.

• Methods: How the hyperparameters of the One-Class Learning algorithms (OCSVM, IF, LOF) (e.g., kernel type or nu parameter for OCSVM, number of trees for IF) were selected and optimized is left missing in the methodology. The hyperparameter optimization process must be detailed.

• Results: In Section 3.4, the spatial differences (electrode-based) between "good quality counters" and "poor quality counters" are discussed. However, it is not specified in the Methods section exactly what criteria were used to define these subject groups in the dataset, how they were separated, and how many patients are in each group.

• Conclusion: The conclusion section should concretely state how the findings align with (or resolve) previous contradictory findings in the literature. Furthermore, the inferences regarding the practical clinical or neurological benefits of the article remain weak and should be strengthened.

Reviewer 3#

This paper proposes a self-supervised framework for segment-based anomaly detection using deep learning to identify EEG signal outliers during mental arithmetic. 1) The reconstruction error metrics across segments from all subjects reported in table 2 show that outliers do exist during the arithmetic task. The experimental results support the manuscript’s conclusions and the manuscript is technically sound. 2) The statistical analysis based on non-parametric Mann–Whitney U test suggests that for five of all six metrics, reconstruction errors were significantly higher during the arithmetic task than in the resting state with p < 0.001. The statistical analysis was performed appropriately and rigorously. 3) The data used in study is the EEG dataset of Mental Arithmetic Task Performance, that is publicly available. 4) The article is well organized and written in standard English.

The reviewer has one concern: where are the Figures 4 and 5?

Reviewer 4#

The article presents work on a matter of high interest on the EEG comunity.

Before discussing the technical side, an important point to address is the length of the article. It is excesive for the topic and the information produced. Several parts of the article re-write the same ideas with different formats without introducing any new information to the reader. I would request the authors to thighten the text, introduce every idea once in the correct section and refer to the sections when required. Also, the authors should improve the use of references, not only as support of the considerations and results in this article but also to dedicate the space to the important new findings and not information that is well known or easily referenced such as the error metrics.

Some more specific coments below:

- Introduction: Ensure to define the achronims the first time they are used, example SVM, used and defined on the second instance, not the first. others like NC and PRD must be corrected as well.

- Introduction: the authors describe the use of 1sec segments. What lead to this selection? Explain clearly how shortter and longer segments would affect the results, and how the authors make the results not sensitive to the segment length. A proper study on this topic must be presented.

- Metholodogy: The authors describe the process including that "the signal is divided into non-overlapping...", what are the effect of data bining on the signal processing results? what are the effects of overlapping? what are the edge effects specially in frequency domain?

- Signal segmentation: What is the correlation between outlier detection on different channels? Although the assumption is that there is no influence of deviations on one channel onto other chanels, present the prove that this thesis is correct. In most life signals detection there is some level of influece between channels and the results are presented under the limitations of these influences and thresholds are presented, not mathematically but physically. Since the authors ensure that the multichannel reconstruction is appropriate with zero cross-channel influence, the data showing this is true is necessary, otherwise clearly present the limitations.

- Reconstruction metrics: Unnecessary long. The text must be shortened and optimized. the table is useful, but refer the reader to the adequate references and use equations that absolutely necessary for the understanding of the work.

- Experimental setup and design rationale: Many ideas are just a repetition of prior sections. Consider a deep review of this section, some information, specially from the first few paragraphs belong to the introduction of the paper.

- SEction 2.2.7 Present supporting information regarding the window size, what would be the minimum N value that can be used?

- Section 2.2.7 Overlapping: find the correct section for the discussion and ensure to present mathematical prove on why this should be avoided or use adquate references.

- Section 2.2.8 las paragraph: Repetition of the methodology described in previous section. Same with paragraph 1 page 14.

The motivation should be addressed in the introduction of the paper, not in a later section, unless there is a direct influence in the results that the reader has to be reminded of, which is not happening in page 14.

Section 2.2.8

- Although sinusoidal signals are a good start of the perturbation study, even with the use of random frequency components, the observations can be quite limited. I strongly encourage the authors to include other type of signals such as wavelets, boadband gaussian noise, modified sinusoidal oscillation with gaussian tappering , non-stationary colored noise, frequency bursts (specially high freq), etc. This would ensure the results are sound and the methodology is tested against challenging and more realistic perturbations

Section 3.1. The authors describe how different metrics were less consistent across subjects, either the authors assess the meaning of this conclussion or eliminate the non-consistent metrics from the analysis, with suporting work.Some questions that could guide the process with regards to the observations: Are the metrics used incorrect for the study? or do the observations determine that other underlying effects may be affecting the results and the current metrics are insufient and more work is necessary?

Table 2, could better be a figure that sompare the resting state with the arithmetic task, although the information is there, the task of drawing a full picture of the results is left to the reader.

Standard deviation, results are presented numerically, the meaning of these results is not addressed.

This section lacks a strong conclussion, are the metrics and methodology used able to discriminate between resting state and brain activation due to arithmetic tasks? Also are the metrics used in this study enogh to fulfill the rquirements of discrimitation and identification of abnormal data?

Section 3.5 is interesting, but the results seem inconclusive, is the aim of the authors to tie these resulst with those of the previous sections? If that is the intent, a review of the text and data presentation should be considered.

Reviewer 5#

The article presents work on a matter of high interest on the EEG comunity.

Before discussing the technical side, an important point to address is the length of the article. It is excesive for the topic and the information produced. Several parts of the article re-write the same ideas with different formats without introducing any new information to the reader. I would request the authors to thighten the text, introduce every idea once in the correct section and refer to the sections when required. Also, the authors should improve the use of references, not only as support of the considerations and results in this article but also to dedicate the space to the important new findings and not information that is well known or easily referenced such as the error metrics.

Some more specific coments below:

- Introduction: Ensure to define the achronims the first time they are used, example SVM, used and defined on the second instance, not the first. others like NC and PRD must be corrected as well.

- Introduction: the authors describe the use of 1sec segments. What lead to this selection? Explain clearly how shortter and longer segments would affect the results, and how the authors make the results not sensitive to the segment length. A proper study on this topic must be presented.

- Metholodogy: The authors describe the process including that "the signal is divided into non-overlapping...", what are the effect of data bining on the signal processing results? what are the effects of overlapping? what are the edge effects specially in frequency domain?

- Signal segmentation: What is the correlation between outlier detection on different channels? Although the assumption is that there is no influence of deviations on one channel onto other chanels, present the prove that this thesis is correct. In most life signals detection there is some level of influece between channels and the results are presented under the limitations of these influences and thresholds are presented, not mathematically but physically. Since the authors ensure that the multichannel reconstruction is appropriate with zero cross-channel influence, the data showing this is true is necessary, otherwise clearly present the limitations.

- Reconstruction metrics: Unnecessary long. The text must be shortened and optimized. the table is useful, but refer the reader to the adequate references and use equations that absolutely necessary for the understanding of the work.

- Experimental setup and design rationale: Many ideas are just a repetition of prior sections. Consider a deep review of this section, some information, specially from the first few paragraphs belong to the introduction of the paper.

- SEction 2.2.7 Present supporting information regarding the window size, what would be the minimum N value that can be used?

- Section 2.2.7 Overlapping: find the correct section for the discussion and ensure to present mathematical prove on why this should be avoided or use adquate references.

- Section 2.2.8 las paragraph: Repetition of the methodology described in previous section. Same with paragraph 1 page 14.

The motivation should be addressed in the introduction of the paper, not in a later section, unless there is a direct influence in the results that the reader has to be reminded of, which is not happening in page 14.

Section 2.2.8

- Although sinusoidal signals are a good start of the perturbation study, even with the use of random frequency components, the observations can be quite limited. I strongly encourage the authors to include other type of signals such as wavelets, boadband gaussian noise, modified sinusoidal oscillation with gaussian tappering , non-stationary colored noise, frequency bursts (specially high freq), etc. This would ensure the results are sound and the methodology is tested against challenging and more realistic perturbations

Section 3.1. The authors describe how different metrics were less consistent across subjects, either the authors assess the meaning of this conclussion or eliminate the non-consistent metrics from the analysis, with suporting work.Some questions that could guide the process with regards to the observations: Are the metrics used incorrect for the study? or do the observations determine that other underlying effects may be affecting the results and the current metrics are insufient and more work is necessary?

Table 2, could better be a figure that sompare the resting state with the arithmetic task, although the information is there, the task of drawing a full picture of the results is left to the reader.

Standard deviation, results are presented numerically, the meaning of these results is not addressed.

This section lacks a strong conclussion, are the metrics and methodology used able to discriminate between resting state and brain activation due to arithmetic tasks? Also are the metrics used in this study enogh to fulfill the rquirements of discrimitation and identification of abnormal data?

Section 3.5 is interesting, but the results seem inconclusive, is the aim of the authors to tie these resulst with those of the previous sections? If that is the intent, a review of the text and data presentation should be considered.

Reviewer 6#

Reviewer comments

PONE-D-26-16141, “A self-supervised reconstruction framework for segment-level detection of taskinduced deviations in multi-channel EEG” Abstract:

1. Please declare the electroencephalogram first before using the ”EEG” abbreviation in the second line.

2. Please remove the abbreviation ”hvEEGNet” because it is not used in the abstract.

3. Please remove the abbreviation ”DTW, STFT, SNR” because they are not used in the abstract.

4. Please review the tense used in describing the experimental methods and results, it is preferred to use past

tense.

Introduction section:

1. Please use (i.e. BA 46) instead of (BA 46). (line 17)

2. Please reorder the cited references to be [3] [10] instead of [10] [3]. (line 22)

3. Please declare ”SVM” first before using the abbreviation. (line 40)

4. Please declare both ”LDA and kNN” before using them. (line 51)

5. In line 74, what do you mean by ”...,which are not available for this dataset”, which dataset are you

referring to?

6. Please declare ”hvEEGNet” first before using it. (line 88)

7. Please declare all ”NC, PRD, STFTL, and MAPE” first before using them. (line 94)

8. Please no need to use slanted font style in the paragraph started from line 108 to line 114.

9. Please remove the ”,” between segment-level and self supervised framework. (line 115)

10. In the last paragraph, all subsections of section 2 were mentioned except subsections 2.2.7 -2.2.9, please

state them.

Material and Methods section:

1. In section 2.1, please mention brief demographic information about the dataset used.

2. In section 2.2, have you calculated the training time for the proposed framework? if yes, please state the

computation capabilities used and the average time consumed.

3. (line 176) please use (i.e. electrode) instead of (electrode).

4. (line 181) please use (i.e. each value....) instead of (each value....). Do the same throughout the paper.

5. Please remove the last sentence in the caption of Figure 1, as it already explained in the context.

1

6. (line 211) please use (Eq. 1) instead of (1) to refer to Equation 1.

7. In subsection 2.2.2, please enumerate all the equations before Equation 4.

8. (line 437 and 439) please use (N = 200) instead of (N = 200).

9. In the statistical analysis subsection, Did you test the normality of data using Shapiro-Wilk test first before

using the Mann-Whitney U test? if, yes please state the test p-value.

Results and Discussion section:

1. In this section, it is recommended to use the past tense in reporting the findings.

2. (line 525) Please use ”60 s” instead of ”60 seconds”. Please use ”s” to refer to seconds throughout the

paper.

3. (line 535) What is the unit of measurement of DTW that reached approximately 13000.

4. (line 578) What is the unit of measurement of the standard deviation that increased frome 3.08 to 13.69

and similarly for the gamma band.

5. I can’t find figure 4 and 5 in the submitted file

[Note: HTML markup is below. Please do not edit.]

Reviewers' comments:

Reviewer's Responses to Questions

**Comments to the Author**

1. Is the manuscript technically sound, and do the data support the conclusions?

Reviewer #1: Partly

Reviewer #2: Yes

Reviewer #3: Yes

Reviewer #4: Partly

Reviewer #5: Yes

Reviewer #6: Partly

**********

2. Has the statistical analysis been performed appropriately and rigorously?

Reviewer #1: Yes

Reviewer #2: No

Reviewer #3: Yes

Reviewer #4: Yes

Reviewer #5: Yes

Reviewer #6: Yes

**********

3. Have the authors made all data underlying the findings in their manuscript fully available?

The PLOS Data policy

Reviewer #1: Yes

Reviewer #2: Yes

Reviewer #3: Yes

Reviewer #4: Yes

Reviewer #5: Yes

Reviewer #6: Yes

**********

4. Is the manuscript presented in an intelligible fashion and written in standard English?

Reviewer #1: Yes

Reviewer #2: Yes

Reviewer #3: Yes

Reviewer #4: Yes

Reviewer #5: No

Reviewer #6: Yes

**********

Reviewer #1: The core idea is sound, the pipeline is clearly described, and the use of a publicly available dataset is commendable.

The following comments are intended to help the authors bring the manuscript to a publishable state:

The most important issue to address is the artifact confound. The electrodes most frequently identified as exhibiting task-induced deviations, namely Fp1, Fp2, T3, and T5, are precisely those most susceptible to ocular, muscular, and movement artifacts during an active cognitive task compared to eyes-closed rest. The authors acknowledge in the pipeline description that detected outliers may reflect either genuine task-related neural activity or artifacts, but this distinction is never pursued in the analysis. The neurophysiological interpretations offered throughout the results and discussion therefore rest on an unverified assumption. The authors should include an explicit discussion of how artifact contamination may contribute to the observed electrode distribution, and ideally should apply an independent artifact characterization step, such as comparing deviant segment topographies against known artifact spatial signatures, to strengthen confidence in the neural interpretation.

The statistical analysis requires attention in two areas. First, the large number of simultaneous tests conducted across metrics, electrodes, frequency bands, and subjects means that the reported p-values are likely inflated due to multiple comparisons. A correction procedure such as Benjamini-Hochberg false discovery rate control should be applied and the results re-reported accordingly. Second, the MAPE metric produces a mean of 420.63 with a standard deviation exceeding 11,000 and a maximum above one million during the arithmetic task, while the median remains below one. Describing this distribution using means and standard deviations is statistically inappropriate. The authors should either apply a log transformation before reporting summary statistics for this metric or replace it with robust alternatives such as median and interquartile range throughout.

The synthetic validation in Section 2.2.8 uses sinusoidal perturbations as the abnormal class. These produce deviations that are structurally very different from the subtle, broadband reconstruction errors expected from genuine task-induced EEG changes, and the near-perfect detection rates achieved by IF and LOF on synthetic data do not translate into meaningful evidence about the method's performance on real task segments. The authors should explicitly acknowledge this limitation and clarify what the synthetic validation is and is not intended to demonstrate.

The OCSVM normal-sample accuracy of 64% with a standard deviation of 38% is poor and highly variable across subjects, indicating near-random classification for a substantial subset of participants. The subject-level deviant rate ranging from 1% to 86% compounds this concern. The authors should investigate whether subjects with high deviant rates correspond to those with poor reconstruction quality or limited training data, and should discuss what this variability implies for the reliability of the electrode-level conclusions.

Finally, the manuscript contains two instances of duplicated words, specifically "suggesting suggesting" and "spatially spatially," and should be carefully proofread before resubmission.

Reviewer #2: Recommendation: Major Revision

Summary: This study presents a 1-second segment-based, self-supervised framework that utilizes a hierarchical variational autoencoder (hvEEGNet) and a One-Class SVM (OCSVM) to detect EEG signal deviations (anomalies) during mental arithmetic tasks. The study's aim to capture transient cortical dynamics by examining signals in short-term segments rather than analyzing them globally is scientifically valuable. However, there are very serious structural issues regarding methodological originality, the clear contribution to literature, the validity of the validation using synthetic data, and the reliability of the physiological interpretations that must absolutely be addressed before acceptance for publication.

Major Comments:

• Weak Contribution to the Literature and Lack of Methodological Originality: The authors use the "hvEEGNet" architecture for signal reconstruction, and the text explicitly states that this architecture was already developed by Cisotto et al. (Ref [24]). The primary methodological contribution of this study appears to consist merely of adding metrics such as DTW, MAPE, PRD, STFTL, and SNR to the outputs of an existing model to perform anomaly detection with an OCSVM algorithm. The authors must go beyond simply testing an existing model on a new dataset; they must provide strong theoretical proof of why their methodology is structurally superior or more innovative than previous approaches (e.g., those using only kNN or simple thresholding).

• Circularity in Validation with Synthetic Data: In Section 2.2.8, the authors state that they generated synthetic data using the already trained hvEEGNet model to validate the anomaly detection algorithms (OCSVM, IF, LOF). Validating an anomaly detection algorithm tested on synthetic data generated by a VAE (hvEEGNet) itself, using its own reconstruction errors, is methodologically flawed (a circular logic error). The VAE will transfer its own learned biases to the synthetic data. This does not scientifically prove that the model can capture real-world physiological anomalies; it merely shows that the model validates itself.

• Physiological Interpretations and the Possibility of Artifacts (Noise): The study finds that statistical deviations occur more frequently in peripheral electrodes such as Fp1, Fp2, T3, and T5. It is well known in the literature that Fp1 and Fp2 are highly susceptible to blinking/eye movements (EOG), while T3 and T5 are highly susceptible to jaw or neck muscle tension (EMG). Although the authors claim the dataset used is "artifact-free," involuntary muscle contractions (e.g., frowning) can occur during tasks requiring high cognitive effort, such as mental arithmetic. The authors must discuss in detail whether these detected deviations are truly complex cognitive processes or physiological artifacts (muscle tension) caused by increased mental effort.

• Correlation of Metrics and Dimensionality (Curse of Dimensionality): A vector of 6 different metrics (DTW, NC, MAPE, PRD, STFTL, SNR) is provided as input to the OCSVM for anomaly detection. Error metrics such as MAPE and PRD generally show a very high correlation with each other. This situation can lead to unnecessary dimensionality in the model. The authors should present the correlation matrix between these 6 features and increase the robustness of the model by eliminating redundant features.

Section-by-Section Comments:

• Introduction: The introduction states that previous studies found contradictory results on the same dataset. However, it is not fully justified why the proposed "1-second segment" approach will physiologically resolve these contradictions in the literature. Window size is very critical in signal processing; why were exactly 1-second windows chosen instead of 2 seconds or 0.5 seconds? The signal processing or physiological foundations of this choice must be explained.

• Methods: How the hyperparameters of the One-Class Learning algorithms (OCSVM, IF, LOF) (e.g., kernel type or nu parameter for OCSVM, number of trees for IF) were selected and optimized is left missing in the methodology. The hyperparameter optimization process must be detailed.

• Results: In Section 3.4, the spatial differences (electrode-based) between "good quality counters" and "poor quality counters" are discussed. However, it is not specified in the Methods section exactly what criteria were used to define these subject groups in the dataset, how they were separated, and how many patients are in each group.

• Conclusion: The conclusion section should concretely state how the findings align with (or resolve) previous contradictory findings in the literature. Furthermore, the inferences regarding the practical clinical or neurological benefits of the article remain weak and should be strengthened.

Reviewer #3: This paper proposes a self-supervised framework for segment-based anomaly detection using deep learning to identify EEG signal outliers during mental arithmetic. 1) The reconstruction error metrics across segments from all subjects reported in table 2 show that outliers do exist during the arithmetic task. The experimental results support the manuscript’s conclusions and the manuscript is technically sound. 2) The statistical analysis based on non-parametric Mann–Whitney U test suggests that for five of all six metrics, reconstruction errors were significantly higher during the arithmetic task than in the resting state with p < 0.001. The statistical analysis was performed appropriately and rigorously. 3) The data used in study is the EEG dataset of Mental Arithmetic Task Performance, that is publicly available. 4) The article is well organized and written in standard English.

The reviewer has one concern: where are the Figures 4 and 5?

Reviewer #4: The article presents work on a matter of high interest on the EEG comunity.

Before discussing the technical side, an important point to address is the length of the article. It is excesive for the topic and the information produced. Several parts of the article re-write the same ideas with different formats without introducing any new information to the reader. I would request the authors to thighten the text, introduce every idea once in the correct section and refer to the sections when required. Also, the authors should improve the use of references, not only as support of the considerations and results in this article but also to dedicate the space to the important new findings and not information that is well known or easily referenced such as the error metrics.

Some more specific coments below:

- Introduction: Ensure to define the achronims the first time they are used, example SVM, used and defined on the second instance, not the first. others like NC and PRD must be corrected as well.

- Introduction: the authors describe the use of 1sec segments. What lead to this selection? Explain clearly how shortter and longer segments would affect the results, and how the authors make the results not sensitive to the segment length. A proper study on this topic must be presented.

- Metholodogy: The authors describe the process including that "the signal is divided into non-overlapping...", what are the effect of data bining on the signal processing results? what are the effects of overlapping? what are the edge effects specially in frequency domain?

- Signal segmentation: What is the correlation between outlier detection on different channels? Although the assumption is that there is no influence of deviations on one channel onto other chanels, present the prove that this thesis is correct. In most life signals detection there is some level of influece between channels and the results are presented under the limitations of these influences and thresholds are presented, not mathematically but physically. Since the authors ensure that the multichannel reconstruction is appropriate with zero cross-channel influence, the data showing this is true is necessary, otherwise clearly present the limitations.

- Reconstruction metrics: Unnecessary long. The text must be shortened and optimized. the table is useful, but refer the reader to the adequate references and use equations that absolutely necessary for the understanding of the work.

- Experimental setup and design rationale: Many ideas are just a repetition of prior sections. Consider a deep review of this section, some information, specially from the first few paragraphs belong to the introduction of the paper.

- SEction 2.2.7 Present supporting information regarding the window size, what would be the minimum N value that can be used?

- Section 2.2.7 Overlapping: find the correct section for the discussion and ensure to present mathematical prove on why this should be avoided or use adquate references.

- Section 2.2.8 las paragraph: Repetition of the methodology described in previous section. Same with paragraph 1 page 14.

The motivation should be addressed in the introduction of the paper, not in a later section, unless there is a direct influence in the results that the reader has to be reminded of, which is not happening in page 14.

Section 2.2.8

- Although sinusoidal signals are a good start of the perturbation study, even with the use of random frequency components, the observations can be quite limited. I strongly encourage the authors to include other type of signals such as wavelets, boadband gaussian noise, modified sinusoidal oscillation with gaussian tappering , non-stationary colored noise, frequency bursts (specially high freq), etc. This would ensure the results are sound and the methodology is tested against challenging and more realistic perturbations

Section 3.1. The authors describe how different metrics were less consistent across subjects, either the authors assess the meaning of this conclussion or eliminate the non-consistent metrics from the analysis, with suporting work.Some questions that could guide the process with regards to the observations: Are the metrics used incorrect for the study? or do the observations determine that other underlying effects may be affecting the results and the current metrics are insufient and more work is necessary?

Table 2, could better be a figure that sompare the resting state with the arithmetic task, although the information is there, the task of drawing a full picture of the results is left to the reader.

Standard deviation, results are presented numerically, the meaning of these results is not addressed.

This section lacks a strong conclussion, are the metrics and methodology used able to discriminate between resting state and brain activation due to arithmetic tasks? Also are the metrics used in this study enogh to fulfill the rquirements of discrimitation and identification of abnormal data?

Section 3.5 is interesting, but the results seem inconclusive, is the aim of the authors to tie these resulst with those of the previous sections? If that is the intent, a review of the text and data presentation should be considered.

Reviewer #5: The manuscript presents a self-supervised framework based on hvEEGNet for detecting segment-level deviations in EEG recordings during mental arithmetic tasks. The topic is timely and relevant, particularly because the study attempts to address inconsistencies in prior analyses of the same EEG dataset by focusing on transient segment-level deviations instead of global EEG characteristics. The integration of reconstruction metrics (DTW, STFTL, SNR, etc.) with one-class anomaly detection is methodologically interesting and potentially useful for EEG analysis where labelled datasets are unavailable. The manuscript is technically ambitious and generally well organized. However, there are several methodological, statistical, interpretational, and presentation-related weaknesses that substantially limit the strength of the conclusions.

1. The authors are suggested to include a artifact robustness analysis to demonstrate detected deviations persist aftrer artifact rejection procedure.

2. The authors should elaborate the novelty concisely and justy the work is not a stacked piece of earlier works.

3. The concept of “outlier,” “anomaly,” and “task-induced deviation” is used somewhat interchangeably throughout the manuscript. The authors should be aware and improve the technical clarity of the manuscript.

4. The English is generally understandable but occasionally awkward and overly formal. Several sections of the manuscript would benefit from professional language editing.

5. The manuscript lacks comparison with some baseline methods or prior established works. Without these comparisons, it is difficult to quantify the true advantage of the proposed framework. The authors should try simpler reconstruction methods at least.

Reviewer #6: The paper presents a self-supervised framework for segment-based detection of task-induced deviations in multi-channel EEG. First, I would like to thank the authors for effort they have done to finish this work. However, I have some concerns and comments stated in the attached file.

**********

what does this mean?). If published, this will include your full peer review and any attached files.

If you choose “no”, your identity will remain anonymous but your review may still be made public.

**Do you want your identity to be public for this peer review?**  For information about this choice, including consent withdrawal, please see our Privacy Policy

Reviewer #1: **Yes:**  Md Abubakkar

Reviewer #2: No

Reviewer #3: No

Reviewer #4: No

Reviewer #5: **Yes:**  Khondakar Ashik Shahriar

Reviewer #6: No

**********

[NOTE: If reviewer comments were submitted as an attachment file, they will be attached to this email and accessible via the submission site. Please log into your account, locate the manuscript record, and check for the action link "View Attachments". If this link does not appear, there are no attachment files.]

To ensure your figures meet our technical requirements, please review our figure guidelines: https://journals.plos.org/plosone/s/figures

You may also use PLOS’s free figure tool, NAAS, to help you prepare publication quality figures: https://journals.plos.org/plosone/s/figures#loc-tools-for-figure-preparation.

NAAS will assess whether your figures meet our technical requirements by comparing each figure against our figure specifications.

Attachment Submitted filename: Reviewer Comments.pdf

Attachment Submitted filename: Reviewer_response.pdf

# Author comment: Author response to Decision Letter 1

**DOI:** 10.1371/journal.pone.0361586.r002

6 Aug 2026

Dear Editor and reviewers,

We thank the editor and the six reviewers for their careful and thoughtful examination of our

paper. We appreciate the time and effort they have put into their reviews.

We have provided a response letter with answers to each and every remarks by the six reviewers (please see uploaded files).

The main changes made to the manuscript are shown in red.

We also proofread the whole text and made many changes (not shown in red) to correct some typos and

improve the writing style for better readability.

Attachment Submitted filename: Responses_to_reviewers.pdf

# Peer review: Decision Letter 1

**DOI:** 10.1371/journal.pone.0361586.r003

**Authors:** Onder Aydemir

24 Aug 2026

Dear Dr. Batatia,

Thank you for submitting your manuscript to PLOS One. After careful consideration, we feel that it has merit but does not fully meet PLOS One’s publication criteria as it currently stands. Therefore, we invite you to submit a revised version of the manuscript that addresses the points raised during the review process.

Please submit your revised manuscript by Oct 08 2026 11:59PM. If you will need more time than this to complete your revisions, please reply to this message or contact the journal office at plosone@plos.org. When you're ready to submit your revision, log on to https://www.editorialmanager.com/pone/ and select the 'Submissions Needing Revision' folder to locate your manuscript file.

- A letter that responds to each point raised by the academic editor and reviewer(s). You should upload this letter as a separate file labeled 'Response to Reviewers'.
- A marked-up copy of your manuscript that highlights changes made to the original version. You should upload this as a separate file labeled 'Revised Manuscript with Track Changes'.
- An unmarked version of your revised paper without tracked changes. You should upload this as a separate file labeled 'Manuscript'.

If you would like to make changes to your financial disclosure, please include your updated statement in your cover letter. Guidelines for resubmitting your figure files are available below the reviewer comments at the end of this letter.

If applicable, we recommend that you deposit your laboratory protocols in protocols.io to enhance the reproducibility of your results. Protocols.io assigns your protocol its own identifier (DOI) so that it can be cited independently in the future. For instructions see: https://journals.plos.org/plosone/s/submission-guidelines#loc-laboratory-protocols. Additionally, PLOS One offers an option for publishing peer-reviewed Lab Protocol articles, which describe protocols hosted on protocols.io. Read more information on sharing protocols at https://plos.org/protocols?utm_medium=editorial-email&utm_source=authorletters&utm_campaign=protocols.

As the corresponding author, your ORCID iD is verified in the submission system and will appear in the published article. PLOS supports the use of ORCID, and we encourage all coauthors to register for an ORCID iD and use it as well. Please encourage your coauthors to verify their ORCID iD within the submission system before final acceptance, as unverified ORCID iDs will not appear in the published article. *Only*  the individual author can complete the verification step; PLOS staff *cannot*  verify ORCID iDs on behalf of authors.

We look forward to receiving your revised manuscript.

Kind regards,

Onder Aydemir

Academic Editor

PLOS One

Journal Requirements:

If the reviewer comments include a recommendation to cite specific previously published works, please review and evaluate these publications to determine whether they are relevant and should be cited. There is no requirement to cite these works unless the editor has indicated otherwise.

[Note: HTML markup is below. Please do not edit.]

Reviewers' comments:

Reviewer's Responses to Questions

**Comments to the Author**

Reviewer #1: (No Response)

Reviewer #2: All comments have been addressed

Reviewer #5: All comments have been addressed

**********

2. Is the manuscript technically sound, and do the data support the conclusions?

Reviewer #1: Partly

Reviewer #2: Yes

Reviewer #5: Yes

**********

3. Has the statistical analysis been performed appropriately and rigorously?

Reviewer #1: Yes

Reviewer #2: Yes

Reviewer #5: Yes

**********

4. Have the authors made all data underlying the findings in their manuscript fully available?

The PLOS Data policy

Reviewer #1: Yes

Reviewer #2: Yes

Reviewer #5: Yes

**********

5. Is the manuscript presented in an intelligible fashion and written in standard English?

Reviewer #1: Yes

Reviewer #2: Yes

Reviewer #5: Yes

**********

Reviewer #1: I reviewed this revision against the previous round of comments. The authors have engaged seriously with the six reviewers, and the manuscript has improved in several important respects: the terminology around anomaly, outlier and deviation is now defined explicitly, the contribution relative to Cisotto et al. is stated clearly rather than left implicit, FDR correction has been applied, the artifact heuristic and the permutation test of cross-channel dependence are useful additions, and the counter group definitions and demographics are now given. The scoping of the synthetic validation, so that it claims only to show that the detectors reject samples outside the support of the resting distribution, is an honest and appropriate retreat from the earlier framing. I think the paper is close, but I do not think it is acceptable in its present form, mainly because of one missing control and a set of internal contradictions that a reader will notice.

The missing control is the one I consider essential. The OCSVM is fitted with a fixed nu of 0.1, and Table 3 reports normal sample accuracy of 64% with a standard deviation of 38%, which implies a false positive rate on non deviant data of roughly a third, with enormous subject to subject spread. The reported outcome is that a mean of 30% of task segments are flagged as deviant, with a range from 1% to 86%. As presented, the headline deviant rate is not distinguishable from the detector's own false alarm rate. The fix is straightforward and does not require new data: hold out a portion of each subject's resting recording, exclude it from OCSVM fitting, apply the trained detector to it, and report the resulting deviant rate alongside the task rate, with a paired test across subjects. If the task rate is significantly higher than the rest rate, the central claim is secure and the paper is much stronger for it. If it is not, that needs to be known before publication. The correlation analyses added in response to Remark 1.4 address a different question and do not substitute for this.

There is also a direct contradiction between the abstract and the methods. The abstract still states that outliers are detected using an unsupervised method with automated hyper parameter tuning, while Section 2.2.5 and the response to Remark 2.6 state plainly that no systematic hyperparameter search was performed and that scikit-learn defaults were used throughout. The abstract must be corrected.

Two results statements also contradict themselves. In Section 3.1, the text reports significantly elevated PSD area in poorly reconstructed segments in the delta, theta and beta bands with no significant difference in alpha, and then the summary paragraph a few lines later says that poorly reconstructed segments exhibit attenuated activity in the delta, theta and alpha bands. One of these is wrong. Separately, in Section 3.3 the standard deviation results show significant differences in 86%, 75%, 72%, 72% and 83% of subjects for delta through gamma, yet the text concludes that deviations predominantly affect lower frequency rhythms. Gamma at 83% is essentially as high as delta, and this matters because the artifact rebuttal in the same section rests on the claim that deviations concentrate in delta and theta rather than in the beta and gamma range associated with myogenic activity. That argument needs to be reconciled with the authors' own numbers or softened accordingly.

Two smaller points on consistency between the response letter and the manuscript. The response to Remark 5.5 states that the framework was evaluated by comparison with simple baseline reconstruction methods, but I could not find any such comparison in the revised manuscript; the kNN thresholding approach is discussed narratively in Section 2.2.5 but never run. Either add the comparison or remove the claim from the response. Likewise, Section 2.1 still describes the dataset as artifact free, which sits awkwardly beside the extended discussion in Section 3.3 of ICA cleaning, residual contamination and the absence of EOG and EMG channels. I would change this to ICA cleaned.

The proofreading commitment has not been fully met. Section 3.5 still ends with "the utility the utility", the Section 3.5 heading reads "Co-ocurrence", and the abstract contains a leftover insertion reading "the same EEG electroencephalogram (EEG) dataset". Reviewer 6 asked for the abstract abbreviations hvEEGNet, DTW, STFT and SNR to be removed and for past tense in the abstract; the response says these were considered, but the abstract in this version still carries all four abbreviations and remains in the present tense. Please go through the abstract line by line against that reviewer's list.

Finally, on Reviewer 4's request to shorten the manuscript, I understand the pedagogical intent behind retaining the full metric derivations, but declining to remove acknowledged repetition on the grounds that it is intentional is not really a response to the comment. I would not hold up publication on this, and PLOS ONE does not impose a length limit, but a modest tightening of Sections 2.2.3 and 2.2.8 would improve readability.

Reviewer #2: Dear Authors,

Thank you for your comprehensive and diligent revisions. I have carefully reviewed your response to the reviewers' comments and find your explanations and adjustments to be accurate, well-reasoned, and appropriate.

You have successfully addressed all of my previous concerns, significantly improving the overall quality and clarity of the manuscript. I have no further requests for modifications. The manuscript is well-prepared and acceptable for publication in its current form.

Congratulations on your excellent work.

Reviewer #5: The authors have succesfully addressed all of the comments. I wish them good luck with the manuscript! Cheers!

**********

what does this mean?). If published, this will include your full peer review and any attached files.

If you choose “no”, your identity will remain anonymous but your review may still be made public.

**Do you want your identity to be public for this peer review?**  For information about this choice, including consent withdrawal, please see our Privacy Policy

Reviewer #1: **Yes:**  Md Abubakkar

Reviewer #2: No

Reviewer #5: **Yes:**  Khandakar Ashik Shahriar

**********

[NOTE: If reviewer comments were submitted as an attachment file, they will be attached to this email and accessible via the submission site. Please log into your account, locate the manuscript record, and check for the action link "View Attachments". If this link does not appear, there are no attachment files.]

To ensure your figures meet our technical requirements, please review our figure guidelines: https://journals.plos.org/plosone/s/figures

You may also use PLOS’s free figure tool, NAAS, to help you prepare publication quality figures: https://journals.plos.org/plosone/s/figures#loc-tools-for-figure-preparation.

NAAS will assess whether your figures meet our technical requirements by comparing each figure against our figure specifications.

# Author comment: Author response to Decision Letter 2

**DOI:** 10.1371/journal.pone.0361586.r004

5 Oct 2026

We thank the editor and the reviewers for their careful and thoughtful examination of our paper. We appreciate the time and effort they have put into their second reviews. We thank reviewr 2 and reviewer 4 for accepting our previous responses and for their kind words. We also thank reviewer 1 for their additional remarks. All remarks from the first and second round of review have helped us immensely to improve the paper. Please refer to the file Responses_to_reviewers_rev2.pdf (part of the submission), where we provide answers to all new comments. In the new revised version of the manuscript, changes are in blue. In addition to the changes requested by the reviewers, we also made several other changes (not shown in colour) to correct typos and improve readability.

Attachment Submitted filename: Responses_to_reviewers_rev2.pdf

# Editor report: Decision Letter 2

**DOI:** 10.1371/journal.pone.0361586.r005

**Authors:** Onder Aydemir

13 Oct 2026

A self-supervised reconstruction framework for segment-level detection of task-induced deviations in multi-channel EEG

PONE-D-26-16141R2

Dear Dr. Batatia,

We’re pleased to inform you that your manuscript has been judged scientifically suitable for publication and will be formally accepted for publication once it meets all outstanding technical requirements.

Within one week, you’ll receive an e-mail detailing the required amendments. When these have been addressed, you’ll receive a formal acceptance letter and your manuscript will be scheduled for publication.

An invoice will be generated when your article is formally accepted. Please note, if your institution has a publishing partnership with PLOS and your article meets the relevant criteria, all or part of your publication costs will be covered. Please make sure your user information is up-to-date by logging into Editorial Manager at Editorial Manager® and clicking the ‘Update My Information' link at the top of the page. For questions related to billing, please contact billing support.

If your institution or institutions have a press office, please notify them about your upcoming paper to help maximize its impact. If they’ll be preparing press materials, please inform our press team as soon as possible -- no later than 48 hours after receiving the formal acceptance. Your manuscript will remain under strict press embargo until 2 pm Eastern Time on the date of publication. For more information, please contact onepress@plos.org.

Kind regards,

Onder Aydemir

Academic Editor

PLOS One

Additional Editor Comments (optional):

Reviewers' comments:

# Editor report: Acceptance letter

**DOI:** 10.1371/journal.pone.0361586.r006

**Authors:** Onder Aydemir

PONE-D-26-16141R2

PLOS One

Dear Dr. Batatia,

I'm pleased to inform you that your manuscript has been deemed suitable for publication in PLOS One. Congratulations! Your manuscript is now being handed over to our production team.

At this stage, our production department will prepare your paper for publication. This includes ensuring the following:

* All references, tables, and figures are properly cited

* All relevant supporting information is included in the manuscript submission,

* There are no issues that prevent the paper from being properly typeset

You will receive further instructions from the production team, including instructions on how to review your proof when it is ready. Please keep in mind that we are working through a large volume of accepted articles, so please give us a few days to review your paper and let you know the next and final steps.

Lastly, if your institution or institutions have a press office, please let them know about your upcoming paper now to help maximize its impact. If they'll be preparing press materials, please inform our press team within the next 48 hours. Your manuscript will remain under strict press embargo until 2 pm Eastern Time on the date of publication. For more information, please contact onepress@plos.org.

You will receive an invoice from PLOS for your publication fee after your manuscript has reached the completed accept phase. If you receive an email requesting payment before acceptance or for any other service, this may be a phishing scheme. Learn how to identify phishing emails and protect your accounts at https://explore.plos.org/phishing.

If we can help with anything else, please email us at customercare@plos.org.

Thank you for submitting your work to PLOS One and supporting open access.

Kind regards,

PLOS One Editorial Office Staff

on behalf of

Prof. Dr. Onder Aydemir

Academic Editor

PLOS One
